# Intracrine Regulation of Estrogen and Other Sex Steroid Levels in Endometrium and Non-gynecological Tissues; Pathology, Physiology, and Drug Discovery

**DOI:** 10.3389/fphar.2018.00940

**Published:** 2018-09-19

**Authors:** Gonda Konings, Linda Brentjens, Bert Delvoux, Tero Linnanen, Karlijn Cornel, Pasi Koskimies, Marlies Bongers, Roy Kruitwagen, Sofia Xanthoulea, Andrea Romano

**Affiliations:** ^1^GROW–School for Oncology and Developmental Biology, Maastricht University, Maastricht, Netherlands; ^2^Department of Obstetrics and Gynaecology, Maastricht University Medical Centre, Maastricht, Netherlands; ^3^Forendo Pharma Ltd., Turku, Finland

**Keywords:** intracrinology, endometrium, estrogens, lungs, gastrointestinal tract, central nervous system, bone

## Abstract

Our understanding of the intracrine (or local) regulation of estrogen and other steroid synthesis and degradation expanded in the last decades, also thanks to recent technological advances in chromatography mass-spectrometry. Estrogen responsive tissues and organs are not passive receivers of the pool of steroids present in the blood but they can actively modify the intra-tissue steroid concentrations. This allows fine-tuning the exposure of responsive tissues and organs to estrogens and other steroids in order to best respond to the physiological needs of each specific organ. Deviations in such intracrine control can lead to unbalanced steroid hormone exposure and disturbances. Through a systematic bibliographic search on the expression of the intracrine enzymes in various tissues, this review gives an up-to-date view of the intracrine estrogen metabolisms, and to a lesser extent that of progestogens and androgens, in the lower female genital tract, including the physiological control of endometrial functions, receptivity, menopausal status and related pathological conditions. An overview of the intracrine regulation in extra gynecological tissues such as the lungs, gastrointestinal tract, brain, colon and bone is given. Current therapeutic approaches aimed at interfering with these metabolisms and future perspectives are discussed.

## Introduction

The term “intracrinology,” coined in 1988 by prof Labrie, refers to the ability of peripheral tissues to use blood precursors and generate steroids (Labrie, 1991). Several studies have been published but several controversies still exist and relate to the following technical and biological aspects: (a) some intracrine enzymes in peripheral tissues have low expression (300–50,000-times lower than in endocrine glands Stoffel-Wagner, 2001; Murakami et al., 2006, close to the detection limit of standard methods like western blotting and immunohistochemistry -IHC); (b) the technology to robustly quantify steroids (liquid-/gas-chromatography tandem mass-spectrometry -LC-MS or GC-MS), became available during the last 5–10 years only (Rosner et al., 2013); (c) intracrine pathways are highly complex.

This review summarizes our knowledge of intracrinology in peripheral tissues like the endometrium, lungs, gastrointestinal tract (GIT), bone and central nervous system (CNS), with special attention to the metabolism of estrogens. Drug development and potential therapeutic approaches are discussed. In this review, the enzymes involved in steroid deactivation/clearance (Rižner, 2013, 2016; with the exclusion of steroid sulphotransferases) and those involved in the transport of conjugated steroids through the plasma membrane (Rižner et al., 2017) are not described. Studies on serum/tissue steroid levels are reported and discussed only if based on gold standard GC/LC-MS.

## From ovarian estrogen synthesis to intracrinology

**Figure 1:**
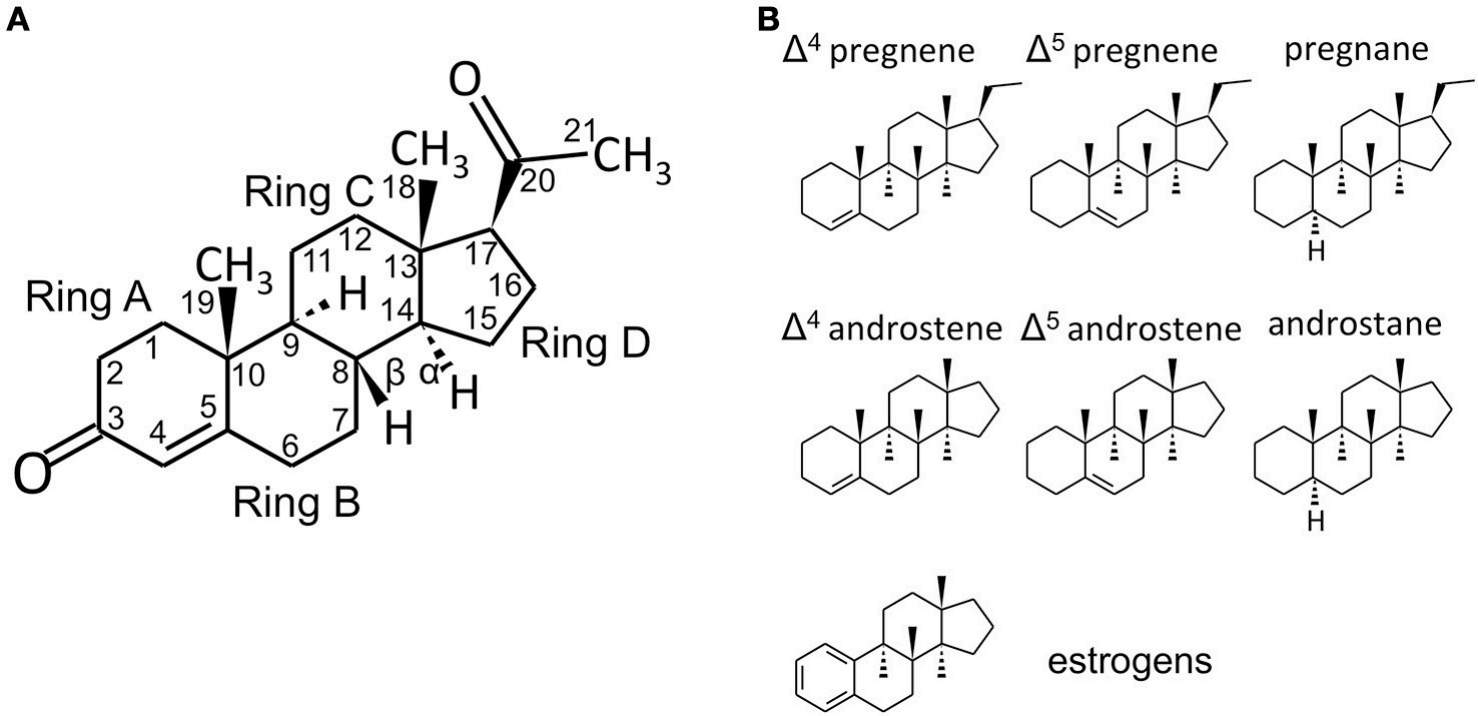
Steroid structure. **(A)** structure of the C21 steroid progesterone (P, used as an example), with carbon numbering and steroid ring numbering. In the storied graphics in Figures 1B and 2, the H groups and the relative bonds will be omitted (with the exclusion of the H in 5α-reduced steroids - androstanes and pregnanes). Methyl groups will be indicated by the bonds only without the CH_3_ group. **(B)** structures of C21 pregnene (Δ^4^ and Δ^5^, i.e., double bond between C4 and C5 or between C5 and C6, respectively), pregnane (5α-reduced steroid), C19 androstene (Δ^4^, Δ^5^) and androstane and C18 (A-ring)-aromatic estrogens. Chemical structures were designed with the aid of Sketcher V2.4 (Ihlenfeldt et al., 2009), available online at PubChem (www.ncbi.nlm.nih.gov; pubchem.ncbi.nlm.nih.gov) (Kim et al., 2016).

**Figure 2:**
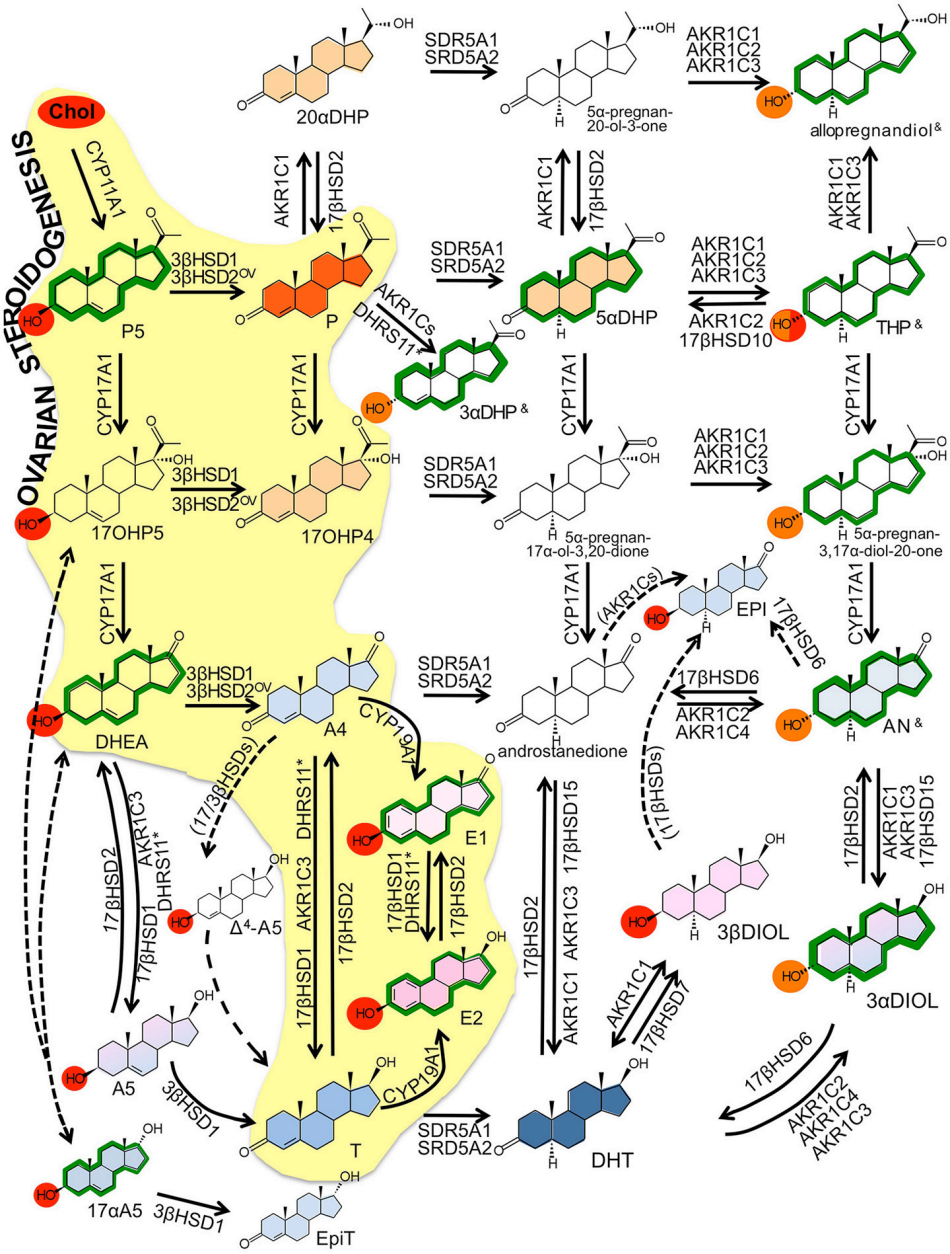
Intracrine networks. Major intracrine networks metabolizing steroids. In this figure, each reaction reports the catalyzing enzymes whose role in that specific reaction is established based on robust evidences (*in vitro, ex vivo, in vivo*). Additional enzymes whose involvement in the same reactions is less robustly demonstrated or based only on *in silico* or cell-free assay are reported in Table 2. The role of 17βHSD3 is disregarded in this figure because restricted to tissues that are not assessed in the present review (testes, prostate, Table 2). Color codes: 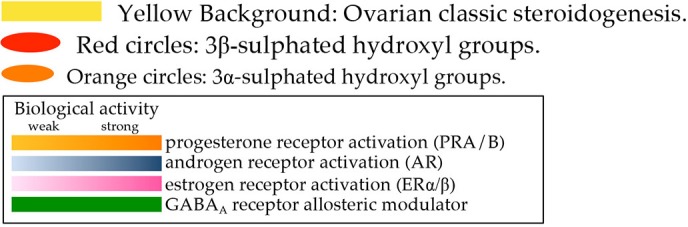 ^OV^ ovarian specific referring to 3β-HSD2 (see text); — dotted arrows indicate reactions that are not fully demonstrated to occur or for which the responsible enzyme is not identified yet; (enzyme name) enzymes indicated by brackets are supposed to catalyze the indicated reaction based on the theoretical assumptions, no experimental proof is yet available; ^&^ these compounds (THP, 3αDHP and allopregnandiol) exist as various hydroxyl α/β isomers (3, 5, 17) with no activity, classic action or neuroactivity (see Table 2); * the role of DHRS11 in steroid metabolism is reported only recently by one publication (Endo et al., 2016).

Local steroid metabolism is possible because those enzymes responsible for steroid synthesis in classical glands (ovaries, adrenals, testes) are expressed in peripheral tissues, where additional and alternative routes for metabolizing steroids are present and make intracrine networks intricate and flexible (Figures 1, 2, Tables 1, 2). In particular, several compounds generated through these pathways, although not being estrogens, can have estrogenic action, because able to bind and activate the estrogen receptors. The biologic activity of the various compounds is given in Table 1, and in Figure 2, by the color codes.

**Table 1:** Major steroidal compounds.

**1. Abbreviation (used here)**		Blood concentration ^**^ (nM)	Biologic activity^***^	Entries^*^
**2. Common name**				
**3. Chemical name**				
1. *none*	PREGNANES	n.d.		CAS:516-59-6
2. *none*				HMDB:60408
3.5α-pregnan-20-ol-3-one^1^				CB:0504265
				ChEBI:81469
1. *none*		3a. M *S*: 28–49^10^	3a. GABA_A_+	
2. allopregnandiol (4 isomers)		3a. F: 0.2–0.7^10^; *S*: 13–20^10^		
3 a. 5α-pregnane-3α,20α-diol ^∧^				a.CAS:566-58-5
b. 5α-pregnane-3β,20β-diol				b.CAS:516-53-0
c. 5α-pregnane-3β,20β-diol				c.CAS:516-53-0
d. 5α-pregnane-3β,20α-diol				d.CAS:516-53-0
1.5αDHP		F: 0.2–1.1^(5, 10)^	PR+ GABA_A_+	CAS:566-65-4
2. allopregnanedione				HMDB:0003759
3.5α-pregnane-3,20-dione				ChEBI:28952
1. *none*		n.d.	CAS:570-59-2	
2. *none*				CB:4441841
3.5α-pregnan-17α-ol-3,20-dione				
1. *none*		n.d.	CAS:6890-65-9	
2. *none*				CB:7269033
3.5α-pregnan-3,17α-diol-20-one ^2^∧^^				
1. THP (isomer 1/4)		All: 0.2–10^6^	GABA_A_+	CAS:516-54-1
2. allopregnanolone^3^		M: 0.1–0.2^10^; *S*: 3.6–10^10^	HMDB:0001449	
3.3α-hydoxy-5α-pregnan-20-one		F: 0.1–0.2^10^; *S*: 4.0–6.7^10^	PC:262961	
1. THP (isomer 2/4)		M *S*: 8.6–18^10^	GABA_A_+	CAS:128-20-1
2. pregnanolone^3^		F: 0.06–0.1^10^; *S*: 5.5–10^10^	HMDB:0062782	
3.3α-hydroxy-5β-pregnan-20-one				CHEBI:1712
				PC:31402
1. THP (isomer 3/4)		M *S*: 7.8–10^10^	GABA_A_−	CAS:516-55-2
2. isopregnanolone^3^		F: 0.08–0.2^10^; *S*: 3.7–7.1^10^	(THP-S)	HMDB:0001455
3.3β-hydoxy-5α-pregnan-20-one				PC:92787
1. THP (isomer 4/4)		All: 0.3–3^6^	CAS:128-21-2	
2. epipregnanolone^3^		M *S*: 0.4–0.6^10^	GABA_A_−	HMDB:0001471
3.3β-hydoxy-5β-pregnan-20-one		F: 0.02–0.04^10^; *S*: 1.5–2.5^10^	(THP-S)	PC:228491
1.3αDHP	PREGNENES	n.d.	GABA_A_+	CAS:25680-68-6
2.3α-dihydroprogesterone				PC:121951
3.4-pregnen-3α-ol-20-one				
1.3βDHP^4^		n.d.	PC:121951	
2.3β-dihydroprogesterone				
3.4-pregnen-3β-ol-20-one				
1. P		M: 0–0.4^6^	PR+	HMDB:01830
2. progesterone		Fpre: 0–80^6^		
3.4-pregnene-3,20-dione		Fpost: 0–0.4^6^		
1.17OHP		M: 0.61–7.0^6^	PR+	CAS:68-96-2
2.17α-hydroxyprogesterone		F: 0.21–2.3^6^	HMDB:0000374	
3.17-hydroxypregn-4-en-3,20-dione				CHEBI:17252
				PC:6238
1.20αDHP	PREGNENES	Fpre: 0.8–11.7^6^	PR+	CAS:145-14-2
2.20α-dihydroprogesterone				HMDB:0003069
3.20α-hydroxypreg-4-en-3-one				PC:8956
1.17OHP5		M: 1.0–12 ^6^	CAS:387-79-1	
2.17-hydroxypregnenolone		F: 0–6.0^6^	HMDB:0000363	
3.5-Pregnen-3β,17α-diol-20-one				CHEBI:28750
				PC:3032570
1. P5		M: 1–15^6^; *S*: 200–1,000^6^	GABA_A_− (P5-S)	CAS:145-13-1
2. pregnenolone		Fpre: 1.0–15^6^; *S*: 100–1,000^6^	HMDB:0000253	
3. pregn-5-en-3β-ol-20-one		Fpost: 1.0–15^6^; *S*: 10–500^6^	CHEBI:16581	
				PC:8955
1. *none*	ANDROSTANES		HMDB:0000899	
2. androstanedione		M: 0.3^7^	CHEBI:22542	
3.5α-androstane-3,17-dione				
1. AN		All: 0.5–0.9^7^	AR+ ^weak^	HMDB:0000031
2. androsterone		M: 0.4–4.0^6,10^; *S*: 617–1,080^10^	GABA_A_+	
3.3α-hydroxy-5α-androstan-17-one		F: 0.3–0.6^10^; *S*: 359–1,070^10^		
1.5βAN		M: 0.09–0.2^10^; *S*: 32–70^10^	GABA_A_+	CAS:53-42-9
2. etiocholanolone		F: 0.1–0.4 ^10^; *S*: 34–88^10^	HMDB:00490	
3.3α-hydroxy-5β-androstan-17-one				CHEBI:28195
				PC:5880
1.3αDIOL		M: 0.4–0.5^9,10^; *S*: 35–121^10^	ERβ+ ^weak^	HMDB:0000495
2. androstanediol		F: 0.03–0.06 ^10^; *S*: 2.4–4.8^10^	GABA_A_+	
3.5α-androstane-3α,17β-diol				
1.3βDIOL		All: 0.15^7^		
2.3β-androstanediol		M *S*: 34–103^10^	ERβ+	HMDB:0000493
3.5α-androstane-3β,17β-diol		F *S*: 8.7–18^10^		
1. EPI		M: 0.3–0.7^9,10^; *S*: 211–532^10^	AR+ ^weak^	CAS:481-29-8
2. epiandrosterone		F: 0.3–0.7^10^; *S*: 172–350^10^		HMDB:0000365
3.3β-hydroxy-17-oxo-5α-androstane				CHEBI:541975
				PC:441302
1. DHT		M: 0/85–3.5 (50–100)^6^	AR+	HMDB:0002961
2. dihydrotestosterone		Fpre: 0.08–1.3^6^		CHEBI:16330
3.17β-hydroxy-5α-androstan-3-one		Fpost: 0.03–1.65^6^		PC:10635
	ANDROSTENES		CAS:481-30-1	
1. EpiT^8^		M: 1.3–2.9^7^	AR+ ^weak9^	HMDB:0000628
2. epitestosterone				CHEBI:42534
3.17α-hydroxy-4-androsten-3-one				CB:10204
				CAS 521-17-5
1. A5		M: 2.6–3.7^9,10^; *S*: 243–494^10^	AR+ ^weak^	HMDB:0003818
2. androstenediol		F: 0.8–1.1^10,11^; *S*: 85–302^10^		CHEBI:2710
3. androst-5-ene-3β,17β-diol				PC:10634
1.17αA5		n.d.	AR+ ^weak^	CAS:521-17-5
2.17α- androstenediol			GAB A_A_−	HMDB:0003818
3. androst-5-ene-3β,17α-diol				CHEBI:2710
				PC:10634
1. Δ^4^-A5		n.d.		CAS:1156-92-9
2.4-androstenediol				HMDB:0005849
3. androst-4-ene-3β,17β-diol				PC:12476620
1. DHEA		M: 10–25^6^; *S*: 2K-10K^6^	GABA_A_−	HMDB:0000077
2. dehydroepiandrosterone		Fpre: 3.0–30^6^; *S*: 1K-8K^6^	(DHEA and DHEA-S)	
3. (3β)-3-hydroxyandrost-5-en-17-one		Fpost: 2.0–20^6^; *S*: 1K-6K^6^		
**1. Abbreviation (used here)**		**Blood concentration** ^**^ **(nM)**	Biologic activity^***^	Entries^*^
**2. Common name**				
**3. Chemical name**				
1. A4	ANDROSTENES	All: 3.1–5.9^7^	AR+	CAS:63-05-8
2. androstenedione				HMDB:0000053
3.4-Androstene-3,17-dione				CHEBI:16422
1. T		M: 5–25^6^		CAS:55-22-0
2. testosterone		Fpre: 0.2–2.0^6^	AR+	HMDB:0000234
3.17β-hydroxyandrost-4-ene-3-one		Fpost: 0.2–1.0^6^		CHEBI:17347
1. E2	ESTROGENS	M: 0.02–0.04^6^		CAS:50-28-2
2.17β-estradiol		Fpre: 0.005–1.0^6^	ER+	HMDB:0000151
3. (17β)-estra-1,3,5(10)-triene-3,17-diol		Fpost: 0.005–0.08^6^		CHEBI:16469
1. E1		M: 0.033–0.1^6^; *S*: 2.0–4.0^6^		CAS:53-16-7
2. estrone		Fpre: 0.015–0.5; *S*: 2.0–5.0^6^	ER+	HMDB:0000145
3.3-hydroxy-1,3,5(10)-estratrien-17-one		Fpost: 0.01–0.12; *S*: 5–20(X10^−^3) ^6^		CHEBI:17263

*List of the steroids discussed in the present review with the major features. Nomenclature of these compounds is variable and aliases are given as **Supplemental Materials***.

* *CAS: Chemical Abstracts Service, a division of the American Chemical Society (www.cas.org. Accessed on date: February 2018); HMDB: Human Metabolome Data Base (www.hmdb.ca. Accessed on date: February 2018) (Wishart et al., 2013); CB: Chemical Book (www.chemicalbook.com. Accessed on date: February 2018); ChEBI (www.ebi.ac.uk/chebi. Accessed on date: February 2018) (Morgat et al., 2015); PC: PubChem (www.ncbi.nlm.nih.gov; pubchem.ncbi.nlm.nih.gov. Accessed on date: February 2018) (Kim et al., 2016)*.

** *M: male subjects; F: female subjects; Fpre: female premenopausal subjects; Fpost: female postmenopausal subjects; -S: sulphated compounds*.

*** *PR, ER, AR: compound activates the indicated steroid receptor; GABA_A_ allosteric positive (+) or negative (–) modulator*.

∧ *This isomer is shown in Figure 2*.

*n.d.: not determined*.

1 *20β-/5β-isomers exist (CB8678436, Chemical Book - www.chemicalbook.com. Accessed on date: February 2018); ^2^Isomer 5α-pregnan-3β,17α-diol-20-one exists (CB:0291774, Chemical Book - www.chemicalbook.com. Accessed on date: February 2018); ^3^In general, 5α-reduced and 3α-hydroxysteroids are positive allosteric modulator of GABA_A_, whereas 3α- and 3β-sulphated hydroxysteroids and 5β-reduced steroids are negative allosteric modulator of GABA_A_. The 3β-hydroxy isomers of THP are inactive (Belelli and Lambert, 2005; Gibbs et al., 2006; ^4^This compound is GABA_A_ receptor inactive; ^5^Pearson Murphy et al., 2001; ^6^Mueller et al., 2015), ^7^Data extracted from the Human Metabolome Data Base-HMDB (www.hmdb.ca. Accessed on date: February 2018) (Wishart et al., 2013); ^8^This compound is strong inhibitor of SRD5As. ^9^Kancheva et al. (2007) and ^10^Bicikova et al. (2013)*.

**Table 2:** Major enzymes involved in steroidogenesis.

	**Chromosome**	Protein (aa)^**^	
**1. Abbr**.	Gene size^*^	**Dalton**	**1. Name 2. Family**
**2. Gene ID**	mRNA size^*^	Localization (L)^***^	3. Catalysis 4. Substrates^****^
**3. *Gene name(s)***	**Exons (no)**		**5. Distribution 6. Cofactor**
**1**. StAR	**Chr:** 8p11.23		**1**. steroidogenic acute regulatory protein
**2**. 6770	**gene:** 8.6	**aa:** 285	**2**. cytochrome P450
**3**. *STAR, STARD1*	**mRNA:** 1.6	**Dalton:** 31,914	**3**. facilitate transport of cholesterol to mitochondria
	**Exons:** 8	**L:** mitochon	**4**. cholesterol
			**5**. restricted (adrenal, testis, ovary, placenta)
**1**. CYP11A1			**1**. cytochrome P450 side-chain cleavage enzyme
**2**. 1583	**Chr:** 15q24.1		**2**. cytochrome P450, type I
**3**. *CYP11A1*; *CYP11A*; *CYPXIA1*; *P450SCC*	**Gene:** 30.0	**aa:** 521	**3**. cleavage of cholesterol side-chain
	**mRNA:** 2.0	**Dalton:** 60,102	**4**. cholesterol**→**P5
	**Exons:** 9	**L:** mitochon	**5**. restricted (adrenal, testis, ovary, placenta)
			**6**. NADP/NADPH
**1**. CYP17A1	**Chr:** 10q24.32		**1**. steroid 17α-hydroxylase/17,20-lyase
**2**. 1586	**Gene:** 6.6	**aa:** 508	**2**. cytochrome P450, type II
**3**. *CYP17A1*; *CPT7*; *CYP17*; *S17AH*; *P450C17*	**mRNA:** 1.9	**Dalton:** 57,371	**3**. 17α-hydroxylase and 17,20-lyase activities
	**Exons:** 8	**L:** EndRet	**4**. P5**→**DHEA; P**→**A4^$^; 5αDHP**→**androstanedione; THP**→** 5α-pregnan-3,17α-diol-20-one
			**5**. restricted (adrenal, testis, ovary, placenta)
			**6**. NADP/NADPH
**1**. CYP19A1	**Chr:** 15q21.1		**1**. cytochrome P450 aromatase
**2**. 1588	**Gene:** 130.6	**aa:** 503	**2**. cytochrome P450, type II
**3**. *CYP19A1; ARO; ARO1; CPV1; CYAR; CYP19; CYPXIX; P-450AROM*	**mRNA:** 1.5–4.5	**Dalton:** 57,883	**3**. oxidative demethylation of C_19_ to C_18_ (aromatisation)
	**Exons:** 10	**L:** EndRet	**4**. A **→** E1; T **→** E2
			**5**. restricted (adrenal, testis, ovary, placenta)
			**6**. NADP/NADPH
**1**. 3βHSD1	**Chr:** 1p11–12		**1**. 3β-hydroxysteroid dehydrogenase/Δ^5 → 4^ isomerase type I
**2**. 3283	**Gene:** 8.1	**aa:** 373	**2**. short chain dehydrogenase/reductase superfamily
**3**. *HSD3B1; HSD3B; HSDB3; SDB3A; (more^∧∧^)*	**mRNA:** 1.7	**Dalton:** 42,252	**3**. oxidative conversion of Δ^5^ 3β-hydroxyl to Δ^4^ keto-steroids
	**Exons:** 4	**L:** membrane	**4**. P5**→**P; 17OHP5**→**17OHP4; DHEA**→**A4; A5**→**T; 17αA5**→**EpiT
			**5**. selectively distributed (placenta, periphery)
			**6**. NADP/NADPH or NAD/NADH
**1**. 3βHSD2	**Chr:** 1p11–13		**1**. 3β-hydroxysteroid dehydrogenase/Δ^5**→**4^ isomerase type II
**2**. 3284	**Gene:** 8.1	**aa:** 372	**2**. short chain dehydrogenase/reductase superfamily
**3**. *HSD3B2; HSDB; HSD3B; SDR11E2*	**mRNA:** 1.7	**Dalton:** 42,052	**3**. oxidative conversion of Δ^5^-3β-hydroxyl to Δ^4^-ketosteroids
	**Exons:** 4	**L:** mitochon	**4**. P5**→**P; 17OHP5**→**17OHP4; DHEA**→**A4
			**5**. restricted (adrenal, testis, ovary)
			**6**. NADP/NADPH or NAD/NADH
**1**. 17βHSD1	**Chr:** 17q11–21		**1**. 17β-hydroxysteroid dehydrogenase type 1
**2**. 3,292	**Gene:** 6.0	**aa:** 328	**2**. short chain dehydrogenase/reductase superfamily
**3**. *HSD17B1*	**mRNA:** 1–2.4	**Dalton:** 34,950	**3**. reduction of 17-keto to 17β-hydroxyl (estrogens)
*E2DH; HSD17; EDHB17; EDH17B2; SDR28C1; (more^∧∧^)*	**Exons:** 6	**L:** cytoplasm	**4. (established)** E1**→**E2; (A4**→**T in rodents) **(postulated)** DHEA**→**A5^1^; P**→**20αDHP^2^;
			DHT**→**3βDIOL^11^; DHT**→** androstanedione^11^
			**5**. selectively distributed (ovary, placenta (low in endometrium, breast)^14,15^
			**6**. NADP/NADPH
**1**. 17βHSD2	**Chr:** 16q24.1–2		**1**. 17β-hydroxysteroid dehydrogenase type 2
**2**. 3294	**Gene:** 63	**aa:** 387	**2**. short chain dehydrogenase/reductase superfamily
**3**. *HSD17B2 HSD17; SDR9C2; EDH17B2*	**mRNA:** 1.5	**Dalton:** 42,785	**3**. oxidation of 17β-hydroxyl to 17-keto (estrogens & androgens)
	**Exons:** 5	**L:** EndRet	**4. (established)** E2**→**E1; T**→**A4; DHT**→**androstanedione^1,10^; 20αDHP**→**P^16^; 5α-pregnan-20-ol -3-one**→**5αDHP^16^; A5**→**DHEA^1^; 3αDIOL**→**AN^5^ **(postulated)** 3αDIOL**→**AN^(1, 10)^; DHT**→**androstanedione^1^; A5**→**DHEA^1^; allopregnandiol**→** THP^16^
			**5**. selectively distributed (liver, intestine, endometrium, placenta, pancreas, prostate, colon, kidney. *Negative* in heart, brain, skeletal muscle, spleen, thymus, ovary, or testis)^(14, 17)^
			**6**. NAD/NADH
**1**. 17βHSD3^&&^	**Chr:** 9q22		**1**. 17β-hydroxysteroid dehydrogenase type 3
**2**. 3293	**Gene:** 67	**aa:** 310	**2**. short chain dehydrogenase/reductase superfamily
**3**. *HSD17B3* *EDH17B3;*	**mRNA:** 1.2	**Dalton:** 34,516	**3**. reduction of 17-keto to 17β-hydroxyl (androgens)
	**Exons:** 11	**L:** EndRet	**4. (established)** A4**→**T **(postulated)** AN**→**3αDIOL^(1, 3, 10)^; androstanedione**→**DHT^(1, 9, 10)^
			**5**. restricted (testis; low in brain, blood, skin, adipose tissue)^14^
			**6**. NADP/NADPH
**1**. 17βHSD4	**Chr:** 5q23.1	**aa:** 736	**1**. 17β-hydroxysteroid dehydrogenase type 4
**2**. 3295	**Gene:** 184	**Dalton:** 79,686	**2**. short chain dehydrogenase/reductase superfamily
**3**. *HSD17B4*	**mRNA:** 2.9	**L:** Peroxisome mitochon	**3**. fatty acid β-oxidation (steroids in pigs)
*DBP; MFE-2; MPF-2; PRLTS1; SDR8C1*	**Exons:** 28		**4. (established)** very long chain branched fatty acids, bile acids **(postulated)** A5**→**DHEA^1^; E2**→**E1^14^
			**5**. ubiquitous (liver, heart, prostate, testis, lung, skeletal muscle, kidney, pancreas, thymus, ovary, intestine, placenta, brain, spleen, colon, lymphocytes)^14^
			**6**. NAD/NADH
**1**. 17βHSD6	**Chr:** 12q13	**aa:** 317	**1**. 17β-hydroxysteroid dehydrogenase type 6
**2**. 8630	**Gene:** 24.5	**Dalton:** 35,966	**2**. short chain dehydrogenase/reductase superfamily
**3**. *HSD17B6*	**mRNA:** 1.6	**L:** EndRet microsomes	**3**. 3α-3β-epimerase; 17β-hydroxyl oxidation (5α-reduced steroids); retinoids
*HSE; RODH; SDR9C*6	**Exons:** 8		**4. (established)** 3αDIOL**→**DHT^10^; AN**→**androstanedione^5^; AN**→**EPI **(postulated)** 3αDIOL**→**AN^5^; E2**→**E1^5^
			**5**. selectively distributed (liver, testis, lung, spleen, brain, ovary, kidney, adrenal, prostate)^14^
			**6**. NAD/NADP
**1**. 17βHSD7	**Chr:** 1q23		**1**. 17β-hydroxysteroid dehydrogenase type 7
**2**. 51478	**Gene:** 22.1	**aa:** 341	**2**. short chain dehydrogenase/reductase superfamily
**3**. *HSD17B7*	**mRNA:** 1.5	**Dalton:** 38,206	**3**. 3-ketosteroid reductase of sterols
*PRAP; SDR37C1*			
	**Exons:** 9	**L:** EndRet	**4. (established)** Sterols/cholesterol biosynthesis; DHT**→**3βDIOL^1^
			**5**. widely distributed (ovary, uterus, placenta, liver, breast, testis, neuronal tissue, adrenal gland, small intestine, prostate, adipose tissue lung, and thymus)^(14, 18)^
			**6**. NADP/NADPH
**1**. 17βHSD8	**Chr:** 6p21.3		**1**. 17β-hydroxysteroid dehydrogenase type 8
**2**. 7923	**Gene:** 2.2	**aa:** 261	**2**. short chain dehydrogenase/reductase superfamily
**3**. *HSD17B8*	**mRNA:** 1.0	**Dalton:** 26,974	**3**. fatty acid elongation; steroid 17βHSD action (rodents).
*KE6; FABG; HKE6; FABGL; RING2; H2-KE6; (more^∧∧^)*	**Exons:** 9	**L:** mitochon	**4. (established)** fatty acids **(postulated)** E2**→**E1
			**5**. widely distributed (prostate, placenta, kidney, brain, cerebellum, heart, lung, small intestine, ovary, testis, adrenal, stomach, liver, adrenals)^14−19^
			**6**. NAD/NADP
**1**. 17βHSD9	**Chr:** 12q23		**1**. 17β-hydroxysteroid dehydrogenase type 9
**2**. 5959	**Gene:** 4.4	**aa:** 318	**2**. short chain dehydrogenase/reductase superfamily
**3**. *HSD17B9*	**mRNA:** 1.4	**Dalton:** 34,979	**3**. retinoid metabolism (steroid metabolism in rodents)
*RDH5; (more^∧∧^)*	**Exons:** 4	**L:** EndRet	**4. (established)** retinoids **(postulated)** AN**→**3αDIOL^1^; androstanedione**→**DHT^1^
**1**. 17βHSD10	**Chr:** Xp11.2		**1**. 17β-hydroxysteroid dehydrogenase type 10
**2**. 3028	**Gene:** 3.1	**aa:** 261	**2**. short chain dehydrogenase/reductase superfamily
**3**. *HSD17B10*	**mRNA:** 0.9	**Dalton:** 26,923	**3**. fatty acids & steroid oxidation; tRNA maturation
*ABAD; CAMR; ERAB; HCD2; MHBD; HADH2; MRPP2; MRX17; MRX31; (more^∧∧^)*	**Exons:** 6	**L:** mitochon	**4. (established)** Isoleucine, fatty acid, bile acid metabolism, THP**→**5αDHP^(20, 34)^ **(postulated)** 3αDIOL**→**AN^1,9^; DHT**→**androstanedione^1,9^; T**→**A4^1^
			**5**. nearly ubiquitous (liver, small intestine, colon, kidney, heart, brain, placenta, lung, ovary, testis, spleen, thymus, prostate, leukocyte)^14^
			**6**. NAD/NADH
**1**. 17βHSD11	**Chr:** 4q22.1		**1**. 17β-hydroxysteroid dehydrogenase type 11
**2**. 51170	**Gene:** 54.9	**aa:** 300	**2**. short chain dehydrogenase/reductase superfamily
**3**. *HSD17B11*	**mRNA:** 1.9	**Dalton:** 32,936	**3**. Short-chain alcohol dehydrogenases
*DHRS8; PAN1B; RETSDR2; SDR16C2; (more^∧∧^)*	**Exons:** 7	**L:** EndRet	**4. (established)** lipids, sec. alcohols/ketones **(postulated)** 3αDIOL**→**AN^1,9^
			**5**. nearly ubiquitous (liver, intestine, kidney, adrenal gland, heart, lung, testis, ovary, placenta, sebaceous gland and pancreas)^14,21^
			**6**. NAD/NADH
**1**. 17βHSD12	**Chr:** 11p11.2		**1**. 17β-hydroxysteroid dehydrogenase type 12
**2**. 51144	**Gene:** 170.1	**aa:** 312	**2**. short chain dehydrogenase/reductase superfamily
**3**. *HSD17B12*	**mRNA:** 2.6	**Dalton:** 34,324	**3**. fatty acid elongation, steroid 17βHSD reductive action (rodents)
*KAR; SDR12C1*	**Exons:** 11	**L:** EndRet	**4. (established)** branched/long chain fatty acids **(postulated)** E1**→**E2
			**5**. ubiquitous (heart, skeletal muscle, liver, kidney, adrenal gland, testis, placenta, brain, pancreas, GIT, trachea, lung, thyroid, prostate, aorta, bladder, spleen, skin, ovary, breast, uterus, vagina)^(14, 22)^
			**6**. NADP/NADPH
**1**. 17βHSD13	**Chr:** 4q22.1		**1**. 17β-hydroxysteroid dehydrogenase type 13
**2**. 345275	**Gene:** 19.1	**aa:** 300	**2**. short chain dehydrogenase/reductase superfamily
**3**. *HSD17B13*	**mRNA:** 2.3	**Dalton:** 33,655	**3. and 4**. unknown
*SCDR9; NIIL497; (more^∧∧^)*	**Exons:** 6	**L:** extracell/EndRet	**5**. restricted (liver; low in bone marrow, lung, ovary, testis, kidney, skeletal muscle brain, bladder)^14^
**1**. 17βHSD14	**Chr:** 19q13.33		**1**. 17β-hydroxysteroid dehydrogenase type 14
**2**. 51171	**Gene:** 23.7	**aa:** 270	**2**. short chain dehydrogenase/reductase superfamily
**3**. *HSD17B14*	**mRNA:** 1.3	**Dalton:** 28,317	**3**. fatty acid & prostaglandin metabolism; 17βHSD activity
*DHRS10; SDR47C1; retSDR3*	**Exons:** 8	**L:** cytoplasm	**4. (established)** fatty acids **(postulated)** 3αDIOL**→**AN^1,9^; E2**→**E1^4^; T**→**A4^4^; A5**→**DHEA^5^
			**5**. widely distributed (brain, liver, placenta, breast)^14^
			**6**. NAD/NADH
**1**. 17βHSD15	**Chr:** 14q24.1		**1**. retinol dehydrogenase 11
**2**. 51109	**Gene:** 19.0	**aa:** 318	**2**. short chain dehydrogenase/reductase superfamily
**3**. *RDH11; PSDR1; ARSDR1; (more^∧∧^)*	**mRNA:** 1.8	**Dalton:** 35,386	**3**. dehydrogenase activity of retinoid and steroids
	**Exons:** 9	**L:** cytoplasm	**4. (established)** retinoids **(post.)** AN**→**3αDIOL^1,9^; androstanedione**→**DHT^1,9^
			**5**. widely distributed **6**. NADP/NADPH
**1**. DHRS11^23^	**Chr:** 17q12		**1**. dehydrogenase/reductase 11
**2**. 79154	**Gene:** 9.0	**aa:** 260	**2**. short chain dehydrogenase/reductase superfamily
**3**. *DHRS11*	**mRNA:** 1.6	**Dalton:** 28,308	**3**. steroid 17HSD & 3βHSD activities; bile acids metabolism
*ARPG836; SDR24C1; spDHRS11*	**Exons:** 7	**L:** extracell	**4**. E1**→**E2; A4**→**T; DHEA**→**A5; androstanedione**→**DHT; AN**→**3αDIOL; P**→**3αDHP^23^ **5**. nearly ubiquitous (testis, small intestine, colon, kidney)^23^ **6**. NADP/NADPH
**1**. AKR1C1	**Chr:** 10p14–15		**1**. aldo-ketoreductase family 1 member C1
**2**. 1645	**Gene:** 20.0	**aa:** 323	**2**. aldo-ketoreductase family
**3**. *AKR1C1*	**mRNA:** 12	**Dalton:** 36,788	**3**. 20αHSD (strong) and 17βHSD (weak) activities; moderate 3-keto reduction to 3β-hydroxyl (> 3α)
*C9, DDH, DDH1, DD1, H-37, HBAB, MBAB HAKRC; DD1/DD2; 2-ALPHA-HSD; 20-ALPHA-HSD*	**Exons:** 9	**L:** cytoplasm	**4. (established)** P**→**20αDHP^6^; 5αDHP**→**5α-pregnan-20-ol-3-one^6^; THP**→**allopregnandiol^6^; DHT**→**3βDIOL^7^; androstanedione**→**DHT^7^; 5α-pregnan-20-ol-3-one**→**allopregnandiol^16^; 5αDHP**→**THP^16^ **(post.)** DHT**→**3αDIOL^7^; A4**→**T^7^; E1**→**E2^7^; 3αDIOL**→**AN^7^; 20αDHP**→** P^7^; DHT**→**3βDIOL^24^; 5α-pregnan-17α-ol-3,20-dione**→**5α-pregnan-3,17α-diol-20-one^16^
			**5**. ubiquitous
			**6**. NADP/NADPH or NAD/NADH
**1**. AKR1C2	**Chr:** 10p14–15		**1**. aldo-ketoreductase family 1 member C2
**2**. 1646	**Gene:** 30.6	**aa:** 323	**2**. aldo-ketoreductase family
**3**. *AKR1C2* *DD; DD2; TDD; BABP; DD-2; DDH2; HBAB; HAKRD; MCDR2; SRXY8; DD/BABP; AKR1C-pseudo*	**mRNA:** 1.3 **Exons:** 9	**Dalton:** 36,735 **L:** cytoplasm	**3**. 20αHSD (weak) and 17βHSD activities; 3-keto reduction to 3α- hydroxyl; bile-acid binding protein activity
			**4. (established)** DHT**→**3αDIOL^7,12^; 5αDHP**→**THP^7^; androstanedione**→**AN^25^; 5α-pregnan-20-ol-3-one**→** allopregnandiol^16^; 5αDHP**→**THP^16^; 5α-pregnan-17α-ol-3,20-dione**→**5α-pregnan-3,17α-diol-20-one^16^ **(postulated)** A4**→** T^7^; E1**→**E2^7^; P**→**20αDHP^7^; 3αDIOL**→** DHT^7^; T**→**A4^7^
			**5**. ubiquitous
			**6**. NADP/NADPH or NAD/NADH
**1**. AKR1C3	**Chr:** 10p14–15		**1**. aldo-ketoreductase family 1 member C3
**2**. 8644	**Gene:** 13	**aa:** 323	**2**. aldo-ketoreductase family
**3**. *HSD17B5* *AKR1C3* *DD3; DDX; PGFS; HAKRB; HAKRe; HA1753; HSD17B5; hluPGFS*	**mRNA:** 1.2 **Exons:** 9	**Dalton:** 36,853 **L:** cytoplasm	**3**. 20αHSD (weak) and 17βHSD activities (androgens); 3-keto reduction to 3α-/β-hydroxyl (weak); 11-ketoprostaglandin reductase^3^
			**4. (established)** A4**→**T^7^; DHT**→**3αDIOL^7^; 3αDIOL**→**AN^7^; 5αDHP**→** THP^7^; 5α-pregnan-20-ol-3-one**→** allopregnandiol^16^; 5αDHP**→**THP^16^; DHEA**→**A5^7^; 5α-pregnan-17α-ol-3,20-dione**→**5α-pregnan-3,17α-diol-20-one^16^; androstanedione**→**DHT^24^ **(postulated)** E1**→**E2^7^;T**→**A4^7^; 20αDHP**→** P^7^
			**5**. nearly ubiquitous (prostate, mammary gland, liver, kidney, lung, heart, uterus, testis, brain, skeletal muscle, adipose tissue, pancreas, hearth, skeletal muscle, thymus, ovary, small intestine and colon)^14,26^
			**6**. NADP/NADPH or NAD/NADH
**1**. AKR1C4	**Chr:** 10p15.1		**1**. aldo-ketoreductase family 1 member C4
**2**. 1109	**Gene:** 25.2	**aa:** 323	**2**. aldo-ketoreductase family
**3**. *AKR1C4*	**mRNA:** 1.2	**Dalton:** 37,067	**3**. 20αHSD (weak) and 17βHSD activities; 3-keto reduction to 3α-hydroxyl (>3β).
*C11; CDR; DD4; CHDR; DD-4; HAKRA;*	**Exons:** 9	**L:** cytoplasm	**4. (established)** DHT**→**3αDIOL^7,10^; androstanedione**→**AN^7,8^; other^$$$^ **(postulated)** A4**→** T^7^; E1,**→**E2^7^; P**→**20αDHP^7^; 3αDIOL**→**DHT^7^
			**5**. restricted (liver) **6**. NADP/NADPH or NAD/NADH
**1**. SRD5A1 **2**. 6715 **3**. *SRD5A1* *S5AR 1*	**Chr:** 5p15.31 **Gene:** 41.0 **mRNA:** 2.3 **Exons:** 5(7)	**aa:** 259 **Dalton:** 29,459 **L:** EndRet	**1**. steroid 5α-reductase 1 **2**. steroid 5α reductase family **3**. androgen and pregnene metabolism **4**. T**→**DHT; A4**→**androstanedione; 17OHP4**→**5α-pregnan-17α-ol-3,20-dione; P**→**5αDHP; 20αDHP**→**5α-pregnan-20-ol-3-one **5**. ubiquitous
**1**. SRD5A2	**Chr:** 2p23.1		**1**. steroid 5α-reductase 2
**2**. 6716	**Gene:** 178.3	**aa:** 254	**2**. steroid 5α reductase family
**3**. *SRD5A2*	**mRNA:** 2.5	**Dalton:** 28,393	**3**. androgen and pregnene metabolism
	**Exons:** 5	**L:** microsomes	**4**. T**→**DHT; A4**→**androstanedione
			**5**. restricted (prostate and androgen sensitive tissues)
**1**. SRD5A3	**Chr:** 4q12		**1**. steroid 5α-reductase 3
**2**. 79644	**Gene:** ….	**aa:** 318	**2**. steroid 5α reductase AND polyprenol reductase subfamily
**3**. *SRD5A3*	**mRNA:** 4.1	**Da:** 36,521	**3**. androgen and pregnene metabolism
*CDG1P; CDG1Q; KRIZI; SRD5A2L; SRD5A2L1*	**Exons:** 6	**L:** EndRet	**4**. T**→**DHT; A4**→**androstanedione; 17OHP4**→**5α-pregnan-17α-ol-3,20-dione; P**→**5αDHP; 20αDHP**→**5α-pregnan-20-ol-3-one
			**5**. ubiquitous
**1**. STS	**Chr:** Xp22.31		**1**. steroid sulphatase
**2**. 412	**Gene:** 208.3	**aa:** 583	**2**. sulphatase
**3**. *STS*	**mRNA:** 6.4	**Dalton:** 65,492	**3**. hydrolyses several 3β-hydroxysteroid sulfates
*ES; ASC; XLI; ARSC; SSDD; ARSC1*	**Exons:** 16	**L:** microsomes EndRet	**4**. sulpho conjugated cholesterol, E1, E2, DHEA, P5, 17OHP5S, A5, EPI
			**5**. ubiquitous (lung, aorta, thyroid, uterus, liver and testis)^27−30^
**1**. SULT1E1	**Chr:** 4q13.3		**1**. estrogen sulphotransferase
**2**. 6783	**Gene:** 50.0	**aa:** 35126	**2**. sulphotransferase 1
**3**. *SULT1E1*	**mRNA:** 1.8	**Dalton:** 35,126	**3**. sulpho-conjugation of steroids
*EST; STE; EST-1; ST1E1; (more^∧∧^)*	**Exons:** 9	**L:** cytoplasm	**4**. E1, DHEA (low affinity for E2)
			**5**. moderately distributed (liver, adrenal, small intestine; low in brain, lung, testis, leukocytes, placenta, salivary gland, stomach, thymus, trachea, uterus, kidney)^(30, 31)^
**1**. SULT2A1	**Chr:** 19q13.3		**1**. dehydroepiandrosterone sulphotransferase
**2**. 6822	**Gene:** 15.9	**aa:** 285	**2**. sulphotransferase 1
**3**. *SULT2A1*	**mRNA:** 2.0	**Dalton:** 33,780	**3**. sulpho-conjugation of steroids, bile acids
*HST; ST2; STD; hSTa; DHEAS; ST2A1;*	**Exons:** 6	**L:** cytoplasm	**4**. DHEA, P5, AN, 17OHP5^32^, A5^32^, AN^33^, EPI, bile acids
			**5**. restricted (liver, adrenal, small intestine (low in colon, hearth, prostate, stomach, testis, thyroid)^30^
**1**. SULT2B1	**Chr:** 19q13.33		**1**. alcohol sulphotransferase
**2**. 6820	**Gene:** 48.5	**aa:** 365	**2**. sulphotransferase 1
**3**. *SULT2B1*	**mRNA:** 1.3	**Dalton:** 41,308	**3**. sulpho-conjugation of steroids
*HSST2; ARCI14*	**Exons:** 7	**L:** cytoplasm	**4**. cholesterol, DHEA
			**5**. moderately distributed (placenta, prostate, lung (low in kidney, salivary gland, small intestine, trachea)^30^
**1**. SULT1A1	**Chr:** 16p11.2		**1**. phenol sulphotransferase 1
**2**. 6817	**Gene:** 18.4	**aa:** 295	**2**. sulphotransferase 1
**3**. *SULT1A1*	**mRNA:** 1.3	**Dalton:** 34,165	**3**. sulpho-conjugation of steroids
*PST; STP; STP1; P-PST; ST1A1; ST1A3; TSPST1; HAST1/HAST2*	**Exons:** 13	**L:** cytoplasm	**4**. E2
			**5**. nearly ubiquitous (adrenal, bone marrow, brain, colon, hearth, kidney, liver, lung, pancreas, leukocytes, placenta, prostate, salivary gland, skeletal muscle, small intestine, spinal cord, spleen, stomach, testis, thymus, thyroid, trachea, uterus)^30^

*List of all enzymes discussed in the present review with the major features. Gene and gene product nomenclature is complex and variable and alias are given as Supplemental Materials. Gene structure (chromosome location, gene length), transcript features (length, exons) and protein characteristics (amino-acid length, molecular weigh and cell localization) were obtained from GeneCards (www.genecards.org. Accessed on date: February 2018) (Stelzer et al., 2016)*.

* *Length in kilo nucleotides*.

** *number of amino-acids*.

*** *Abbreviations: EndRet: endoplasmic reticulum; extracell: extracellular; mitochon: mitochondria*.

**** For some enzymes, substrate specificity based on robust evidences are indicated as ‘established’, whereas other reactions whose catalyzes is supported by less robust experimental evidences (mostly using recombinant proteins in vitro/cell-free assays) are indicated as “postulated.”

$ *This reaction of CYP17A1 does not occur in vivo in humans (Miller and Auchus, 2011)*.

$$$ *AKR1C4 has an important detoxifying function in the liver and converts chlordecone into chlordecone alcohol*.

&& *17βHSD3 is testis specific and the reactions catalyzed by this enzyme are not reported in Figure 2*.

∧∧ *For this gene, additional gene names exist, for details see NCBI database (https://www.ncbi.nlm.nih.gov/)*.

1 *Luu-The and Labrie (2010), Labrie and Labrie (2013), and Labrie (2015); ^2^ Smuc and Rizner (2009), ^3^ Miller et al. (2012b), ^4^ Sivik (2012); ^5^ GeneCards (www.genecards.org. Accessed on date: February, 2018) Stelzer et al. (2016), ^6^ Smuc and Rizner (2009), ^7^ Penning et al. (2004), Steckelbroeck et al. (2010); ^8^ Jin et al. (2011), ^9^Manenda et al. (2016), ^10^Balk and Knudsen (2008), ^11^Gangloff et al. (2003), ^12^Bélanger et al. (2002), ^13^Perez Carrion et al. (1994), ^14^Möller et al. (1999), Moeller and Adamski (2009), ^15^Cornel et al. (2017), ^16^Sinreih et al. (2017b), ^17^Casey et al. (1994), ^18^Törn et al. (2003), ^19^Ohno et al. (2008), ^20^Yang et al. (2016), ^21^Chai et al. (2003), ^22^Sakurai et al. (2006), ^23^Endo et al. (2016). ^24^Rižner and Penning (2014), ^25^Manenda et al. (2016), ^26^Lin et al. (1997), ^27^Miki et al. (2002), ^28^Foster et al. (2008a), ^29^Purohit and Foster (2012), ^30^Mueller et al. (2015), ^31^Marchais-Oberwinkler et al. (2011), Mueller et al. (2015), ^32^Rege et al. (2016), ^33^Strott (2002), and ^34^Yang et al. (2016)*.

### Ovarian steroidogenesis

Transformation of cholesterol to 17β-estradiol (E2) involves first the production of dehydroepiandrosterone (DHEA) in theca cells through the action of steroidogenic acute regulatory protein (StAR) that facilitates the transport of cholesterol into mitochondria, followed by CYP11A1 (rate-limiting) and CYP17A1 (Figure 2); the ovarian pathway is indicated by the yellow background; reviewed by (Miller and Auchus, 2011; Andersen and Ezcurra, 2014). CYP11A1 is a type I CYP localized in mitochondria that uses nicotine-adenine-dinucleotide-phosphate (NADPH) and ferredoxin (Fdx)/ferredoxin reductase (FdR) to cleave the cholesterol side chain and produce pregnenolone (P5). Type II CYP17A1, localized in the endoplasmic reticulum (EndRet), has both 17α-hydroxylase and 17,20-lyase activities. It uses NADPH and P450 oxidoreductase (POR) to first hydroxylate P5 to 17α-hydroxypregnenolone (17OHP5) (17α-hydroxylase action), followed by 17,20-lyase action to release DHEA. Gonad specific type 2 3β-hydroxysteroid dehydrogenase (3βHSD2) has 3β-dehydrogenase and Δ^5^ to Δ^4^ isomerase activities and converts DHEA to androstenedione (A4). Next, CYP19A1 catalyzes the oxidative demethylation of C_19_ androgens to C_18_ estrogens, with A-ring aromatisation; hence A4 is converted to estrone (E1). The final conversion of E1 (with low affinity for the estrogen-receptors -ERs) to E2 (high affinity for ERs and high estrogenic potency) is catalyzed by 17βHSD1 that reduces 17-keto to 17β-hydroxyl steroids. In the ovary, the 17-keto group of A4 can be reduced to 17β-hydroxyl by AKR1C3/17βHSD5 yielding testosterone (T) that is converted to E2 by CYP19A1. Upon ovulation, high 3βHSD2 levels in the corpus luteum lead to high progesterone (P) generation from P5.

### Intracrine steroidogenesis

The expression of StAR, CYP11A1 and CYP17A1 is demonstrated in a limited number of peripheral tissues (see later and **Tables 6**–**8**). However, pregnenes, pregnanes, androstenes and androstanes generated from these initial steps (but also abundantly available as circulating precursors) can be further metabolized locally thus generating a plethora of compounds with various biological activities (estrogenic, androgenic, progestogenic and neuroactive; Tables 1, 2 and Figure 2). The Δ^5^ to Δ^4^ isomerization of androstenes (DHEA, androstenediol -A5- and 17αA5) and pregnenes (P5, 17OHP5) is catalyzed by 3βHSD1, which is the peripheral counterpart of ovarian 3βHSD2. Also 3βHSD2, whose expression was initially considered to be restricted to endocrine tissues, is detected peripherally in recent reports (Stoffel-Wagner, 2001; Tsai et al., 2001; Attar et al., 2009; Huhtinen et al., 2014; Osinski et al., 2018). Due to the high concentration of DHEA (both in blood and tissues), its conversion to A4 by 3βHSDs is relevant to the formation of downstream androgens and of estrogens. Additionally, 3βHSDs convert A5 and the isomer 17αA5 to T and epitestosterone (EpiT). Although minor, in the context of women's health, these pathways are relevant. A5, together with 3α and 3βDIOL (generated by AKR1Cs from DHT and AN, see below) activate both ERs and have estrogenic action (especially 3βDIOL, a potent ERβ binder). A5 possesses immune stimulatory activity whereas its 17α isomer (17αA5) has androgenic, antitumor and neuroactivity. Additionally, EpiT is a weak AR binder and a strong endogenous inhibitor of SRD5As (Loria and Graf, 2012). The endogenous occurrence of 17αA5 is demonstrated in humans (Laatikainen et al., 1971) but its route of synthesis is unclear (Shimizu, 1979). A 17αHSD able to convert A4 to EpiT and DHEA to 17αA5 is characterized in mice (Bellemare et al., 2005) but no human homologous is described yet. Similarly to the ovaries, androgen to estrogen conversion is catalyzed by CYP19A1.

A particularly important reaction is controlled by oxidative and reductive 17βHSDs, which interconvert 17-keto and 17β-hydroxysteroids. Since 17β-hydroxysteroids (T and E2) have higher affinity for the receptors than the keto-steroids (A4 and E1), this balance determines the final androgenic/estrogenic activity. Fourteen 17βHSDs exist, whose specificity is determined by tissue distribution, intracellular localization and biochemistry (Table 2); reviewed thoroughly in (Mindnich et al., 2004; Moeller and Adamski, 2006, 2009; Prehn et al., 2009; Miller and Auchus, 2011). Unpublished data also refer to a 15th 17βHSD (see Table 2; reported in Luu-The et al., 2008) with a putative role in androgen metabolism. With the exclusion of 17βHSD5 (AKR1C3, see below), all other 17βHSDs belong to the short-chain dehydrogenase (SRD) family.

Although all 17βHSDs have been postulated to use steroids as substrates based on cell-free or *in vitro* assays, recent investigations based on substrate specificity (Laplante et al., 2009) and knock-out (KO) models (Table 4) better clarified their roles. Type 1 17βHSD is the estrogenic enzyme and coverts E1 to E2 both in the ovary and in peripheral tissue. Type 2 17βHSD oxidizes 17-hydroxyl groups (E2 and T) to the 17-keto forms (E1 and A4), and possesses also a 20α-hydroxyl oxidative action, through which this enzyme generates P from 20αDHP. Type 6 17βHSD uses 5α-reduced androgens and has 17-hydroxyl oxidative activity (converting androsterone -AN- to androstanedione) and 3-hydroxyl oxidative activity (converting 3αDIOL to the most potent androgen dihydrotestosterone -DHT). Additional catalytic actions for 17βHSD6 (epimerase or 17-hydroxydehydrogenase) are demonstrated *in vitro* (Table 2). Type 14 17βHSD is postulated to have 17β-hydroxyl oxidative action on various steroids, type 7 is involved in cholesterol metabolism as indicated by KO mice (Table 4), whereas there is apparently little/no *in vivo* role of types 8, 9, 10, 11 and 12 17βHSDs on steroid metabolism (Table 2 and indicated by KO mice, Table 4). Recently, a novel SRD, DHRS11, was shown to possess *in vitro* 17-keto to 17β-hydroxyl reductive action (able to use E1, Δ^5^ or Δ^4^ androstenes, androstanes), plus reductive 3βHSD activity toward Δ^4^ pregnenes and other compounds (5β-steroids, bile acids; Table 2 and Figure 2; Endo et al., 2016).

Androgens and progestogens can be further metabolized by aldo-ketoreductases (AKRs) and 5α-reductases (SRD5As; Figure 2). Cytoplasmic AKRs (AKR1C1, 1C2, 1C3/17βHSD5 and 1C4) have broad substrate specificity with non-stereo-selective 3α/3βHSD, 17- and 20-ketosteroid reductase activities (Table 2; Penning et al., 2004; Steckelbroeck et al., 2010). Together with the fact that they have wide tissue distribution (only AKR1C4 is restricted), AKR1Cs contribute to make intracrine networks flexible and intricate (Rižner and Penning, 2014; Sinreih et al., 2014).

SRD5As convert 3-keto Δ^4^ androstene and pregnene to 5α-reduced steroids (androstanes and pregnanes), hence they are important in progestogen, androgen (DHT production) and neurosteroid metabolism (Di Costanzo et al., 2009). SRD5A1 and 3 are widely expressed, in contrast to SRD5A2. Human 5β-reductase activity, catalyzed by AKR1D1, is restricted to the liver, where 5β-steroids are directed to clearance/catabolism. However, some 5β-compounds are neuroactive and recent studies indicate the presence of AKR1D1 in placenta and myometrium (Jin et al., 2011). With the exclusion of their neuroactivity (Paragraph 4.6), 5β-steroids will not be further considered.

The sulphatase pathway is finally responsible for the balance between sulpho-conjugated and free steroids. Sulpho-conjugated steroids (-S) possess higher water solubility, increased stability and longer half-life than unconjugated compounds (e.g., 10–12 h *vs*. 20–30 min for estrogens), and although they cannot bind steroid-receptors, they serve as a reservoir for the formation of biologically active steroids (Reed et al., 2005). Sulphotransferases (SULTs) are phase-I detoxifying enzymes that use bis-phospho-nucleotide 3′-phospho-adenosine-5′-phosphate- (PAP)-sulfate as donor to conjugate 3β-hydroxyl steroids (e.g., estrogens, DHEA, P5, cholesterol; red circles in Figure 2) with a sulfate group (Strott, 2002; Rižner, 2016). Distinct SULTs have different specificities toward substrates, with SULT1E1 being the major estrogen sulphating enzyme (with little contribution of SULT1A1), and SULT2A1 being specific for DHEA (but also for P5, 17OHP5 and A5) (Table 2). Steroid sulphatase (STS) is a membrane-bound microsomal enzyme that catalyzes the hydrolysis of sulfate ester bonds from sulphated-steroids (cholesterol-S, P5-S, 17OHP5-S, DHEA-S, E1-S) (Mueller et al., 2015; Rižner, 2016), thus releasing unconjugated compounds.

Although sulphated-3α-hydroxysteroids are not thoroughly studied, they are detected in biospecimens (AN-S, 3αDIOL-S; Table 1 and orange circles in Figure 2). They are most likely produced by SULT2A1 (active on 3α-hydroxy bile acids) (Strott, 2002; Rižner, 2016) but no 3α-stereo specific sulphatase is known to date. Some intracellular sulphated-steroids are converted to other compounds without prior desulphation (Sánchez-Guijo et al., 2016).

In conclusions, intracrinology presents redundant and complex pathways, which generate compounds with various activities. Genetic variants in intracrine genes are associated with various diseases (classically endocrine and not; Table 5). Even in the absence of the enzymatic machinery to metabolize cholesterol (StAR, steroidogenic factor, CYP17A1 and CYP11A1), DHEA, P5 and especially their sulphated-conjugates have high blood concentrations (Table 1), and are used to generate all other steroids in peripheral tissues.

## Drug development

Natural hormones have been historically used as drugs, and depending on definitions, approximately 90 marketed drugs share a steroidal core (see https://www.drugbank.ca). Steroids (T, E2, cortisol, DHEA), simple derivatives (ethinylestrogen, prednisolone) or more complex analogs (abiraterone, fulvestrant) are used in various conditions. This old-and-proven steroidal chemistry based approach is used even in modern era.

By targeting steroid intracrine metabolism, the effects of steroids can be modulated locally. Table 3 overviews the available drugs targeting intracrine enzymes and their developmental status. CYP19A1 (aromatase) inhibitors, currently at their third generation, started to be used for breast cancer during the 80's of last century (Lønning and Eikesdal, 2013), and was followed by drugs able to target other enzymes (CYP11A1, CYP17A1, SRD5As; Table 3).

**Table 3:** Drugs targeting intracrine enzymes.

	**Inhibitor name (if known); (Drug Bank ID**^**#**^**)**				
	**Developmental phase**^**&**^			**Approved drugs**	
**Name**	**Discovery**	**Preclinical indication**	**Clinical indication**	**Inhibitor name**	**Indication**
CYP11A1	✓	✓	✓	Aminoglutethimide (DB00357^##^)	Cushing's syndrome Breast cancer
CYP17A1	✓	✓	✓	Abiraterone (DB05812)	Prostate cancer, metastatic, castration-resistant
CYP19A1	✓	✓	Letrozole (DB01006)^(19, 22, 24)^	Anastrozole	Breast cancer: adjuvant treatment, metastatic
				(DB01217)	
				Letrozole	
				(DB01006)	
				Exemestane	Breast cancer, palliative
				(DB00990)	
				Formestane^1^	
				Testolactone^*^	
				(DB00894)	
			Anastrozole (DB01217) ^(20, 23)^		
			Exemestane (DB00990) ^21^		
			Advanced stage endometrial cancer, NSCLC^∧^, LAM^∧∧^		
3βHSD1	✓	✓	✓	Trilostane* (DB01108)	Cushing's syndrome (veterinary use)
3βHSD2	✓	✓	✓	Trilostane* (DB01108)	Cushing's syndrome (veterinary use)
17βHSD1	✓	Endometriosis^2,3^			
		Endometrial cancer^4^			
		Breast cancer^5,6^			
		Endometrial hyperplasia^7^			
17βHSD2	^8,9^				
17βHSD3^&&^		Prostate cancer^10^			
17βHSD7	^11^				
AKR1C1	^12^				
AKR1C2	^12^				
AKR1C3	✓	✓	ASP-9521^13^ Prostate cancer^**^		
			BAY 1128688 Endometriosis^***^		
AKR1C4	^12^				
SRD5A1	✓	✓	✓	Dutasteride (DB01126)	Prostatic hyperplasia (benign)
SRD5A2	✓	✓	✓	Finasteride (DB01216)	Prostatic hyperplasia (benign)
				Dutasteride (DB01126)	
SRD5A3	✓	✓	✓	Dutasteride (DB01216)	Prostatic hyperplasia (benign)
STS	✓	✓	Irosustat (DB02292)		
			Endometrial cancer ^14,15^		
			Breast cancer^14−17^		
			E2MATE/PLG2001		
			Endometriosis^18^		
SULT1E1	✓	✓	✓	Cyclizine (DB01176)	antistaminic for nausea/vomiting

& *‘Clinical phase’, i.e., in phase I, II or III trial; ‘Preclinical phase’ refers to in vivo testing; ‘Discovery phase’ any previous phase with some candidate compounds*.

# *Drug Bank ID if the compound is deposited in Drug Bank database (www.drugbank.ca/drugs. Accessed on date: February 2018) (Wishart et al., 2018)*.

## *Aminoglutethimide (ID: DB00357) is an important inhibitor of CYP11A1 with inhibitory activity on CYP19A1 as well*.

* *No longer on the market*.

&& *17βHSD3 is testis specific and the reactions catalyzed by this enzyme are not reported in Figure 2*.

** *This trial for prostate cancer was prematurely terminated (www.clinicaltrials.gov, NCT01352208)*.

*** *Phase I trial is concluded (www.clinicaltrials.gov, NCT02434640. Accessed on date: February 2018) and a phase II trial started 2016 (http://adisinsight.springer.com/drugs/800041929)*.

∧ NSCLC: non-small cell lung cancer

∧∧ LAM: lymphangioleiomyomatosis

1 *Perez Carrion et al. (1994), ^2^Arnold and Einspanier (2013), ^3^Delvoux et al. (2014), ^4^Konings et al. (2017), ^5^Järvensivu et al. (2018), ^6^Husen et al. (2006), ^7^Saloniemi et al. (2010), ^8^Gargano et al. (2015), ^9^Soubhye et al. (2015) ^10^Day et al. (2013), ^11^ Wang et al. (2017), ^12^BroŽic et al. (2011), ^13^ Kikuchi et al. (2014), ^14^Purohit and Foster (2012), ^15^Pautier et al. (2017), ^16^Palmieri et al. (2017a), ^17^Palmieri et al. (2017b), ^18^Pohl et al. (2014), ^19^Ma et al. (2004), ^20^Rose et al. (2000), ^21^Lindemann et al. (2014), ^22^Slomovitz et al. (2015), ^23^ NCT00932152; ^25^Lu et al. (2017)*.

**Table 4:** Mouse models (knockouts - KO or transgenic-TG, i.e., ubiquitous expression of the gene, unless specified) for intracrine enzymes.

**Gene^∧^**	**Modification MGI ID^$^**	**Phenotype**
SatAR	Null/KO^1^ MGI: 2388706	**Endocrine (steroids) & reproductive endocrinology** - abnormal endocrine organs (adrenal, ovaries, prostate, testis). - decreased steroids and increased adrenocorticotropin level. - adrenocortical insufficiency. - loss of negative feedback regulation at hypothalamic-pituitary levels.
		**Additional** Growth retardation neo/post natal lethality (incomplete penetrance). ***Reproductive system***: abnormal uterus; incomplete spermatogenesis; abnormal genitalia.
CYP11A1	Null/KO^2^ MGI:5464022	**Endocrine (steroids) and reproductive endocrinology** - abnormal adrenal gland morphology. - increased circulating adrenocorticotropin level. - lack of steroid production. - decreased corticosterone and aldosterone levels.
	Null/KO^3^ MGI: 2183813	**Additional** Neonatal lethality (rescued by steroid supplementation); abnormal mitochondrion morphology; abnormal lipid level. ***Reproductive system***: abnormal genitalia, prostate, testis morphology and spermatogenesis; ***Nervous system***: abnormal adrenaline and noradrenaline level; abnormal food intake, hypoactivity; postnatal growth retardation.
CYP17A1	Null/KO^4^ MGI:3722780	**Endocrine (steroids) & reproductive endocrinology** - increased circulating cholesterol level. - decreased T level. - early reproductive senescence.
	Null/KO^5^ MGI:3047328 Null/KO MGI:5605834	**Additional** Homozygous embryonic lethality (Ed7, between implantation and somite formation). ***Reproductive system***: abnormal sperm flagellum morphology/asthenozoospermia; reduced male fertility. ***Bone:*** abnormal bone structure, mineral content and density. ***Metabolism:*** increased total body fat; decreased lean body mass; increased circulating creatinine level; increased fasted circulating glucose level. ***Nervous system***: abnormal sexual interaction.
CYP19A1	Null/KO^6^ MGI:2179439	**Endocrine (steroids) and reproductive endocrinology** - increased circulating cholesterol, T, DHT, FSH, LH and prolactin. - decreased circulating E2 level. - abnormal endometrium (thin, decreased uterus weight). - abnormal ovary (absence of follicles and corpus luteum, anovulation).
	Null/KO^7^ MGI:2154536 Null/KO^8^ MGI:2389548	**Additional** ***Reproductive system***: ovary hemorrhage and cysts; increased seminal vesicle weight and abnormal seminiferous tubule epithelium and oligozoospermia; female infertility and reduced male fertility. ***Metabolism:*** increased fat; obesity and susceptibility to weight gain. ***Bone:*** decreased bone mineral density and bone mass; increased bone resorption, osteoclast cell number; abnormal compact and trabecular bone morphology. ***Metabolism:*** increased circulating glucose and triglyceride levels; impaired glucose tolerance; insulin resistance; hepatic steatosis; abnormal liver physiology. ***Nervous system***: abnormal short term spatial reference memory; abnormal emotion/affect behavior; abnormal barbering behavior; increased grooming behavior; abnormal locomotor activation, bradykinesia; abnormal mating frequency.
17βHSD1	Null/KO^9^ MGI:5576042 and 3799948	**Endocrine (steroids**) **& reproductive endocrinology** - abnormal corpus luteum morphology and decreased number. - increased ovarian E1:E2 and A4:T ratios. - increased LH level. - reduced P level.
		**Additional** Increased circulating alkaline phosphatase level, pigmentation, abnormal retinal pigmentation, abnormal lens morphology, abnormal retina morphology, abnormal retinal pigmentation. ***Reproductive system***: increased ovary weight; reduced female fertility. ***Metabolism:*** decreased circulating glucose level. ***Nervous system***: abnormal behavior, response to light, sleep behavior, decreased exploration in new environment; abnormal motor coordination/balance.
17bHSD1	TG^10^	**Reproductive endocrinology** - female have increased T levels. - increased E1 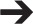 E2 conversion. - masculinization in females. - develop benign/malignant breast, ovarian and endometrial conditions.
17βHSD2	Null/KO^11^ MGI:3773836	**No clear reproductive endocrinology phenotype** **Additional** Heterozygous mice: growth retardation at birth ant postnatal; premature death; renal degeneration. ***Reproductive system***: 70% embryonic lethality (Ed11.5) due to placental defects (homozygous); small and abnormal placenta morphology; ***Nervous system***: brain phenotype with enlarged ventricles; abnormal cortex morphology; impaired balance, coordination, abnormal sleep pattern, megacephaly.
	TG^12^	**Reproductive endocrinology** - low T level.
		**Additional** Growth retardation; delayed eye opening; impaired retinoic signaling. ***Reproductive system***: disrupted spermatogenesis. ***Bone:*** decreased bone formation (pre-pubertal age); decreased IGF-I and osteocalcin levels.
17βHSD4	Null/KO^13^	**No clear reproductive endocrinology phenotype** **Additional** Neonatal and postnatal lethality; postnatal growth retardation; abnormal mitochondrion morphology; abnormal bile salt level; hepatic steatosis. ***Reproductive system***: abnormal testis and spermatid morphology; seminiferous tubule degeneration; small testis; abnormal gametogenesis; reduced male fertility. ***Nervous system***: microgliosis; Purkinje cell degeneration; astrocytosis; axon degeneration; abnormal suckling behavior; increased anxiety-related response, tremors, ataxia, impaired coordination, hypoactivity, lethargy; abnormal gait. ***GIT:*** abnormal intestinal absorption. ***Metabolism:*** decreased body weight; abnormal lipid homeostasis and decreased fatty acid level.
17βHSD7	Null/KO^14^ MGI:3811923	**Endocrine (steroids)** Cholesterol biosynthesis.
	Null/KO^15^ MGI:4456868	**Additional** Decreased embryo size; embryo lethality due to heart malformations (Ed10.5); abnormal blood vessel and capillary morphology. ***Nervous system***: brain malformations; forebrain hypoplasia; increased neural tube apoptosis.
17βHSD9	Null/KO^16^ MGI: 2446073 Null/KO^17^ MGI:2388375	**No clear reproductive endocrinology phenotype** **Additional** Visual defects; abnormal eye electrophysiology, delayed dark adaptation.
17βHSD10	Null/KO^18^	**No clear reproductive endocrinology phenotype** **Additional** Mitochondria dysfunction; reduced plasma glucose and increase insulin levels. ***Nervous system***: neuronal damage.
	TG (brain specific)^19^	**No clear reproductive endocrinology phenotype** **Additional** ***Nervous system***: Protect against ischemia, Parkinson, Alzheimer disease model
17βHSD11	Null/KO^20^ MGI:5581418	**No clear reproductive endocrinology phenotype** **Additional** Increased total circulating protein level. ***Nervous system***: hyperactivity.
17βHSD12	Null/KO^21^	**No clear reproductive endocrinology phenotype** **Additional** Embryo lethality Ed 9.5; impaired organogenesis; reduced arachidonic acid synthesis. ***Reproductive system***: ovarian dysfunction, fertility problems, smaller litters, significantly fewer numbers of ductal branches than wild type female mammary glands; ovulation problems. ***Nervous system***: high embryo expression in neuronal structures.
17βHSD13	Null/KO^22^ MGI:5007180	No clear phenotype associated.
17βHSD14	Null/KO^23^ MGI:5007181	**No clear reproductive endocrinology phenotype** **Additional** Increased IgG2a level. ***Reproductive system***: oligozoospermia, testis degeneration, male infertility. ***Nervous system***: increased response to stress-induced hyperthermia.
17βHSD15	Null/KO^24^ MGI:3526658 & 3586379	**No clear reproductive endocrinology phenotype** **Additional** Abnormal eye electrophysiology, delayed dark adaptation
AKR1C3/ 17βHSD5^*^	Null/KO^25^ MGI:3527218	**Reproductive endocrinology** - long gestation, parturition failure. - increased levels of P. - prolonged estrous and diestrous.
	Null/KO^26^ MGI:3774264	**Additional** Small litter size, the number of pups, especially live pups, was markedly decreased hematopoietic system phenotype. ***Nervous system***: Some behavioral phonotype,
SRD5A1	Null/KO^27^ MGI:1857454	**Reproductive endocrinology** **-** parturition defects, rescued by 3α-DIOL supplementation.
		**Additional** Decreased litter size; small prostate.
SRD5A2	Null/KO^28^ MGI:2178039	**Reproductive endocrinology** **-** T accumulation in reproductive tissues. - impaired androgen-dependent gene expression. **-** parturition defects, rescued by 3α-DIOL supplementation.
		**Additional** Decreased litter size; small prostate.
SRD5A3	Null/KO^29^ MGI:5520177	***Mouse not thoroughly characterized*** Embryonic lethality, abnormal heart morphology, abnormal neural tube closure
SULT1E1	Null/KO^30^ MGI:3529586	**Reproductive endocrinology** - elevated circulating estrogen levels.
		**Additional** Disturbed platelet physiology. ***Reproductive system***: leyding cell hyperplasia and abnormal morphology; abnormal testis morphology; abnormal placentation and amniotic fluid composition.
SULT2B1	Null/KO MGI:5432568 (unpublished)	**Endocrine (steroids)** disturbed cholesterol metabolism and levels.

∧ *No report/references was found for 17βHSD3, 17βHSD6, 17βHSD8, 3βHSD1, 3βHSD2, DHRS11, STS, SULT2A1, SULT1A1*.

* *The human AKR1C3/17βHSD5 KO refers to mice with disrupted AKR1C18, however, functional conservation between the four human AKR1Cs and the eight mouse AKR1Cs in unclear (Sudeshna et al., 2013)*.

$ *Reference ID refers to the Mouse Genome Informatics (MGI; www.informatics.jax.org. Accessed on date: February 2018) (Blake et al., 2017)*.

1 *Caron et al. (1997), ^2^Huang et al. (2012), ^3^Hu et al. (2002), ^4^Liu et al. (2005), ^5^Bair and Mellon (2004), ^6^Nemoto et al. (2000), ^7^ Fisher et al. (1998), ^8^Honda et al. (1998), ^9^Hakkarainen et al. (2015), ^10^Saloniemi et al. (2010) and Järvensivu et al. (2018), ^11^Rantakari et al. (2008), ^12^Zhongyi et al. (2007), ^13^Baes et al. (2000), ^14^Shehu et al. (2008), ^15^Jokela et al. (2010), ^16^Shang et al. (2002), ^17^Driessen et al. (2000) and Sahu et al. (2015); ^18^Li et al. (2010) and Rauschenberger et al. (2010); ^19^Li et al. (2010); Rauschenberger et al. (2010), ^20^Dickinson et al. (2016), ^21^Rantakari et al. (2010); Kemilainen et al. (2016); ^22^Tang et al. (2010), ^23^Tang et al. (2010), ^24^Kim et al. (2005), ^25^Piekorz et al. (2005), ^26^Ishida et al. (2007), ^27^Mahendroo et al. (1996), ^28^Mahendroo et al. (2001), ^29^Dickinson et al. (2016), ^30^Qian et al. (2001) and Tong et al. (2005)*.

**Table 5:** Diseases associated with gene variants in intracrine enzymes.

**Name**	**Affected system or tissue *Disease***	**Phenotype**	**OMIM ID^#^ References**
StAR	**Endocrine system** *Lipoid adrenal hyperplasia*	Deficiency of adrenal or gonadal steroids	OMIM: 201710^1^
		All individuals are phenotypic females	
		Infant mortality (mineral- & glucocorticoid deficiency	
CYP11A1	**Endocrine system** *Congenital adrenal insufficiency, with 46XY sex reversal*	Acute adrenal insufficiency in infancy or childhood	OMIM: 613743^1^
		Abnormality of cholesterol metabolism	
		Absence of secondary sex characteristics	
	**Reproductive** (*ass^∧^*)	SNPs^∧∧^ associated with endometrial cancer	^2^
	**Bone** (*ass^∧^*)	SNPs associated with skeletal adverse events to AI	^3^
	**CNV** (*ass^∧^)*	SNPs associated with neurological disturbances	^4^
CYP17A1	**Endocrine system** *Congenital adrenal insufficiency (17α-hydroxyl. deficiency)*	Excessive corticosteroids leading to hypertension Low aldosterone synthesis Abnormal sex determination and secondary sex characteristics, amenorrhea	OMIM: 202110^1^
	**GIT** *(ass^∧^)*	SNPs associated with risk of cancer in the CRC^##^	^5^
	**Reproductive** *(ass^∧^)*	SNPs associated with endometrial cancer	^6^
CYP19A1	Reprod Endocrin^**^Aromatase deficiency	Pseudo hermaphroditism in female	OMIM: 613546
		Cystic ovaries, delayed bone maturation, adiposity	
	*Aromatase excess syndrome*	Heterosexual precocity and gynecomastia in males	OMIM: 139300
		Isosexual precocity in females	
	**Reproductive** *(ass^∧^)*	SNPs associated with endometrial cancer and ovarian cancer risks, endometriosis risk and risk to develop preeclampsia	_6−14_
	**Bone** *(ass^∧^)*	SNPs associated with osteoporosis and fracture risk	_15, 16_
	**Lung** *(ass^∧^)*	SNPs associated with lung cancer	^17^
	**GIT** *(ass^∧^)*	SNPs associated with gastric cancer	^18^
3βHSD1	Endocr syst^*^(ass^∧^)	SNPs associated with hypertension	^19^
	**Skin** *(ass^∧^)*	SNPs associated with acne susceptibility	^20^
3βHSD2	**Endocrine system & Reprod Endocrin** *Congenital adrenal insufficiency (3β-HSD2 deficiency)*	Impaired steroid biosynthesis	OMIM: 201810^1^
		Low cortisol, aldosterone, P androgens, estrogens.	
		Male new-borns exhibit pseudo hermaphroditism	
		Incomplete masculinization of the external genitalia	
		Affected females can have partial verification	
	**Urogenital** *(ass^∧^)*	SNPs associated with bladder cancer	^21^
17βHSD1	**Reproductive** *(ass^∧^)*	SNPs associated with E2 levels and with endometriosis, cancer risk, abortion	_22−25_
17βHSD3	**Reprod Endocrin** *ketosteroidreductase deficiency of testis*	Males: pseudo hermaphroditism, gynecomastia	OMIM: 264300^1^
		Infertility	
17βHSD4	**CNS and** **Reprod Endocrin** *Perrault syndrome 1*	Ovarian dysgenesis, amenorrhea, low estrogens	OMIM: 233400
		Sensorineural deafness,	
		Neurologic manifestations (mild mental retardation)	
	**CNS** *D-bifunctional protein deficiency*	Abnormal peroxisomal fatty acid beta-oxidation	OMIM: 261515
		Deterioration of nervous system functions	
		Infantile-onset of hypotonia, seizures, and abnormal facial features, death before the age of 2 years	
17βHSD6	**Reproductive** *(ass^∧^)*	SNPs associated with PCOS^***^	^26^
17βHSD9	**Eye** *Fundus albipunctatus*	Fleck retina disease, night blindness, delayed cone and rod photopigment regeneration.	OMIM: 136880
17βHSD10	**CNS** *HSD10 mitochondrial disease*	X-linked neurodegenerative disorder	OMIM: 300438
		Multisystemic features, mitochondrial dysfunction	
	*Turner type X-linked mental retardation*	Moderate to profound mental retardation Macrocephaly and variable skeletal features	OMIM: 300706
17βHSD12	**Reproductive** *(ass^∧^)*	(Male) SNPs associated with prostate cancer	^27^
	**Lung** *(ass^∧^)*	SNPs associated with pulmonary function	^28^
17βHSD15	**Eye and CNS** *Retinal dystrophy, juvenile cataracts, short stature*	Decreased visual acuity, retinitis pigmentosa	OMIM: 616108
		Psychomotor delays from early childhood, lack of fine motor skills and coordination, learning difficulties, facial dysmorphism	
AKR1C1	**Lymphocytes** *(ass^∧^)*	SNPs associated with non-Hodgkin lymphoma	^29^
	**Lung** *(ass^∧^)*	SNPs associated with lung cancer	^29^
	**CNS** *(ass^∧^)*	SNPs associated with panic disorders	^29^
AKR1C2	**Reprod Endocrin** *46XY sex reversal 8*	Males: ambiguous external genitalia, cryptorchidism	OMIM: 614279
		Disturbed endocrine features	
	**Endocr. syst**. *(ass^∧^)*	SNPs associated with weight-gain predisposition	^29^
	**Lung** *(ass^∧^)*	SNPs associated with lung cancer	^29^
AKR1C3	**Reproductive** *(ass^∧^)*	SNPs associated with T levels and PCOS	^29,30^
	**Lymphocytes** *(ass^∧^)*	SNPs associated with large B cell lymphoma	^29^
	**Lung** *(ass^∧^)*	SNPs associated with lung cancer	^29^
	**Leukocytes** *(ass^∧^)*	SNPs associated with lung childhood leukemia	^29^
	**Urogenital** *(ass^∧^)*	SNPs associated with bladder cancer	^29^
	**CNS** ***(ass**^∧^**)***	SNPs associated with amyotrophic lateral sclerosis	^29^
AKR1C4	**Reprod Endocrin** *46XY sex reversal 8*	Males: ambiguous external genitalia, cryptorchidism	OMIM 614279
		Disturbed endocrine features	
	–* (ass^∧^)*	SNPs associated with responses to anthracycline	^29^
	**CNS** *(ass^∧^)*	SNPs associated with paranoia risk	^29^
SRD5A1	**Reproductive** *(ass^∧^)*	Haplotypes associated with PCOS and hirsutum	^31^
SRD5A2	**Reprod Endocrin** *Pseudovaginal perineoscrotal hypospadias*	Males: pseudo hermaphroditism, ambiguous genitalia, cryptorchidism, small prostate	OMIM: 264600
		No Mullerian structures, masculinization at puberty	
		No breast development or menstruation at puberty	
		Abnormal plasma DHT (and T) level	
	**Reproductive** *(ass^∧^)*	Haplotypes associated with PCOS	^31^
	**Bone** *(ass^∧^)*	SNPs associated with low bone mineral density	^32^
SRD5A3	**CNS** *Type Iq congenital glycosylation disorder*	Developmental delay, midline brain malformations	OMIM: 612379
		Variable extents of visual loss	
	**CNS** *Kahrizi syndrome*	Mental retardation, delayed motor development, speech impairment, coarse facial features	OMIM: 612713
STS	**Skin** *X-linked ichthyosis*	Cutaneous manifestations: dark brown, polygonal scales and generalized dryness	OMIM: 308100^40^
	**Bone** *bone dysplasia*	Chondrodysplasia punctata and bone dysplasia	^33^
SULT1E1	**Reproductive** *(ass^∧^)*	SNP associated with estrogen dependent diseases	^34^
SULT2A1	**Reproductive** *(ass^∧^)*	SNP associated with DHEA-S, androgens and PCOS	^40,35^
SULT2B1	**Skin** *congenital autosomal recessive ichthyosis*	Generalized desquamation, dry scaly skin, hyperkeratosis, erythema	OMIM: 604125
SULT1A1	**Reproductive** *(ass^∧^)*	SNPs associated with endometrial cancer	^11,36^
	**Bone** *(ass^∧^)*	SNPs associated with low bone mineral density	^32^
	**GIT** *(ass^∧^)*	SNPs associated with risk of cancer in the GIT	^37−38^
	**–** *(ass^∧^)*	SNPs associated with activity and termostability	^37,39^

*Selected papers reporting association between SNPs and diseases are reported. Association studies with enzymes involved in steroid signaling but not discusses in the present review exist (for some references, see Doherty et al., 2005; Freedman et al., 2009; Miller and Auchus, 2011; Mueller et al., 2015)*.

* *Endocr syst: Endocrine system*.

** *Reprod Endocrin: Reproductive endocrinology*.

*** *PCOS: polycystic ovarian syndrome*.

∧ *ass: association studies, case controls*.

∧∧ *SNP: single nucleotide polymorphism*.

# *OMIM: Online Mendelian Inheritance in Man. McKusick-Nathans Institute of Genetic Medicine, Johns Hopkins University (Baltimore, MD). (https://omim.org/. Accessed on date: February 2018)*.

## *CRC: colorectal cancer*.

1 *Miller and Auchus (2011), ^2^Terry et al. (2010), ^3^Rodríguez-Sanz et al. (2015), ^4^Deng et al. (2016), ^5^Zeng et al. (2016), ^6^Olson et al. (2007), ^7^Berstein et al. (2006), ^8^Kitawaki et al. (2002), ^9^Lundin et al. (2012), ^10^Thompson et al. (2016), ^11^Gulyaeva et al. (2008), ^12^Setiawan et al. (2009), ^13^Zacher et al. (2016), ^14^Shimodaira et al. (2012), ^15^Fontein et al. (2014), ^16^Masi et al. (2001), ^17^Zhang et al. (2013), ^18^Cho et al. (2012), ^19^Shimodaira et al. (2010), ^20^Yang et al. (2013), ^21^Andrew et al. (2012), ^22^Tsuchiya et al. (2005), ^23^Huber et al. (2005), ^24^Setiawan et al. (2004), ^25^Shi et al. (2016), ^26^Jones et al. (2009), ^27^Audet-Walsh et al. (2012), ^28^Loth et al. (2014), ^29^Alshogran (2017), ^30^Qin et al. (2006), ^31^Goodarzi et al. (2006), ^32^Zarrabeitia et al. (2007), ^33^Wöhrle et al. (1990), ^34^Adjei et al. (2003), ^35^Goodarzi et al. (2007), ^36^Ashton et al. (2010), ^37^Lilla et al. (2007), ^38^Xiao et al. (2014), ^39^Sun et al. (2005), and ^40^Mueller et al. (2015)*.

*Bold text indicates the system affected, italics text indicates the name of the disease*.

More recently, there is a re-emerging interest in developing novel intracrine drugs. A number of compounds are in their clinical phases, like STS inhibitors (Maltais and Poirier, 2011; Woo et al., 2011; Purohit and Foster, 2012; Pohl et al., 2014; Pautier et al., 2017) or inhibitors of AKR1C3/17βHSD5, which are of particular interest because this enzyme has crucial role in androgen/estrogen and prostaglandin biosynthesis (Penning, 2017). Bayer's AKR1C3/17βHSD5 inhibitor BAY 1128688 has a modified estrogen core, it interferes with both pathways, and is in phase II clinical trial for endometriosis (Bothe et al., 2017). Astellas Pharma potent and selective AKR1C3/17βHSD5 inhibitor ASP-9521 had only modest effect in a phase II study on prostate cancer as single drug, but combination therapy approaches remain to be studied (Kikuchi et al., 2014; Loriot et al., 2014).

HSD inhibitors are being studied in the area of hormone-dependent diseases, with 11βHSD inhibitors being in clinical trials for metabolic disorders (Ye et al., 2017) and 17βHSD inhibitors approaching the clinical phase for a number of gynecological indications (Table 3; Abdelsamie et al., 2017).

## Intracrinology in peripheral tissues

In this paragraph, intracrinology of endometrium, GIT, bone, lungs, and CNS is reviewed. To comprehensively understand the ability of these tissues and systems to generate estrogens and other steroids, we have performed a systematic search of all original papers published in English until June 2018 that described the levels of intracrine enzymes (those indicated in Table 2-mRNA, protein or activity) in healthy tissues. In total 177 if the four extra ref are allowed papers were reviewed, and for details of this search, see Supplemental panel: “Systematic Review.” The results of this systematic review are summarized in Tables 6–**8** and are briefly overviewed in each section dedicated to the distinct tissues or systems. Reports describing the enzymes in cultured cells or cell lines were excluded (may have been discussed elsewhere, though). Each section follows then with a non-systematic overview of the role of intracrinology in pathophysiology. A brief non-systematic description of the intracrinology of the skin, immune system and adipose tissue is also given. We will not describe the intracrinology of breast, prostate and liver (where steroid catabolism is the most relevant aspect), and we redirect the reader to recent reviews (Foster et al., 2008a; Luu-The et al., 2008; Luu-The and Labrie, 2010; Labrie and Labrie, 2013; Labrie, 2015; Mueller et al., 2015; Zhao et al., 2016; Hilborn et al., 2017; Penning, 2017).

**Table 6:** Expression of intracrine enzymes in endometrium–results of the systematic search.

**Name**			**Menopausal status**		
		**Technique****^#^**	**Pre**	**Post**	**References**
StAR	mRNA	RT-PCR	yes	yes	Bukulmez et al., 2008a; Attar et al., 2009; Sinreih et al., 2017b^&^
CYP11A1	mRNA	RT-PCR	yes	n.d.	Tsai et al., 2001; Rhee et al., 2003; Attar et al., 2009; Sinreih et al., 2013; Huhtinen et al., 2014
		RT-PCR	no	n.d.	Rhee et al., 2003
CYP17A1	mRNA	RT-PCR	yes	n.d.	Tsai et al., 2001; Attar et al., 2009; Huhtinen et al., 2014
		RT-PCR	no	n.d.	Rhee et al., 2003
		IHC	no	no	Watanabe et al., 1995
CYP19A1	mRNA	RT-PCR	yes	yes	Dheenadayalu et al., 2002; Brosens et al., 2004; Matsuzaki et al., 2006; Pathirage et al., 2006; Smuc et al., 2006, 2009; Dassen et al., 2007; Bukulmez et al., 2008b; Attar et al., 2009; Smuc and Rizner, 2009; Lépine et al., 2010; Cornel et al., 2012; Huhtinen et al., 2012a; Delvoux et al., 2014; Sinreih et al., 2017a
		RT-PCR	no	no	Bulun et al., 1993, 1994; Watanabe et al., 1995; Noble et al., 1996, 1997; Kitawaki et al., 1999; Bacallao et al., 2008; Colette et al., 2009
		ISH	no	n.d.	Watanabe et al., 1995
	Protein	IHC	yes	yes	Maentausta et al., 1990; Kitaoka et al., 2004; Maia et al., 2006, 2007; Hudelist et al., 2007; Vouk et al., 2011; Miller et al., 2012a
		IHC	no	no	Watanabe et al., 1995; Kitawaki et al., 1999; Velasco et al., 2006; Acién et al., 2007; Jeon et al., 2007; Bukulmez et al., 2008b; Colette et al., 2009
		WB	N.d.	yes	Knapp et al., 2013
		activity	no	no	Bulun et al., 1993; Watanabe et al., 1995; Noble et al., 1997
		activity	yes	yes	Tseng et al., 1982; Yamaki et al., 1985; Taga et al., 1990; Yamamoto et al., 1990a,b, 1993a,b; Jongen et al., 2005; Purohit et al., 2008
3βHSD1	mRNA	RT-PCR	yes	yes	Rhee et al., 2003; Vani et al., 2007; Attar et al., 2009; Smuc et al., 2009; Gibson et al., 2013; Sinreih et al., 2013
	Protein^*^	IHC	yes	n.d.	Rhee et al., 2003; Vani et al., 2007
		IHC	no	no	Watanabe et al., 1995
3βHSD2	mRNA	RT-PCR	yes	yes	Tsai et al., 2001; Attar et al., 2009; Huhtinen et al., 2014; Osinski et al., 2018
17βHSDs					
oxidative activity			yes	yes	Tseng and Gurpide, 1974; Pollow et al., 1975a,b, 1976; Polow et al., 1975; Tseng et al., 1977; Lane, 1990; Kitawaki et al., 2000; Utsunomiya et al., 2001; Delvoux et al., 2007, 2009; Cornel et al., 2012
reductive activity			yes	yes	Maentausta et al., 1990; Delvoux et al., 2007, 2009, 2014; Bacallao et al., 2008
			no	no	Utsunomiya et al., 2001
17βHSD1	mRNA	RT-PCR	yes	yes	Zeitoun et al., 1998; Dassen et al., 2007; Smuc et al., 2007, 2009; Bacallao et al., 2008; Huhtinen et al., 2012a; Colette et al., 2013; Delvoux et al., 2014; Sinreih et al., 2017a; Osinski et al., 2018
		NB	yes	n.d.	Zeitoun et al., 1998
		RT-PCR	no	no	Casey et al., 1994; Utsunomiya et al., 2001
	Protein	IHC	yes	yes	Maentausta et al., 1990^&^; Mäentausta et al., 1991; Li et al., 2003; Dassen et al., 2007; Colette et al., 2013; Mori et al., 2015; He et al., 2016; Sinreih et al., 2017a
		IHC	no	no	Utsunomiya et al., 2001
17βHSD2	mRNA	RT-PCR	yes	yes	Mäentausta et al., 1991; Zeitoun et al., 1998; Kitawaki et al., 2000, 2002; Utsunomiya et al., 2001; Matsuzaki et al., 2006; Smuc et al., 2006, 2007, 2009; Carneiro et al., 2007; Dassen et al., 2007; Vani et al., 2007; Bacallao et al., 2008; Hevir et al., 2011b; Huhtinen et al., 2012a; Colette et al., 2013; Delvoux et al., 2014; Sinreih et al., 2017a; Osinski et al., 2018
		NB	yes	n.d.	Zeitoun et al., 1998
	Protein	IHC	yes	yes	Scublinsky et al., 1976; Ciuffi et al., 1982; Utsunomiya et al., 2001; Dassen et al., 2007; Colette et al., 2013; Cornel et al., 2017; Sinreih et al., 2017a
17βHSD4	mRNA	RT-PCR	yes	yes	Dassen et al., 2007; Smuc et al., 2009; Huhtinen et al., 2012a; Delvoux et al., 2014
		NB	yes	n.d.	Möller et al., 1999
17βHSD6	mRNA	RT-PCR	yes	n.d.	Huang and Luu-The, 2000; Huhtinen et al., 2012a
17βHSD7	mRNA	RT-PCR	yes	yes	Smuc et al., 2007, 2009; Smuc and Rizner, 2009; Lépine et al., 2010; Cornel et al., 2012; Huhtinen et al., 2012a; Delvoux et al., 2014
17βHSD8	mRNA	RT-PCR	yes	yes	Smuc and Rizner, 2009; Smuc et al., 2009
17βHSD10	mRNA	RT-PCR	yes	n.d.	Huhtinen et al., 2012a
17βHSD12	mRNA	RT-PCR	yes	yes	Smuc and Rizner, 2009; Smuc et al., 2009; Lépine et al., 2010; Cornel et al., 2012; Huhtinen et al., 2012a; Delvoux et al., 2014
17βHSD14	mRNA	RT-PCR	yes	n.d.	Huhtinen et al., 2012a; Sinreih et al., 2017a
AKR1Cs					
AKR1C1	mRNA	RT-PCR	yes	yes	Rizner et al., 2006; Smuc and Rizner, 2009; Smuc et al., 2009; Hevir et al., 2011b; Sinreih et al., 2013
AKR1C2	mRNA	RT-PCR	yes	yes	Hevir et al., 2011b; Sinreih et al., 2013
AKR1C3/17 βHSD5	mRNA	RT-PCR	yes	yes	Penning et al., 2000; Rizner et al., 2006; Vani et al., 2007; Smuc and Rizner, 2009; Smuc et al., 2009; Hevir et al., 2011b; Cornel et al., 2012; Huhtinen et al., 2012a; Sinreih et al., 2013; Delvoux et al., 2014
	Protein	IHC	yes	yes	Pelletier et al., 1999; Ito et al., 2006; Vani et al., 2007; Smuc and Rizner, 2009; Zakharov et al., 2010
SRD5As					
SRD5A1	mRNA	RT-PCR	yes	yes	Carneiro et al., 2008; Hevir et al., 2011b; Sinreih et al., 2013; Huhtinen et al., 2014
	Protein	IHC	yes	yes	Ito et al., 2002; Carneiro et al., 2008; Tanaka et al., 2015
SRD5A2	mRNA	RT-PCR	yes	yes	Carneiro et al., 2008; Hevir et al., 2011b; Sinreih et al., 2013; Huhtinen et al., 2014
	Protein	IHC	yes	yes	Ito et al., 2002; Carneiro et al., 2008; Tanaka et al., 2015
SRD5A3		RT-PCR	yes	n.d.	Huhtinen et al., 2014
Sulphatase pathway			
STS	mRNA	RT-PCR	yes	yes	Tanaka et al., 2003; Utsunomiya et al., 2004; Smuc et al., 2006, 2007, 2009; Dalla Valle et al., 2007; Dassen et al., 2007; Bacallao et al., 2008; Smuc and Rizner, 2009; Lépine et al., 2010; Colette et al., 2013; Huhtinen et al., 2014; Piccinato et al., 2016b; Sinreih et al., 2017a
		RT-PCR	no	no	Miki et al., 2002
		Comp-RT	yes	n.d.	Yanaihara et al., 2001
	Protein	IHC	yes	yes	Yanaihara et al., 2001; Utsunomiya et al., 2004; Dassen et al., 2007; Cornel et al., 2017; Sinreih et al., 2017a
		IHC	no	no	Miki et al., 2002
		activity	yes	yes	Warren and French, 1965; Prost and Adessi, 1983; Adessi et al., 1984; Platia et al., 1984; Yamamoto et al., 1990a, 1993a; Tanaka et al., 2003; Bacallao et al., 2008; Purohit et al., 2008; Delvoux et al., 2009
		IHC	no	no	Utsunomiya et al., 2004
SULT1E1	mRNA	RT-PCR	yes	yes	Yamamoto et al., 1993a; Miki et al., 2002; Tanaka et al., 2003; Utsunomiya et al., 2004; Smuc et al., 2006, 2007; Dassen et al., 2007; Bacallao et al., 2008; Smuc and Rizner, 2009; Lépine et al., 2010; Hevir et al., 2011a, 2013; Colette et al., 2013; Piccinato et al., 2016b; Sinreih et al., 2017a
		NB	yes	n.d.	Rubin et al., 1999
	Protein	IHC	yes	yes	Miki et al., 2002; Utsunomiya et al., 2004; Hudelist et al., 2007; Cornel et al., 2017; Sinreih et al., 2017a
		activity	yes	yes	Tanaka et al., 2003; Utsunomiya et al., 2004; Bacallao et al., 2008; Purohit et al., 2008
SULT1A1	mRNA	RT-PCR	yes	yes	Hevir et al., 2011a, 2013
SULT1A1		NB	yes	n.d.	Rubin et al., 1999^***^
SULT2A1		NB	no	n.d.	Rubin et al., 1999
SULT2B1	mRNA	RT-PCR	yes	yes	Hevir et al., 2011a, 2013

Primary/original references were analyzed and reviews were excluded (and are cited ad hoc in the text). The table report only the enzymes whose expression was assessed in reviewed studies^**^.

# *Technique abbreviations. For mRNA detection, NB: northern blot; ISH: in situ hybridisation; RT-PCR: reverse transcription semi or quantitative PCR; Comp-RT: competitive RT-PCR. For protein detection: IHC: immunohistochemistry; WB: western blotting. For enzyme activity measurement: activity*.

* *Most commercially available antibodies do not distinguish between 3βHSD1 and 3βHSD1*.

** *No publication was found describing the expression of 17βHSD9, 11, 13, 15, DHRS11 and AKR1C4*.

*** *The same study also detected expression of SULT1A3 (Rubin et al., 1999)*.

n.d.: not determined.

& *Protein level was measured by radioimmunoassay*.

### Endometrium

The actions of steroid hormones in the endometrium are mediated by hormone-receptors *via* the classical mechanisms, although non-genomic and rapid signaling are also present (Groothuis et al., 2007; Zwart et al., 2011; Flach and Zwart, 2016; Hewitt et al., 2016). Estrogens and P control the menstrual cycle (Groothuis et al., 2007; Andersen and Ezcurra, 2014) and the endometrium during the window of implantation (WOI), occurring in the mid-luteal phase (Wang and Dey, 2006).

In rats, the WOI is characterized by high E2 plasma levels, and endometrial ERα and PR expression shows specific and varying cytosolic/nuclear patterns (Singh et al., 1996). ERα and PR expression decreases after ovulation and in preimplantation stages in both mice (Vasquez and DeMayo, 2013) and primates (*Macaca mulatta*) (Ghosh et al., 1999).

Rodent genetic models unraveled some molecular mechanisms underlying the estrogen-dependency of these processes. ERα-KO mice are infertile, no implantation occurs, endometrium is hypoplastic and estrogen response is absent (Couse and Korach, 1999; Walker and Korach, 2004). Not only its absence, but also sustained estrogen signaling has deleterious effects on endometrial receptivity, as recapitulated by mice with uterine COUP-TFII ablation. These mice exhibit increased estrogen signaling and asynchrony between embryo competency and uterine receptivity with consequent implantation defects. This effect is rescued by treatment with the antiestrogen ICI-182780 (Lee et al., 2010). Additionally, the duration of E2 exposure and its dosage affect endometrial receptivity and WOI length in mice (Ma et al., 2003).

Available human data, mostly obtained in the context of assisted reproduction technologies (ART), also indicate that steroid stimulation retards or shortens the luteal phase, the WOI, causes shifts in the appearance of pinopodes (a classical WOI marker) and causes asynchrony between ovarian and menstrual cycles (Devroey et al., 2004).

#### Intracrinology in healthy endometrium–systematic search

Initial studies on steroid hormone metabolism in the endometrium date back to 1965 with first demonstration of the STS activity, followed by investigation on the oxidative and reductive 17βHSD activities (Table 6).

Both pre and postmenopausal tissues possess oxidative and reductive 17βHSD activities and the expression of 17βHSD1, 2, 4, 6, 7, 8, 10, 12, 14, and AKR1C3/17βHSD5 was detected at the mRNA or protein levels. Sulphatase pathway (STS and SULT1E1; recently reviewed by Rižner, 2016), CYP19A1, 3βHSDs, SRD5As and AKR1Cs are also present, indicating that human endometrium can metabolize sulphated-compounds and DHEA to form androgens and estrogens.

Few 17βHSDs have been characterized by IHC. The low expression of 17βHSD1 poses sensitivity problems using standard detection methods (Cornel et al., 2017), and few authors reported endometrial absence of 17βHSD1 (Table 6). Type 1 17βHSD localizes in the cytoplasm of epithelial cells (Dassen et al., 2007; Colette et al., 2013; Mori et al., 2015; Sinreih et al., 2017a) and it is also detected in primary stroma cells cultured *in vitro* (Aghajanova et al., 2009; Mori et al., 2015). Type 2 17βHSD, AKR1C3/17βHSD5 and 3βHSD1 give strong reactivity in the glandular epithelium (Rhee et al., 2003; Ito et al., 2006; Dassen et al., 2007; Vani et al., 2007; Smuc and Rizner, 2009; Zakharov et al., 2010; Colette et al., 2013; Mori et al., 2015; Sinreih et al., 2017a).

CYP19A1 as well has low expression and some authors detected this enzyme only in association with diseases (see below and recently reviewed by Rižner, 2013). Although CYP19A1 immunoreactivity was initially associated with stroma cells (Watanabe et al., 1995), subsequent investigations showed also glandular expression (Kitawaki et al., 1999; Hudelist et al., 2007) and laser-capture-microdissected stroma/epithelial components detected CYP19A1 mRNA in both cell types (Matsuzaki et al., 2006).

The mRNA of those enzymes converting cholesterol to DHEA (CYP11A1, CYP17A1, StAR) and (ovarian) 3βHSD2 was reported in recent studies, suggesting that the endometrium can produce steroids from cholesterol (Table 6).

#### Intratissue steroid levels

Endometrial steroid levels were recently profiled by LC-MS. E2 levels differ between tissue and serum during the menstrual cycle, being up to five-times higher in tissue than in serum during the proliferative phase and 1.5-fold higher in the luteal period (Huhtinen et al., 2012a, 2014). T levels were lower in tissue than in serum with no cyclic changes. The levels P and P5 (and their 17-hydroxy derivatives) did not vary between serum and tissue, indicating that, contrarily to estrogens, progestogen intra-tissue levels are determined by passive diffusion from the blood (Huhtinen et al., 2014).

#### Intracrinology and reproduction

Animal models show not only that intracrine enzymes are expressed in the endometrium, but also they vary the expression levels during the endometrial phases and during implantation, as shown already during the 80's in rhesus monkeys for the oxidizing 17βHSD activity (Kreitmann et al., 1979).

In rodents, STS activity measured with [3H]E1-S in 6-days pregnant rats was lower around the implantation site compared with non-implantation sites (Loza, 1995). *In situ* hybridisation signal of 17βHSD7 mRNA varied spatio-temporally throughout implantation and early gestation, being initially detected on luminal epithelium around the implantation site and absent in decidua (embryonic day, Ed5.5). At Ed8 and Ed9.5, 17βHSD7 expression increased in the decidua capsularis (the part that interacts with the trophoblast) and later (after E9) in the junctional zone of the developing placenta and in the spongiotrophoblasts (Nokelainen et al., 2000).

A brilliant study in mice showed that decidualization is dependent on local E2 produced through CYP19A1. CYP19A1 expression increased during pregnancy and decidualization was unaffected by ovariectomy. In contrast, treatment with the aromatase inhibitor (AI) letrozole impaired decidualization and decreased decidual marker expression (e.g., PRP, BMP2 and CX43) (Das et al., 2009).

In human endometrium, 17βHSD2 and SULT1E1 are induced by P as their expression peaks in the luteal phase (Rubin et al., 1999; Tseng and Mazella, 2002; Utsunomiya et al., 2004; Dassen et al., 2007; Huhtinen et al., 2012a; Colette et al., 2013; Piccinato et al., 2016b). Since both enzymes decrease intra-tissue estrogen levels, their up-regulation is one of the mechanisms of the uterine antiestrogenic effects of P. The P-dependency of 17βHSD2 and SULT1E1 was recapitulated *in vitro* using explant cultures and primary cells (Tseng and Mazella, 2002; Dassen et al., 2007; Piccinato et al., 2016b). Luteal peak expression of other SULTs (1A1 and 2B1) was also reported (Rubin et al., 1999; Koizumi et al., 2010). Some reports also suggested that STS expression increased in the luteal phase (Tanaka et al., 2003; Piccinato et al., 2016b) with a potential role during decidualization (Tseng and Mazella, 2002). Mid-luteal phase endometrium shows also peaking expression of 3βHSD1 (mRNA and protein) (Rhee et al., 2003; Vani et al., 2007).

Two studies on human ectopic pregnancies explored the endometrium around the implanted blastocyst. Expression of 3βHSD1 (mRNA and protein) was highest in decidua obtained from ectopic pregnancies (Rhee et al., 2003) and in a study on 23 tubal pregnancies, 17βHSD1 showed highest immunoreactivity at the fetal-maternal interface (Li et al., 2003), suggestive for a role of these enzymes in the nidation site.

#### Endometriosis

Endometriosis, an estrogen-dependent benign disorder affecting up to 10% of reproductive-aged women, is associated with pelvic pain, infertility, decreased life-quality and important health care/social costs (Simoens et al., 2011, 2012; De Graaff et al., 2013, 2015, 2016; Vercellini et al., 2014). Endometriosis is characterized by the growth of endometrium-like tissue outside the uterus (ectopic locations), beside the ovaries (endometrioma), as peritoneal implants, or as deep-lesions infiltrating peritoneal organs (deep endometriosis).

The expression of intracrine enzymes in endometriosis was reviewed in 2012, (Huhtinen et al., 2012b) and among other studies, 20 papers published between 1996 and 2009 specifically described the levels of intracrine enzymes in eutopic and ectopic endometrium from patients and control women. With the exclusion of one study that included over 100 patients (Colette et al., 2009), the rest included small study populations, and in most cases, the various endometriosis types (ovarian, peritoneal and deep infiltrating) were pooled together. Various techniques were used (RT-qPCR, immunohistochemistry, enzyme activity assay). Overall, no clear conclusion could be drawn from these studies. Comparing endometriosis with controls, CYP19A1 was up-regulated (six studies), unchanged (three studies) and one study found no expression of this gene. With respect to oxidative and reductive 17βHSDs, 17βHSD1 was reported up-regulated (three studies), 17βHSD2 was reported down-regulated or unchanged and two studies reported an up-regulation of 17βHSD7 and 12 in endometriosis vs. controls (Huhtinen et al., 2012b).

Subsequent investigations also continued to report inconsistent results. No change in mRNA (Delvoux et al., 2014) or increased expression of CYP19A1 in ovarian endometriosis vs. controls (Huhtinen et al., 2012a) were reported. An increased expression of CYP19A1 was also described using *in vitro* spheroids derived from endometrial stroma cells from patients compared with controls (Mori et al., 2015).

The mRNA expression of 17βHSD1 was higher in endometriosis compared with normal tissue using patient biopsies as well as spheroid cultures derived from endometrial stroma cells of patients and controls (Delvoux et al., 2014; Mori et al., 2015). One study assessing the three endometriosis types separately (60 patients in total) described that the increased 17βHSD1 level was restricted to endometrioma during the secretory phase of the menstrual cycle (Huhtinen et al., 2012a), whereas a second study on 79 patients and 41 controls, found no change in 17βHSD1 level, but described an increased 17βHSD1/2 ratio (Colette et al., 2013).

Regarding 17βHSD2, recent investigations reported both unchanged (Delvoux et al., 2014) and down-regulated mRNA in patient biopsies compared with controls (Huhtinen et al., 2012a; Colette et al., 2013). No variations were found in 17βHSD4, 5, 7 and 12 (Smuc et al., 2009; Delvoux et al., 2014) but an increased level of 17βHSD6 mRNA was detected in endometriosis compared with controls (Huhtinen et al., 2012a).

A few studies reported detectable levels of the enzymes involved in the generation of DHEA from cholesterol (StAR, CYP11A1 and CYP17A1) in endometriosis (Tsai et al., 2001; Rhee et al., 2003; Bukulmez et al., 2008a; Attar et al., 2009; Sinreih et al., 2013, 2017b; Huhtinen et al., 2014), suggesting that, in contrast to eutopic endometrium, endometriosis is able to produce steroids from cholesterol. However, it has also been argued that the presence of paracrine confounders of ovarian origin in studies using endometriomas could bias the results (Noël et al., 2011).

The contribution of STS, SULT1E1 and other SULTs was investigated by numerous studies and also in this case, conclusions are unclear (recently reviewed, Rižner, 2016). A recent investigation using 78 specimens described increased STS levels in endometriosis vs. control samples and found that the overall balance between STS and SULT1E1 differed between eutopic and ectopic tissue, implying an unbalanced flux of sulpho-conjugated estrogens in this disease (Piccinato et al., 2016b). The same research group also described an aberrant regulation of the enzymes involved in the estrogen oxidative metabolism in endometriosis (Piccinato et al., 2016a).

Although the level of the single enzymes in the intracrine machinery varies with apparently no clear association with the disease condition, the intracrinological nature of endometriosis was recently proven by comparison between serum and tissue levels of steroids in 60 patients (eutopic and ectopic endometrium) and 16 controls. Although E2 changed cyclically in eutopic tissue, E2 levels remained constant in the lesions and inversely correlated with the mRNA level of 17βHSD2 and 17βHSD6 suggesting an impairment in E2 deactivation to E1. P levels were equal in serum and control tissues, but resulted higher in patients and correlated with high 3βHSD2 mRNA. T, low in the tissue of controls, was over 13-times more concentrated at ectopic locations and correlated with low expression of SRD5A3 (Huhtinen et al., 2012a, 2014).

#### Endometrial cancer (EC)

EC is the most common gynecological malignancy in western society and 80% of all cases are estrogen-driven (Amant et al., 2005; Morice et al., 2015). Major serum steroids are increased in patients with EC, including several substrates for intracrine E2 synthesis (Lépine et al., 2010; Audet-Walsh et al., 2011). In addition, tissue-steroid levels differ between cancer, normal tissue and serum and correlate with the levels of specific intracrine enzymes (see below) (Tanaka et al., 2015).

A systematic review recently explored all studies published between 1990 and 2017 assessing the expression of 17βHSD1, 2, STS, SULT1E1, and CYP19A1, with results that describe unbalanced intracrine regulation and important inter-patient variability (Cornel et al., 2018). Most studies compared cases with controls or tumor tissue with adjacent normal endometrium. Compared with normal tissue (from controls or adjacent to tumor), 17βHSD1 was found increased in EC (Cornel et al., 2012), decreased (Smuc and Rizner, 2009; Lépine et al., 2010) and undetected (Utsunomiya et al., 2001, 2003); 17βHSD2 was found decreased (Utsunomiya et al., 2003, 2004) or increased (Lépine et al., 2010; Cornel et al., 2012; Sinreih et al., 2013); AKR1C3/17βHSD5 was found unchanged (Cornel et al., 2012; Sinreih et al., 2013), increased (Ito et al., 2016) and decreased (Zakharov et al., 2010); 17βHSD7 both decreased (Smuc and Rizner, 2009) and unchanged (Lépine et al., 2010; Cornel et al., 2012) and 17βHSD12 was unchanged (Smuc and Rizner, 2009; Cornel et al., 2012) or increased in tumors vs. controls (Lépine et al., 2010). One recent report described decreased 17βHSD14 levels in tumor compared with adjacent tissue (Sinreih et al., 2017a). Controversial results apply to CYP19A1, described as increased (Watanabe et al., 1995; Utsunomiya et al., 2001, 2004; Smuc and Rizner, 2009) and unchanged (Jongen et al., 2005; Pathirage et al., 2006; Cornel et al., 2012). STS/SULT1E1 expression is also inconsistent in different studies (recently reviewed in Mueller et al., 2015; Rižner, 2016).

Recent studies exploring the association between enzyme levels and tumor characteristics found a correlation between STS with tumor grade and lymphovascular invasion (Sinreih et al., 2017a) and described an association between high CYP19A1 or 17βHSD1 and poor patient prognosis (Segawa et al., 2005; Cornel et al., 2017).

Other investigations emphasized the potential antiestrogenic and protective roles of androgens and P. Formation of DHT (via conversion of A4 to T by AKR1C3/17βHSD5 and of T to DHT by SRD5As) has potential antiestrogenic action because it devoids tissue from T (substrate of CYP19A1 yielding E2) and because it has direct endometrial antiproliferative effects via AR (Ito et al., 2016). Similar to the AKR1C3/17βHSD5 data reported earlier, results on SDR5A expression are inconclusive as SRD5A2 was down-regulated in a study on 47 tumor specimens compared with adjacent normal tissue (Sinreih et al., 2013), but both SRD5A1 and SRD5A2 resulted unchanged in another study on 122 tumors (although only five controls were studied) (Tanaka et al., 2015). This last study found however increased androgen levels (T and DHT) in tissue vs. blood. High DHT levels were restricted to samples with high SRD5A1 immunohistochemical staining. In addition, AR and SRD5A1 positivity was associated with good patient prognosis (Tanaka et al., 2015). The prognostic value of AR is confirmed by independent investigations (Tangen et al., 2016).

P is well-known for its antiestrogenic action, PR positivity is a good prognostic marker (Tangen et al., 2014) and P synthesis and metabolism are disturbed in EC (Sinreih et al., 2013). Interestingly, in a study on 47 tumors and adjacent normal tissues, EC had decreased StAR and CYP11A1 mRNA levels, indicative of diminished *de novo* steroid synthesis (Sinreih et al., 2013, 2017b). At the same time, EC showed decreased SRD5A2 and increased 17βHSD2 indicative of a diminished rate of conversion of P to 5αDHP and of 20αDHP to 5α-pregnan-20-ol-3-one, but increased conversion of 20αDHP to P (see Figure 2).

#### Other endometrial/gynecological disorders

Although literature is scarce, a potential role of intracrinology is postulated for ovarian cancer (Ito et al., 2016), for adenomyosis and fibroids (Rižner, 2016), for sarcoma, where CYP19A1 expression may have prognostic significance (Kitaoka et al., 2004) and among infertile women (Brosens et al., 2004).

#### Intracrine drug targets

Endometriosis: blocking the systemic estrogen signaling via P, or GnRH agonist is standard care (Vercellini et al., 2014). Blocking the intracrine E2 generation is the future approach with on-going preclinical/clinical research.

STS inhibition showed promising results. Irosustat (Table 3) inhibited up to 100% the formation of free steroids using *ex-vivo* material from 27 patients (Purohit et al., 2008) and STS inhibition showed good results in a mouse model of endometriosis, where decreased size and weight of the lesions was observed (Colette et al., 2011). A phase-I clinical trial on 24 volunteers proved the safety of the STS inhibitor E2MATE (PLG2001), which reduced STS activity by over 90% and induced changes in endometrial markers (both alone or co-administered with norethindrone acetate) (Pohl et al., 2014).

Inhibitors of 17βHSD1 are in preclinical phase, and promising results are described using a primate model of endometriosis, where decreased behavior/pain symptoms were reported (Arnold and Einspanier, 2013) and using *ex-vivo* material from endometriosis patient (over 70% of the patients showed over 80% of enzyme inhibition) (Delvoux et al., 2014).

AKR1C3/17βHSD5 inhibition can interfere with E2, androgen synthesis, and reduce prostaglandin-associated inflammation/proliferation and an inhibitor has recently entered a phase II trial for endometriosis (Table 3). Overall, AIs have limited efficacy for endometriosis (Ferrero et al., 2011; Dunselman et al., 2014),

EC: only in case of advanced stage/metastatic disease hormonal care is given (progestogen, tamoxifen or AIs). AIs alone have limited efficacy with low response rates (Rose et al., 2000; Ma et al., 2004; Lindemann et al., 2014). Promising data were obtained using dual regimen (AI and mTOR inhibitor; Slomovitz et al., 2015) and additional trials on combinatory regimen are on-going. STS inhibitors showed promising results in a mouse subcutaneous model of EC, with decreased tumor growth by 48–67% (Foster et al., 2008b). However, a phase II trial on advanced stage EC was stopped because of the absence of added benefit compared with progestogen treatment (Purohit and Foster, 2012; Pautier et al., 2017).

Preclinical studies on 17βHSD1 inhibitors showed promising results in a mouse model of endometrial hyperplasia (Saloniemi et al., 2010;Järvensivu et al., 2015) and in various models of EC (Konings et al., 2018).

#### Endometrium: conclusions

**Figure 3:**
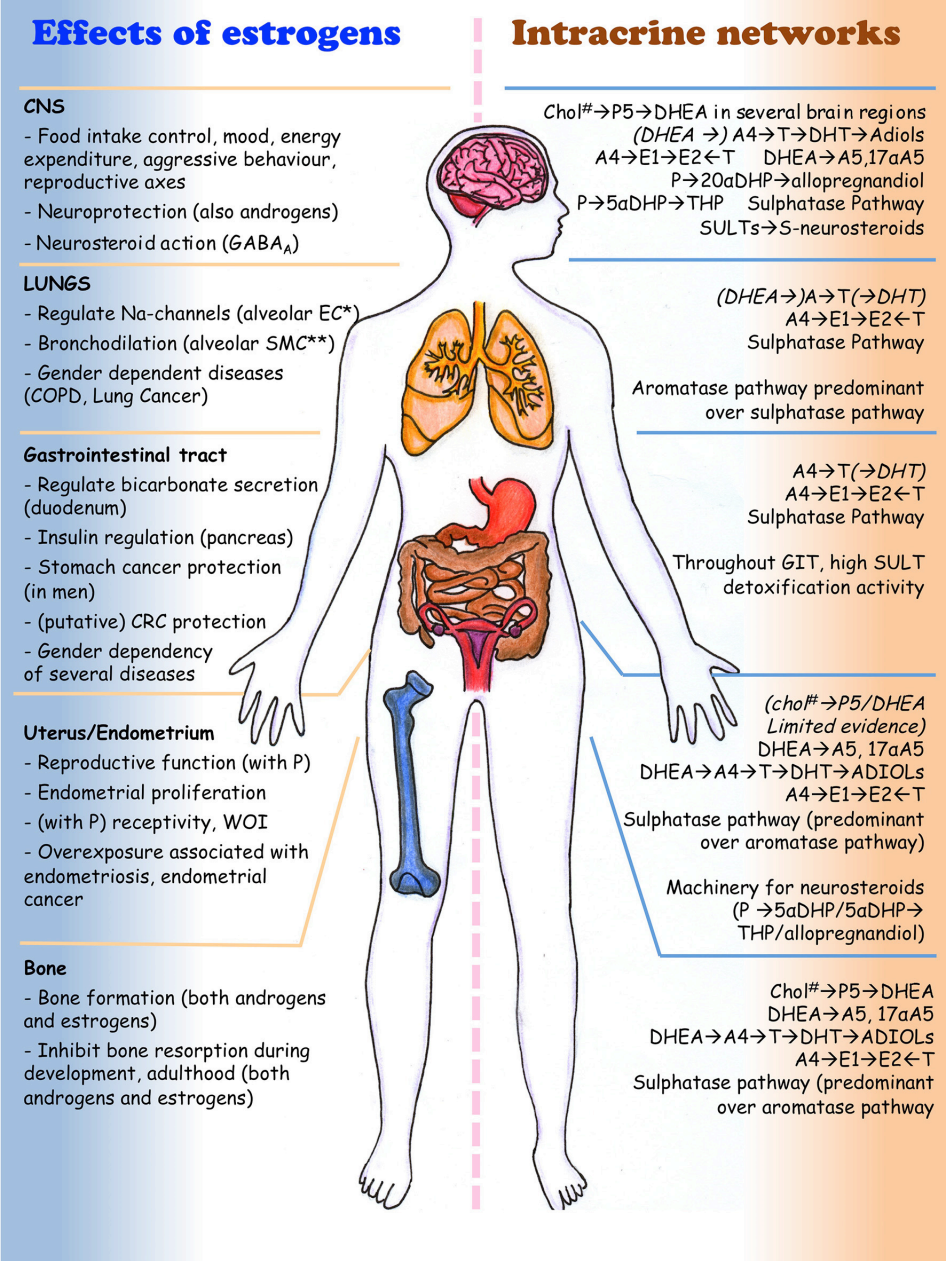
Effect of steroids (mainly estrogens) and intracrine networks in central nervous system, lungs, digestive system, uterus and bone. Italics and by brackets are those metabolism/reactions that need conformation by independent authors (because validated at the mRNA level only or in few studies). * EC, epithelial cells; **SMC, smooth muscle cells; ^#^Chol, cholesterol. The drawing was kindly generated by Dr. Margaretha A. Skowron (Department of Urology, University Düsseldorf, Germany) for this review.

The ability to synthesize DHEA from cholesterol (reported by few studies) needs confirmation. However, the endometrium possesses the enzymatic machinery to metabolize sulphated-compounds and DHEA and form androgens and estrogens, (although this contention is wrangled by other authors: Labrie and Labrie, 2013; Labrie, 2015). Further, the endometrium can metabolize androgens and progestogens via AKR1Cs and SRD5As to produce a wide range of compounds, including estrogens (Table 6 and Figure 3). The morphological changes during the menstrual cycle are accompanied by cyclic changes in intracrine steroid and enzyme levels, indicating that steroid exposure needs to be cyclically regulated to support endometrial physiology.

### Gastrointestinal tract (GIT) and digestive system (DS)

ERα and ERβ are expressed throughout the GIT and DS (esophagus, stomach, colon, gallbladder, pancreas) and epidemiological studies show important influence of sex hormones in DS physiology and disturbances, with a clear gender-dependency. In the duodenum, estrogens regulate bicarbonate secretion (Nayeb-Hashemi and Kaunitz, 2009; Tuo et al., 2011). This is an important defense mechanism of the mucosa against acids discharged from the stomach, and men develop duodenal ulcer two/three-times more often than premenopausal women (Wu et al., 2008). Such estrogen protective effect is recapitulated in animal studies exposed to estrogens and anti-estrogens, and is mediated by a rapid action (i.e., non genomic) of ERα on membrane ion channels (Smith et al., 2008).

ERα, ERβ and GPER mediate important effects on the pancreatic beta-cells during adaptation to insulin resistance periods (e.g., pregnancy, puberty, obesity; Nadal et al., 2011). In mice, ERα signaling regulates proliferation of beta-cell during development and after injury (Yuchi et al., 2015).

Men are also more likely than women to develop cancer in the esophagus, stomach and colon. Accordingly, estrogen treatment for prostate cancer decreases the incidence of gastric cancer and menopausal status in women is associated with colorectal cancer CRC risk (Freedman et al., 2007; Kennelly et al., 2008; Hogan et al., 2009; Duell et al., 2010). ERβ results oncoprotective at several GIT sites (Kennelly et al., 2008; Barzi et al., 2013; Caiazza et al., 2015) and low expression correlate with high CRC stage in mice and with poor differentiated gallbladder cancer in humans (Hogan et al., 2009).

The association between estrogens and DS cancer risk is however controversial. The Women's Health Initiative and other large studies showed that combined estrogens plus P hormone replacement therapy (HRT) decreases CRC risk, but increases that of gallbladder. In addition, CRC during HRT has a higher grade (Kennelly et al., 2008; Hogan et al., 2009; Rennert et al., 2009; Foster, 2013; Mueller et al., 2015). However, a recent randomized, placebo-controlled trial enrolling over 10,000 women receiving estrogens alone vs. placebo found no difference in CRC incidence (Lavasani et al., 2015). Such complexity is recapitulated in animal studies where estrogens and androgens can have distinct and opposite effects on colitis and CRC (Amos-Landgraf et al., 2014; Heijmans et al., 2014). Overall, the association between DS disturbances/cancers with estrogens depends on the moment in life, extent and nature (endogenous or exogenous) of exposure and is influenced by the relative balance of the receptors (Foster, 2013). Similarly, androgens influence DS pathophysiology via complex and unclear mechanisms involving classical, membrane signaling, level of free and SHBG bound T (Roshan et al., 2016).

The lack of clear conclusion and the fact that the levels of circulating endogenous estrogens in women do not influence CRC risk indicates that intracrine steroids may have a predominant role irrespective of their circulating levels (Sato et al., 2009; Falk et al., 2015).

#### Intracrinology in healthy GIT–systematic search

In total, 29 original papers were retrieved that described the levels of the intracrine enzymes in the GIT, published from the late 80's (Table 7 and Supplemental panel: “Systematic Review”).

**Table 7:** Expression of intracrine enzymes in the gastrointestinal tract (GIT)-results of the systematic search.

**Gene**	**Detection**		**Gastrointestinal tract**					
	**Molecule**	**Technique^#^**	St^*^	**References**	S.I.^*^	**References**	L.I.^*^	**References**
CYP11A1	Protein	IHC	no	Saitoh et al., 1992	n.d.		n.d.	
CYP19A1	Protein	Activity	n.d.		n.d.		yes	English et al., 2000
CYP19A1	Protein	IHC	no	Saitoh et al., 1992	n.d.		yes	English et al., 2000
		WB	no	Saitoh et al., 1992	n.d.		n.d.	
HSD17B oxidative activity			n.d.		n.d.		yes	English et al., 1999
HSD17B reductive activity			n.d.		n.d.		yes	English et al., 1999
17βHSD1	mRNA	RT-PCR	no	Oduwole et al., 2003a	n.d.		yes	Rawłuszko et al., 2011
		NB	n.d.		no	Casey et al., 1994	no	Casey et al., 1994
		ISH	n.d.		no	Oduwole et al., 2002	no	Oduwole et al., 2002
	Protein	WB	n.d.		n.d.		yes	Rawłuszko et al., 2011
17βHSD2	mRNA	RT-PCR	yes	Oduwole et al., 2003a; Frycz et al., 2015	n.d.		n.d.	
		NB	n.d.		yes	Casey et al., 1994	yes	Casey et al., 1994
		ISH	n.d.		yes	Oduwole et al., 2002	yes	Oduwole et al., 2002, 2003b
	Protein	IHC	n.d.		n.d.		yes	English et al., 2000; Mueller et al., 2015
		WB	yes	Frycz et al., 2015	n.d.		yes	English et al., 2000; Mueller et al., 2015
17βHSD4	mRNA	NB	n.d.		yes	Möller et al., 1999	yes	Möller et al., 1999
	Protein	IHC	n.d.		n.d.		yes	English et al., 2000; Mueller et al., 2015
		WB	n.d.		n.d.		yes	English et al., 2000; Mueller et al., 2015
17βHSD10	mRNA	RT-PCR	n.d.		n.d.		yes	De Preter et al., 2012
17βHSD12	mRNA	RT-PCR	n.d.		yes	Sakurai et al., 2006	n.d.	
		NB	n.d.		yes	Sakurai et al., 2006	no	Sakurai et al., 2006
	Protein	IHC	yes	Sakurai et al., 2006	yes	Sakurai et al., 2006	yes	Sakurai et al., 2006
AKR1C3	mRNA	Comp-RT	yes	Frycz et al., 2016	n.d.		n.d.	
		NB	yes	Frycz et al., 2016	yes	Lin et al., 1997	yes	Lin et al., 1997
	Protein	IHC	yes	Miller et al., 2012b; Chang et al., 2013	yes	Chang et al., 2013	yes	Chang et al., 2013
SRD5A1	Protein	IHC	yes	Aumüller et al., 1996	yes	Aumüller et al., 1996	yes	Aumüller et al., 1996
SRD5A2	Protein	IHC	yes	Aumüller et al., 1996	yes	Aumüller et al., 1996	yes	Aumüller et al., 1996
STS	mRNA	RT-PCR	n.d.		no	Miki et al., 2002	yes	Dalla Valle et al., 2007
		RT-PCR	–		–		no	Miki et al., 2002
	Protein	IHC	n.d.		no	Miki et al., 2002	no	Miki et al., 2002; Sato et al., 2009
	Protein	Activity	n.d.		n.d.		yes	Munroe and Chang, 1987
SULT1E1	mRNA	RT-PCR	no	Nishimura and Naito, 2006	yes	Miki et al., 2002; Nishimura and Naito, 2006	yes	Miki et al., 2002; Teubner et al., 2007; Riches et al., 2009
			–		–		no	Nishimura and Naito, 2006
		NB	n.d.		yes	Her et al., 1996	n.d.	
	Protein	IHC	no	Chen et al., 2003; Teubner et al., 2007	yes	Miki et al., 2002; Teubner et al., 2007	yes	Miki et al., 2002; Teubner et al., 2007; Sato et al., 2009
		WB	n.d.		yes	Her et al., 1996; Chen et al., 2003; Teubner et al., 2007; Riches et al., 2009	no	Chen et al., 2003
		Activity	no	Chen et al., 2003; Teubner et al., 2007	yes	Teubner et al., 2007	yes	Teubner et al., 2007
SULT2A1	mRNA	RT-PCR	no	Nishimura and Naito, 2006	yes	Nishimura and Naito, 2006	no	Nishimura and Naito, 2006
		ISH	yes	Tashiro et al., 2000	n.d.		n.d.	
		NB	n.d.		yes	Her et al., 1996	n.d.	
	Protein	IHC	no	Teubner et al., 2007	yes	Teubner et al., 2007	yes	Teubner et al., 2007
		WB	yes	Tashiro et al., 2000; Chen et al., 2003	yes	Her et al., 1996; Chen et al., 2003; Teubner et al., 2007; Riches et al., 2009	yes	Chen et al., 2003; Teubner et al., 2007; Riches et al., 2009
		WB	n.d.		n.d.		no	Chen et al., 2003
		Activity	yes	Tashiro et al., 2000	yes	Chen et al., 2003; Teubner et al., 2007	yes	Chen et al., 2003; Teubner et al., 2007
		Activity	no	Teubner et al., 2007	–		–	
SULT1A1	mRNA	RT-PCR	yes	Nishimura and Naito, 2006	yes	Nishimura and Naito, 2006	yes	Nishimura and Naito, 2006
	Protein	IHC	yes	Teubner et al., 2007	yes	Teubner et al., 2007	yes	Teubner et al., 2007
		WB	yes	Teubner et al., 2007	yes	Teubner et al., 2007; Riches et al., 2009	yes	Teubner et al., 2007; Riches et al., 2009
		Activity	yes	Teubner et al., 2007	yes	Teubner et al., 2007	yes	Teubner et al., 2007
SULT2B1	mRNA	RT-PCR	no	Nishimura and Naito, 2006	yes	Nishimura and Naito, 2006	no	Nishimura and Naito, 2006
		NB	n.d.		n.d.		no	Meloche and Falany, 2001

Primary/original references were analyzed and reviews were excluded (and are cited ad hoc in the text). The table report only the enzymes whose expression was assessed in reviewed studies^**^.

* *St: stomach; S.I.: small intestine; L.I.: large intestine*.

# *Technique abbreviations. For mRNA detection, NB: northern blot; ISH: in situ hybridisation; RT-PCR: reverse transcription quantitative (or semi-quantitative) PCR; Comp-RT: competitive RT-PCR assay; NB: northern blotting. For protein detection: IHC: immunohistochemistry; WB; western blotting; activity: enzyme activity measurement*.

^**^No publication was found describing the expression of StAR, 3βHSD1, 3βHSD2, 17βHSD6, 7, 8, 9, 11, 13, 14 15, DHRS11, AKR1C1 AKR1C2 and AKR1C4.

*n.d.: not determined*.

##### Stomach intracrinology.

The stomach is an endocrine tissue, and in rodents it produces steroids starting at birth and throughout adulthood (Kobayashi et al., 2013). Human gastric mucosa expresses 17βHSD1, 2, 12 and AKR1C3/17βHSD5 (Table 7). The mRNA for 17βHSD2 in mucosa surface and glandular epithelium inversely correlates with age in both genders (Oduwole et al., 2003a). Luminal gastric mucosa has strong AKR1C3/17βHSD5 immunoreactivity that decreases toward the gastric pits (Chang et al., 2013). Weak immunoreactivity for 17βHSD12 localizes in the fundic glands and in the squamous epithelium of the esophagus (Sakurai et al., 2006).

Sulphatases in parietal cells of the gastric glands have a protective role in detoxification. Estrogenic SULT1E1 is not expressed whereas data for SULT2A1 are inconsistent. SULT2A1 was detected in the gastric mucosa in a study on seven subjects (Tashiro et al., 2000), but it was low/absent in other studies on 39 (Teubner et al., 2007) and 23 subjects (Chen et al., 2003).

##### Small intestine: duodenum–jejunum–ileum.

Due to its high exposure to food components and harmful xenobiotics, the duodenum expresses several phase I/II enzymes including DHEA/estrogenic SULT1E1, 2A1, 1A1 (Table 7). Protein and enzyme activity of SULT1E1 and 2A1 are present in human jejunum and ileum but absent in duodenum (Teubner et al., 2007), mRNA and protein levels vary with no relation either with age or gender (Her et al., 1996; Nishimura and Naito, 2006). In a study on 23 subjects, SULT1E1 and 2A1 varied inter-individually and between different intestine tracts (Chen et al., 2003). The duodenal mucosa expresses 17βHSD2, but not 17βHSD1 (Casey et al., 1994; Oduwole et al., 2003a) and shows strong luminal AKR1C3/17βHSD5 (Chang et al., 2013) and weak 17βHSD12 immunoreactivity (Sakurai et al., 2006) that decreases toward the Brunner's gland (Chang et al., 2013).

##### Large intestine: colon, cecum, rectum

The intracrinology of healthy colon mucosa and its relation to CRC was recently reviewed (Foster, 2013). Studies dating from 1987 demonstrated the presence of CYP19A1, 17βHSD reductive and oxidative enzymatic activities, plus the expression of 17βHSD1, 2, 4, CYP19A1, STS and SULT1E1 (Table 7). Most 17βHSDs tend to have higher levels at the surface than in cryptal epithelial cells as indicated for 17βHSD2 mRNA (Oduwole et al., 2002; Foster, 2013), and for the immunoreactivity of AKR1C3/17βHSD5 (very strong; Chang et al., 2013) and 17βHSD12 (weak; Sakurai et al., 2006).

##### Pancreas

Radiolabelled substrates demonstrated the presence of CYP19A1 and SRD5A activities in human pancreatic tissue (Iqbal et al., 1983), which expresses 17βHSD2, 12, STS, SULT1E1 (Casey et al., 1994; Miki et al., 2002; Sakurai et al., 2006; Dalla Valle et al., 2007). High levels of AKR1C3/17βHSD5 localized in pancreatic ductules (acini and islets of Langerhans resulted negative; Chang et al., 2013).

#### Association with diseases

SNPs in genes controlling estrogen synthesis, response and deactivation are associated with GIT cancers (Freedman et al., 2009; Cho et al., 2012; Zeng et al., 2016) and AKR1C4 is a candidate gene in hereditary CRC (Gylfe et al., 2013; Table 5). Also variations in the expression of these genes associate with GIT disturbances. Low 17βHSD10 levels are associated with aberrant butyrate β-oxidation and ulcerative colitis (De Preter et al., 2012). The epithelial 17βHSD2 level is low in case of stomach, duodenal cancer and chronic gastritis, though it is high in regenerating epithelium close to active gastritis and ulcers (Oduwole et al., 2003a). In a study on 34 gastric tumors and adjacent healthy tissue, the mRNA and protein levels of 17βHSD2 and AKR1C3/17βHSD5 were down-regulated in cancer (Frycz et al., 2015, 2016). Some studies showed lower oxidative 17βHSD activity and mRNA level of 17βHSD2 (and 4) in CRC vs. adjacent normal tissue, suggesting a protective role of estrogen deactivation. However, another study on 35 women and 39 men found that high 17βHSD2 levels were associated with poor prognosis in female patients with distal CRC (reviewed in Foster, 2013). Also 17βHSD1 level measured by RT-qPCR and western blotting in specimens from 52 patients was lower in CRC than adjacent normal mucosa (Rawłuszko et al., 2011). CRC show also higher CYP19A1 mRNA compared with adjacent normal mucosa (*n* = 31) (Sato et al., 2012).

Although no clear target for drugs has been identified in the intracrine network, intracrine enzymes showed some values as biomarkers. In CRC, high STS/SULT1E1 ratio correlates with poor prognosis (Foster, 2013) and AKR1C3/17βHSD5 expression with lymph-node metastasis (Nakarai et al., 2015). In addition, AKR1C1 and AKR1C3/17βHSD5 associate with cisplatin resistance in CRC, hence inhibitors of these AKR1Cs may be used to re-sensitize patients to chemotherapy (Matsunaga et al., 2013). In a study were the levels of E1, E2 and DHEA-S were measured in CRC specimens and adjacent normal mucosa of men and women by LC-MS, intra-tumor estrogens were elevated and (in particular E1) correlated with poor prognosis. In line with an unfavorable role of intra-tissue estrogens, absence on STS was associated with long survival (Sato et al., 2009).

#### GIT: conclusions

Human GIT/DS is unable to metabolize cholesterol and there is no clear evidence that it expresses 3βHSDs, hence DHEA cannot be used to generate androgens and estrogens (Table 7 and Figure 3). Several SULTs are expressed throughout GIT and involved in detoxification and STS is regulated by estrogen *in vitro* via non-classical GPER signaling (Gilligan et al., 2017).

The role of steroids in pathology is complex, with divergent effects that depend on time, length and extent of exposure. In line with this, intracrine networks have unclear roles in pathogenesis. In the GIT these networks are strongly involved in the metabolisms of fatty acids and bile acids (outside the scope of this review).

### Bone tissue and skeletal system

Bones consist of mineralized connective tissue with structural and supportive functions. The hard exterior part (cortical bone) and the trabecular and spongy cancellous tissue filling the bone interior are identical but differ in the level of mineralization. Osteoblasts, derived from multipotent mesenchymal stem cells, build the bone tissue through deposition of Type-I collagen and through the release of ions that combine chemically forming the bone mineral. Osteoclasts differentiate from hematopoietic stem cells and cause resorption of the mineralized bone mass. The balance between osteoblasts and osteoclasts regulates mineral deposition and resorption. Sex steroid hormones contribute to control bone development during puberty, contribute to bone physiology, bone mass maintenance and regulate the rate of mineral bone deposition and resorption (Svoboda et al., 2010).

The presence of the ERs as well as other hormone-receptors in normal osteoblastic cells, osteoclasts and osteoblasts is documented (Gruber et al., 2002) and estrogens and androgens stimulate bone formation and inhibit bone resorption in both males and females. During human puberty and throughout adulthood, E2 and T induce osteoblast proliferation (Kassem et al., 1998), which is mediated by IGF and GH (Riggs et al., 2002; Svoboda et al., 2010). Such human effects are well recapitulated in animal models. ERα-KO (Vidal et al., 2000) and CYP19A1-KO mice (Oz et al., 2000) exhibit low BMD in both genders and E2 treatment rescues the CYP19A1-KO phenotype (Miyaura et al., 2001). Additionally, ovariectomy stimulates osteoclast differentiation through (indirect) increased levels of IL-1, 6 and TNF in osteoblasts and other bone-derived stromal cells (Gruber et al., 2002; Svoboda et al., 2010).

Accelerated bone loss and increased osteoporotic fractures are associated with postmenopausal estrogen deficiency and low sex steroid levels elicit similar manifestations in men (Compston, 2001; Riggs et al., 2002; Syed and Khosla, 2005). Free E2 levels are associated with low lumbar spine and femoral neck bone mineral density (BMD) in both genders (Zarrabeitia et al., 2007) and estrogen therapy reduces bone loss and the risk of fracture in women with osteoporosis (Gruber et al., 2002).

#### Intracrinology in healthy bone–systematic search

Bone expresses CYP19A1 and 17βHSD1, and mRNA *in situ* hybridisation and immunohistochemistry signals were seen in lining cells, osteoblasts, chondrocytes of articular cartilage, and adipocytes adjacent to bone trabeculae in both male and female tibiae. CYP19A1 mRNA was also widely present in various bones (ribs, femurs) with inter-individual variability, but no relation with gender or age (Sasano et al., 1997). STS and 17βHSD activities were demonstrated by recovery of [3H]E1 and [3H]E2 after incubating femur-head fragments with [3H]E1-S (15 women and 12 men with osteoarthritis indicated for hip replacement). No gender-related differences were observed and E2 formation from androgens was lower than that from E1-S, indicating a predominant role of the sulphatase pathway in bone estrogen supply (Muir et al., 2004). Subsequent studies also demonstrated the presence of CYP11A1, CYP17A1, 17βHSD reductive and oxidative activity in bone tissues (Table 8). Overall, however, only six papers describing the level of intracrine enzymes in bone tissues were retrieved by the systemic search (Table 8) and most studies on bone intracrinology used *in vitro* cell cultures. *In vitro* studies were not included in our systematic review, but those on bone are briefly described in the next paragraph. These studies demonstrate the presence of a complex intracrine networks.

**Table 8:** Expression of intracrine enzymes in bone, lungs and central nervous system (CNS) – results of the systematic search.

**Gene**	**Detection**		**Bone**	**References**	**Lung**	**References**	**CNS**	**References**
	**Molecule**	**Technique^#^**						
StAR	mRNA	RT-PCR	n.d.		yes^∧^	Pezzi et al., 2003	n.d.	
CYP11A1	mRNA	RT-PCR	yes	Rodríguez-Sanz et al., 2015	yes^∧^	Pezzi et al., 2003	yes	Stoffel-Wagner, 2001
		Comp-RT	n.d.		n.d.		yes	Beyenburg et al., 1999; Watzka et al., 1999
	Protein	WB	yes^*^	Rodríguez-Sanz et al., 2015			n.d.	
CYP17A1	mRNA	RT-PCR	yes	Rodríguez-Sanz et al., 2015	no^∧^	Pezzi et al., 2003	yes	Stoffel-Wagner, 2001
		RT-PCR	–		–		no	Steckelbroeck et al., 2004b
	Protein	WB	yes^*^	Rodríguez-Sanz et al., 2015	n.d.		n.d.	
		IHC	n.d.		n.d.		no	Steckelbroeck et al., 2004b
		Activity	n.d.		n.d.		no	Steckelbroeck et al., 2004b
CYP19A1	mRNA	RT-PCR	yes	Oz et al., 2001	yes	Pezzi et al., 2003; Aresti et al., 2014; Kohno et al., 2014; Konings et al., 2017	yes	Sasano et al., 1998; Stoffel-Wagner et al., 1998a; Stoffel-Wagner, 2001; Yague et al., 2006
		Comp-RT	n.d.		n.d.		yes	Stoffel-Wagner et al., 1999a
		ISH	yes	Sasano et al., 1997	n.d.		n.d.	
	Protein	IHC	yes	Sasano et al., 1997; Oz et al., 2001	yes	Verma et al., 2013; Siegfried and Stabile, 2014; Taniuchi et al., 2014; Konings et al., 2017	yes	Naftolin et al., 1996; Yague et al., 2006, 2010
		ELISA	n.d.		yes	Aresti et al., 2014; Skjefstad et al., 2016; Tanaka et al., 2016	n.d.	
		Activity	yes	Schweikert et al., 1995	yes	Taniuchi et al., 2014	yes	Naftolin and MacLusky, 1982; Stoffel-Wagner, 2001
3βHSD1	mRNA	RT-PCR	n.d.		yes^∧^	Pezzi et al., 2003	no	Stoffel-Wagner, 2001
3βHSD2	mRNA	RT-PCR	n.d.		no^∧^	Pezzi et al., 2003	no	Stoffel-Wagner, 2001
17βHSDs								
HSD17B oxidative activity			yes	Muir et al., 2004	n.d.		yes	Steckelbroeck et al., 1999, 2003; Stoffel-Wagner, 2001
HSD17B reductive activity			yes	Muir et al., 2004	n.d.	Tsai et al., 2001; Attar et al., 2009; Huhtinen et al., 2014	yes	Steckelbroeck et al., 1999, 2003; Stoffel-Wagner, 2001
17βHSD1	mRNA	RT-PCR	n.d.		yes	Takeyama et al., 2000; Drzewiecka et al., 2015; Konings et al., 2017)	yes	Stoffel-Wagner et al., 1999a; Stoffel-Wagner, 2001
		Comp-RT	n.d.		n.d.		yes	Beyenburg et al., 2000
	mRNA	ISH	yes	Sasano et al., 1997	n.d.		n.d.	
17βHSD1	Protein	Comp-RT	n.d.		n.d.		yes	Stoffel-Wagner et al., 1999b; Beyenburg et al., 2000
		IHC	yes	Sasano et al., 1997	yes	Verma et al., 2013; Drzewiecka et al., 2015; Konings et al., 2017	n.d.	
		WB	n.d.		yes	Drzewiecka et al., 2015	n.d.	
17βHSD2	mRNA	RT-PCR	n.d.		yes	Takeyama et al., 2000; Simard et al., 2010; Konings et al., 2017	no	Stoffel-Wagner, 2001
		Comp-RT	n.d.		n.d.		n.d.	Stoffel-Wagner et al., 1999b; Beyenburg et al., 2000
		NB	n.d.		n.d.		no	Casey et al., 1994
	Protein	IHC	n.d.		yes	Verma et al., 2013	n.d.	
		WB			n.d.		n.d.	
17βHSD3	mRNA	RT-PCR	n.d.		n.d.		yes	Stoffel-Wagner, 2001
		Comp-RT	n.d.		n.d.		yes	Stoffel-Wagner et al., 1999b; Beyenburg et al., 2000
17βHSD4	mRNA	RT-PCR	n.d.		yes	Konings et al., 2017	yes	Stoffel-Wagner et al., 1999a; Stoffel-Wagner, 2001; Steckelbroeck et al., 2003
		Comp-RT	n.d.		n.d.		yes	Stoffel-Wagner et al., 1999b; Beyenburg et al., 2000
		NB	n.d.		yes	Möller et al., 1999	yes	Möller et al., 1999
17βHSD6	mRNA	RT-PCR	n.d.				yes	Huang and Luu-The, 2000; Steckelbroeck et al., 2003
17βHSD7	mRNA	RT-PCR	n.d.		yes	Törn et al., 2003; Konings et al., 2017	yes	Steckelbroeck et al., 2003
17βHSD8	mRNA	RT-PCR	n.d.		yes	Ohno et al., 2008	yes	Steckelbroeck et al., 2003
17βHSD9	mRNA	RT-PCR	n.d.		n.d.		yes	Steckelbroeck et al., 2003
17βHSD10	mRNA	RT-PCR	n.d.		n.d.		yes	Steckelbroeck et al., 2003; He and Yang, 2006; Hovorkova et al., 2008
	Protein	IHC	n.d.		n.d.		yes	He et al., 2005b
		C-ELISA	n.d.		n.d.		yes	Hovorkova et al., 2008
17βHSD11	mRNA	RT-PCR	n.d.		yes	Chai et al., 2003	yes	Steckelbroeck et al., 2003
	mRNA	NB	n.d.		yes	Chai et al., 2003	n.d.	
	Protein	IHC	n.d.		yes	Brereton et al., 2001	n.d.	
17βHSD12	mRNA	RT-PCR	n.d.		yes	Sakurai et al., 2006; Konings et al., 2017	yes	Sakurai et al., 2006
	mRNA	NB	n.d.		yes	Sakurai et al., 2006	yes	Sakurai et al., 2006
	mRNA	NB	n.d.		n.d.		no	Casey et al., 1994
	Protein	IHC	n.d.		Yes^$^	Sakurai et al., 2006	n.d.	
AKR1C activity			n.d.		n.d.		yes	Steckelbroeck et al., 2010
AKR1C1	mRNA	RT-PCR	n.d.		n.d.		yes	Penning et al., 2000; Stoffel-Wagner et al., 2003; Steckelbroeck et al., 2010
AKR1C2	mRNA	RT PCR	n.d.		n.d.		yes	Penning et al., 2000
		Comp-RT	n.d.		n.d.		yes	Stoffel-Wagner et al., 2003; Steckelbroeck et al., 2004a, 2010
AKR1C3	mRNA	RT PCR	n.d.		yes	Simard et al., 2010; Konings et al., 2017	n.d.	
		Comp-RT	n.d.		n.d.		yes	Stoffel-Wagner et al., 2000, 2003; Steckelbroeck et al., 2001, 2004a; Stoffel-Wagner, 2001
		NB	n.d.		n.d.	Lin et al., 1997	n.d.	
	Protein	IHC	n.d.		yes	Miller et al., 2012b; Chang et al., 2013	n.d.	
AKR1C4	mRNA	RT-PCR	n.d.		n.d.		no	Steckelbroeck et al., 2010
		Comp-RT	n.d.		n.d.		no	Stoffel-Wagner et al., 2000, 2003; Steckelbroeck et al., 2004a
SRD5A1	mRNA	Comp-RT	n.d.		n.d.		yes	Stoffel-Wagner et al., 1998b, 2000, 2003
	Protein	IHC	n.d.		yes	Aumüller et al., 1996	yes	Aumüller et al., 1996
		activity	n.d.		n.d.		yes	Stoffel-Wagner et al., 1998b; Steckelbroeck et al., 2001
SRD5A2	mRNA	Comp-RT	n.d.		n.d.		no	Stoffel-Wagner et al., 1998b, 2000
	Protein	IHC	n.d.		yes	Aumüller et al., 1996	yes	Aumüller et al., 1996
STS	mRNA	RT-PCR	n.d.		yes	Konings et al., 2017	yes	Steckelbroeck et al., 2004b
		RT-PCR	–		no	Miki et al., 2002	no	Miki et al., 2002
	Protein	IHC	n.d.		yes	Iida et al., 2013	yes	Steckelbroeck et al., 2004b
		IHC	–		no	Miki et al., 2002	no	Miki et al., 2002
		Activity	yes	Muir et al., 2004	yes	Milewich et al., 1983; Munroe and Chang, 1987	yes	Platia et al., 1984
SULT1E1	mRNA	RT-PCR	yes	Svoboda et al., 2007	yes	Miki et al., 2002; Konings et al., 2017	yes	Miki et al., 2002; Nishimura and Naito, 2006
		RT-PCR	n.d.		n.d.		no	Salman et al., 2011
		NB	n.d.		n.d.		n.d.	
	Protein	IHC	n.d.		yes	Miki et al., 2002; Iida et al., 2013	yes	Miki et al., 2002
		IHC	n.d.		–		no	Salman et al., 2011
		WB	n.d.		yes	Riches et al., 2009	n.d.	
		Activity	n.d.		yes	Jones et al., 1992	no	Miki et al., 2002
SULT2A1	mRNA	RT-PCR	n.d.		n.d.		no	Nishimura and Naito, 2006; Salman et al., 2011 *3/4 Table VII continues*
	Protein	IHC	n.d.		yes	Riches et al., 2009	no	Steckelbroeck et al., 2004b; Salman et al., 2011
		Activity	n.d.		n.d.		no	Steckelbroeck et al., 2004b.
SULT1A1	mRNA	RT-PCR	n.d.		n.d.		yes	Nishimura and Naito, 2006; Salman et al., 2011
	Protein	IHC	n.d.		n.d.		yes	Nishimura and Naito, 2006; Salman et al., 2011
		WB	n.d.		yes	Riches et al., 2009	n.d.	
SULT2B1	mRNA	RT-PCR	n.d.		yes	He et al., 2004, 2005a	yes	Nishimura and Naito, 2006; Salman et al., 2011
		NB	n.d.		yes	He et al., 2004, 2005a	no	Meloche and Falany, 2001
		NB	–		no	Meloche and Falany, 2001	–	
	Protein	IHC	n.d.		yes	He et al., 2004, 2005a	yes	Nishimura and Naito, 2006; Salman et al., 2011

Primary/original references were analyzed and reviews were excluded (and are cited ad hoc in the text). The table report only the enzymes whose expression was assessed in reviewed studies^**^.

# *Technique abbreviations. For mRNA detection, NB: northern blot; ISH: in situ hybridisation; RT-PCR: reverse transcription quantitative (or semi-quantitative) PCR; Comp-RT: competitive RT-PCR assay; NB: northern blotting. For protein detection: IHC: immunohistochemistry; C-ELISA: competitive ELISA assay; WB: western blotting; activity: enzyme activity measurement*.

* *CYP11A1 and CYP17A1 activities were detected in primary cells of bone*.

^**^No publication was found describing the expression of 17βHSD13, 14, 15 and DHRS11.

∧ *Detected in fetal lung tissue*.

$ *IHC signal in bronchial epithelium*.

#### Bone intracrinology: *in vitro* and *in vivo*

From early ‘90s, various isotopic techniques demonstrated the presence of CYP19A1, 17βHSD reductive/oxidative, 3βHSD and STS activities and the mRNA expression of 17βHSD1, 2, 4, STS, SULT1E1, CYP19A1 and SDR5A in human osteoblastic (e.g., HOS, U20S, HTB-96 and MG63) and osteosarcoma cell lines like CRL-1543 (Purohit et al., 1992; Fujikawa et al., 1997; Jakob et al., 1997; Dong et al., 1998; Saito and Yanaihara, 1998; Janssen et al., 1999; Muir et al., 2004; Svoboda et al., 2007; Dias and Selcer, 2014).

*In vitro* evidence using osteoblastic cells show that E2 has mitogenic effects, which is blocked by the ERα antagonist fulvestrant. Since both E1-S and DHEA-S elicit effects similar to E2, which are blocked by STS inhibition (Selcer and Difrancesca, 2012; Dias and Selcer, 2014), these studies demonstrate that conjugated steroids are activated and that DHEA is converted to E2. Studies in rat osteoblast with [14C]T demonstrated that T is converted by SRD5As and AKR1Cs to 3α/3βDIOLs, which induce proliferation via activation of ER (and not AR) (Enríquez et al., 2013).

*In vitro* models of osteoblast differentiation showed that various differentiation stages are accompanied by declines in STS, CYP19A1 and 17βHSD1 (Janssen et al., 1999; Dias and Selcer, 2016).

In rats, during and after sexual maturation, *in situ* hybridization showed that ERα and ERβ localize in osteoblasts, osteoclasts and osteocytes covering the tibia metaphysis (responsible for elongation of long bones), and co-localize with STS. Starting at sexual maturation (e.g., 7-week-old), ERs also co-localize with CYP19A1, 17βHSD1, 2 and SRD5A1 (van der Eerden et al., 2004). In addition, male transgenic mice overexpressing 17βHSD2 show disturbed IGF-I/steroid actions in bone, with growth retardation, decreased bone formation at prepuberty and decreased serum levels of IGF-I, osteocalcin and T (Shen et al., 2008).

#### Diseases and treatments

Genetic variants of estrogen and intracrine pathways are associated with bone disturbances (Table 5). Defects in the CYP19A1 and ERα are associated with low BMD and other skeletal disturbances (e.g., high stature, delayed bone age) and estrogen therapy ameliorates some bone abnormalities caused by CYP19A1 deficiencies in men (Smith et al., 1994; Morishima et al., 1995; Carani et al., 1997; Mullis et al., 1997; Bilezikian et al., 1998). In lumbar vertebrae, CYP19A1 levels correlate with changes in osteoporotic degree (Sasano et al., 1997).

Inhibitors of 17βHSD2 attracted attention as potential drugs to oppose the effects of low E2 on BMD, fracture and osteoporosis. Ovariectomised female macaques receiving a 17βHSD2 inhibitor display desirable bone balance, bone strength and lower bone resorption compared with untreated controls (Bagi et al., 2008). Several compounds targeting this enzyme have been developed and their use and challenges in osteoporosis were recently reviewed (Soubhye et al., 2015).

In a study on 35 chondrosarcoma biopsies (a malignant bone cancer occurring in middle aged patients), ERα (mRNA and IHC) and CYP19A1 (mRNA and activity) were demonstrated in the majority of the samples, and the AI exemestane impaired the E2- and androgen-induced proliferation of primary chondrosarcoma cells (Cleton-Jansen et al., 2005). Although AIs were proposed as novel drugs to treat this condition (Bovée et al., 2005), a pilot study on six patients with progressive disease showed no benefit of exemestane in progression-free survival compared with untreated patients (Meijer et al., 2011).

In a study of 28 osteosarcoma specimens (one of the most common bone cancers developing at young age) strong ERβ and PR immunoreactivity was seen in over 80% of the samples (and also correlated with Ki67). ERα and AR staining was seen in 30% of the samples, whereas CYP19A1 was undetected (Dohi et al., 2008). In another study, 20 osteosarcoma specimens, including 11 good responders to chemotherapy and nine poor responders, were subjected to cDNA microarray and 17βHSD10 resulted unregulated in the poor responder group. Results were further confirmed by IHC on 69 archival biopsies, hence targeting 17βHSD10 may be a valuable approach for drug (re)sensitisation (Salas et al., 2009).

Additional intracrine imbalances are described in bone diseases, such as higher androgen reducing 17βHSD activity in benign vs. malignant tumors, declines of CYP19A1 from normal bone to osteosarcoma and expression of SULT1E1 in the majority of the skeletal benign and malignant lesions, originated in bones or from primary tumors elsewhere (Svoboda et al., 2007).

#### Bone tissue: conclusions

*In vitro*, animal and human studies show that intracrinology controls bone development, benign and malignant conditions, and offer novel potential drug targets (Table 8 and Figure 3). Steroids can be synthesized *in situ* from cholesterol (Rodríguez-Sanz et al., 2015) and can be recruited from the serum *via* the sulphatase pathway. DHEA is substrate for androgen and estrogen production. The action of androgens is partly mediated by their conversion to estrogens *via* CYP19A1 or to estrogenic 3α/βDIOLs (Vanderschueren et al., 2008).

### Lungs

Sex steroids play an important role in lung development and homeostasis. Androgens, progestogens and estrogens are present and exert genomic and non-genomic actions via their hormone-receptors. Classical ERs (with ERβ as predominant form) and membrane GPER are expressed (Couse et al., 1997; Prossnitz and Barton, 2011; Konings et al., 2017). Sex steroids remain active in the lungs throughout lifetime and modulate lung function in both a beneficial or detrimental way, extensively reviewed (González-Arenas and Agramonte-Hevia, 2012; Townsend et al., 2012; Sathish et al., 2015).

E2 and P regulate epithelial sodium channel expression in alveolar epithelial cells (Luo et al., 2015). In alveolar smooth muscle cells, E2 induces bronchodilation via the reduction of intracellular Ca^2+^ (Townsend et al., 2010).

Both human and animal studies support a promoting role for estrogens and inhibitory role for androgens in lung development and maturation. During gestation and neonatal period, AR is expressed in mesenchymal and epithelial cells. Androgens inhibit the production of surfactants, which starts later in male than in female neonatal lungs (Carey et al., 2007), but also support the developing lung during branching morphogenesis (Kimura et al., 2003).

#### Lung intracrinology in lungs–systematic search

Adult human lungs express CYP19A1 and most 17βHSDs (1, 2, 4, 7, 8, 11, 12, 17βHSD5/AKR1C3; Table 8). STS, SULT and 17βHSD1, 12 and 17βHSD5/AKR1C3 immunoreactivity localizes in the bronchial epithelium (weak for types 1 and 12, strong for type 17βHSD5) and alveolar macrophages (Sakurai et al., 2006; Miller et al., 2012b; Chang et al., 2013; Konings et al., 2017).

Intracrinology controls lung development and maturation as shown in various animal models (Boucher et al., 2009) and intracrine enzymes are expressed already during fetal stages. Human fetal lungs possess StAR, CYP11A1, 3βHSD1 mRNA (Pezzi et al., 2003), SULT1E1 activity (Jones et al., 1992) and show 17βHSD1 and 2 mRNAs expression at 13 and 20 weeks of gestational age (Takeyama et al., 2000). High mRNA levels of AR, 17βHSD2 and 17βHSD5/AKR1C3 in mid-late gestation period and adult lungs indicate the present of androgen metabolism (Simard et al., 2010). Immunoreactivity for 17βHSD11 is detected in bronchioles of 14 and 31 weeks old fetuses, whereas other structures are negative (e.g., alveoli, ciliated epithelium, acini of the trachea). The expression of 17βHSD11 increases during the second half of pregnancy and maintains similar patterns in neonatal (14 days) and adult lugs (Brereton et al., 2001).

#### Intracrinology and lung diseases

Altered intracrinology is involved in lung disorders already from neonatal stages toward adulthood, and SNPs in intracrine genes are associated with the onset of diseases (Zhang et al., 2013). Higher concentration of estrogens were measured by LC-MS in women with multiple-synchronous-lung adenocarcinoma compared with single adenocarcinoma (Ikeda et al., 2016) and in neoplastic tissue compared with adjacent normal lungs (Niikawa et al., 2008; Verma et al., 2013). Type 1 17βHSD mRNA, protein and activity are present in various non-small-cell-lung-cancer (NSCLC) cell lines where the mitogenic effect of E1 is abrogated by 17βHSD1 knockdown (Drzewiecka and Jagodzinski, 2012; Verma et al., 2013). In specimens from 48 NSCLC patients, 17βHSD1 expression was associated with squamous cell carcinoma and stage 3A disease (Drzewiecka et al., 2015). In another study on 103 NSCLC specimens, high 17βHSD1 immunoreactivity was associated with low intratumoural E1 and high E2:E1 ratio, whereas higher 17βHSD2 immunoreactivity was associated with high intratumoural E1. Multivariate regression analysis also demonstrated that increased 17βHSD1 immunoreactivity in tumors was an independent negative prognostic factor (Verma et al., 2013).

CYP19A1 is expressed in lung cancer and has potential therapeutic value (Niikawa et al., 2008; Verma et al., 2011; Siegfried and Stabile, 2014). A recent IHC study on 335 NSCLC specimens found an inverse association between CYP19A1 expression with disease specific survival (Skjefstad et al., 2016). Similar data, although restricted to women only, were confirmed in an independent study on 150 primary lung adenocarcinoma specimens, where CYP19A1 was found as the main driver of local estrogen supply (Tanaka et al., 2016). Another study on 110 lung adenocarcinoma specimens found an association between CYP19A1 mRNA (RT-qPCR) and poor prognosis in females, never-smokers and harboring EGFR mutations (Kohno et al., 2014). However, a recent mRNA study on 96 NSCLC patients showed that CYP19A1 in combination with ER is a good prognostic marker (Aresti et al., 2014).

STS and SULT1E1 immunoreactivity is detected in the majority of NSCLC cases, and STS is a good prognostic marker (Iida et al., 2013).

Lymphangioleiomyomatosis (LAM) is a rare, potentially fatal disease affecting predominantly young women. It is strongly hormone sensitive and it is hypothesized to originate from the uterus as lung metastasis (Prizant and Hammes, 2016). The levels of ERs, PR, AR, CYP19A1, STS, 17βHSD1 and SRD5A2 were recently assessed among 30 LAM biopsies. CYP19A1 expression resulted a useful classification marker with implication for potential therapy (Adachi et al., 2015). A recent study on specimens from 73 patients with chronic obstructive pulmonary disease (COPD) and 48 controls described an association between both CYP19A1 and 17βHSD1 with COPD (Konings et al., 2017). CYP19A1 is also implicated in interstitial pneumonia interstitial pneumonia, where local E2 concentration and CYP19A1 activity and immunoreactivity were elevated in diseased compared with normal tissue (Taniuchi et al., 2014).

#### Potential novel treatments

Blocking the estrogen signaling showed promising preclinical results in animal models of lung cancer (Verma et al., 2011). In humans, antiestrogen treatments (ER antagonists, GnRH, oophorectomy, P) have been used in LAM (Taveira-DaSilva and Moss, 2014) and lung cancer patients (Verma et al., 2011; Kohno et al., 2014). A phase II study on advanced NSCLC patients non-responsive to platinum-based drugs tested the dual-regimen mTOR/CYP19A1 inhibitors. Unfortunately, this study was prematurely terminated due to high toxicity (Singhal et al., 2015) and one additional trial using ER antagonist plus AI (fulvestrant and anastrozole) as consolidation therapy in postmenopausal women with advanced NSCLC (NCT00932152) was terminated due to poor recruitment.

Better results were obtained using the AI letrozole as single agent or in combination with rapamycin in a phase II trial on 17 postmenopausal women with LAM (NCT01353209). AI treatment was safe and well tolerated also in the dual drug regimen (Lu et al., 2017).

#### Lungs: conclusions

Steroids are involved in lung maturation, development and in susceptibility to diseases. Most 17βHSDs, STS/SULT1E1, CYP19A1 are expressed indicating the lung's ability to metabolize androgens, estrogens and progestogens. Evidence of 3βHSDs is limited to fetal tissues (Table 8 and Figure 3). Approaches aimed at decreasing local estrogens may offer future novel treatments for various lung diseases.

### Brain and central nervous system (CNS)

One of the first CNS actions of sex steroids to be described is the hypothalamus-pituitary-gonadal axes control (Andersen and Ezcurra, 2014). The identification of steroid-receptors outside the hypothalamus, like hippocampus (controlling memory), prefrontal cortex, cerebellum and dopaminergic system regulation indicated that sex steroids have complex and widespread effects in the CNS. They control aggressive behavior, cognitive functions, mood, food intake, appetite, addiction, blood pressure, fine motor skills, motor coordination, pain circuit and both estrogens and androgens are neuroprotective (López and Tena-Sempere, 2015; Soma et al., 2015; McEwen and Milner, 2017). Estrogen deprivation in animals and humans is associated with development of metabolic disorders and estrogen administration has a general catabolic effect (López and Tena-Sempere, 2015). Animal experiments and KO models show that ERα mediates the major actions of estrogens in the CNS, like the metabolic control functions (Musatov et al., 2007) and the negative-feedback on the hypothalamus-pituitary-gonadal axes (Couse et al., 2003). However, both nuclear and non-nuclear ERs are relevant in distinct CNS regions (Almey et al., 2015; López and Tena-Sempere, 2015; McEwen and Milner, 2017).

Local steroid synthesis in the CNS is demonstrated in animal studies. CYP19A1-KO mice have increased ischemic damages compared with ovariectomised wild-type mice, indicating a local action of CYP19A1 (McEwen and Milner, 2017). Similar conclusions were drawn for the estrogen protective effects on stroke, Alzheimer (AD), Parkinson diseases, aggressive behavior (Soma et al., 2015; McEwen and Milner, 2017) and mice with ablation in various 17βHSDs show neuronal defects (Table 4). In rodents, CNS regions like the hippocampus and the hypothalamus express the enzymes involved in the local generation of steroids, like StAR, CYP11A1, CYP17A1, 3βHSD1, CYP19A1, 17βHSD1, SRD5A1 and 2 (mRNA and protein by immunohistochemistry or western blot), and can produce pregnenolone, DHEA, androgens and estrogens from cholesterol, as confirmed by HPLC using radiolabelled substrates and tissue cultures of brain slices (Mukai et al., 2006; Murakami et al., 2006). CYP enzymes of rat hippocampus co-localize in pyramidal neurons (CA1–CA3 regions) and granule cells (dentate gyrus) (Mukai et al., 2006; Murakami et al., 2006). Regulation of intracrine enzymes varies during development and sexual maturation, as indicated by mRNA expression (RT-qPCR) of 20 intracrine enzymes analyzed in rat hippocampus post-natal and throughout early (1 week) development (Kimoto et al., 2010).

Intracrinology in the CNS is particularly relevant because, beside the traditional pathway *via* the receptors, several steroids have neuroactivity and are allosteric modulators of GABA_A_ receptors (Figure 2). Such actions are possessed also by steroids that are unable to activate the steroid hormone receptors, such as 3β- and 3α-hydroxyl sulpho-conjugates (P5-S and DHEA-S), 5β-reduced steroids (5βAN, etiocholanolone and 5β-THP isomers; Table 1), which are all GABA_A_ negative modulators (in contrast to unconjugated 3α-hydroxysteroids) (Stoffel-Wagner, 2001; Belelli and Lambert, 2005; Agís-Balboa et al., 2006; Gibbs et al., 2006; Reddy, 2010; Steckelbroeck et al., 2010).

#### Intracrinology in CNS–systematic search

Intracrine enzymes are widely expressed in human CNS (Table 8) and intratissue concentrations of steroids in distinct regions differ between regions and from the levels in the blood (Mukai et al., 2006; Murakami et al., 2006; Jäntti et al., 2010). In contrast to rodents, however, the presence of the complete steroid biosynthetic pathway is not clearly demonstrated in the human CNS and contrasting data were reported (Table 8). CYP11A1 mRNA was detected in the temporal, frontal neocortex and subcortical white matter of men, women and children (Stoffel-Wagner, 2001). Low mRNA levels of CYP17A1 were detected in the hippocampus, amygdala, caudate nucleus, cerebellum, corpus callosum, spinal cord and thalamus (Stoffel-Wagner, 2001; Yu et al., 2002), but other authors found no expression of this enzyme (Steckelbroeck et al., 2004b, 2010; MacKenzie et al., 2008). No 3βHSD1 or 2 was detected in temporal lobes, hippocampus, thalamus and amygdala (Stoffel-Wagner, 2001; Steckelbroeck et al., 2010), although other authors detected low levels in amygdala, caudate nucleus, cerebellum, corpus callosum, hippocampus, spinal cord and thalamus (Yu et al., 2002).

The temporal lobes (both neocortex and white matter) have 17βHSD oxidative and reductive activities, CYP19A1 mRNA expression and activity, which is also present in hippocampus (Stoffel-Wagner et al., 1999a; Stoffel-Wagner, 2001). Temporal lobe specimens from 10 men to 12 women indicated that 17βHSD estrogen-oxidative and DHEA-reductive metabolisms are predominant, thus producing E1 and A5, respectively (Stoffel-Wagner, 2001). Regarding the different 17βHSDs, type 1, 3, and 4 mRNAs (but not type 2) were demonstrated by competitive reverse transcription-PCR in specimens from 34 women, 32 men and 10 children (Casey et al., 1994; Beyenburg et al., 2000). Subsequent studies confirmed the expression of types 4, 7, 8, 10, 11 17βHSD and AKR1C3/17βHSD5 in temporal lobes and hippocampus (Stoffel-Wagner, 2001; Steckelbroeck et al., 2003). In particular 17βHSD10 is involved in the deactivation of THP to 5αDHP, and it is an important regulator of neurological functions (Yang et al., 2016).

Production of 5α-androstane and pregnane neurosteroids is mediated by the action of SRD5As and AKR1Cs (Figure 2). SRD5A1 (not type 2) mRNA and enzyme activity were demonstrated in temporal neocortex and subcortical white matter, hippocampus, cerebellum, hypothalamus (Steckelbroeck et al., 2001; Stoffel-Wagner, 2001), and AKR1C4 mRNA was detected in both hippocampus and temporal lobe (Stoffel-Wagner, 2001). AKR1C1 and AKR1C2 are widely expressed in CNS and since no specific inhibitors directed against AKR1C1 to 4 could completely inhibit AKR1C brain activity, the involvement of an unidentified enzyme is suggested (Steckelbroeck et al., 2010). Isomeric 5β-neurosteroids require the action of AKR1D1, and it is unknown whether AKR1D1 is expressed in CNS, or liver 5β-steroids reach peripheral regions via the circulation (Jin et al., 2011).

The sulphatase pathway in the CNS is relevant because (although recent studies are revisiting this paradigm; Qaiser et al., 2017), sulphated-steroids do not cross the blood-brain barrier. Therefore, sulphated neurosteroids like DHEA-S and P5-S need to be generated locally, and in line with this, their level in the CNS is independent from the level in the blood (Rajkowski et al., 1997) and varies throughout distinct brain regions (especially hippocampus and hypothalamus) (Jäntti et al., 2010).

STS and SULTs are widely expressed, with no gender-related differences (Table 8) (Kríz et al., 2008a,b; Mueller et al., 2015). SULT1A1 has high expression especially in specimens isolated from cerebellum, occipital and frontal lobes (Salman et al., 2009). No brain region expresses SULT2A1, whereas contrasting data exist for SULT2B1 and SULT1E1 (Table 8).

#### Diseases and treatments

Steroid metabolism is deviated in schizophrenia (Bicikova et al., 2013) and aberrations and unbalances of intracrine enzymes are associated with neurological disorders (Luchetti et al., 2011 and see Table 5). In a study of 49 patients with AD, prefrontal cortex mRNA levels of 17βHSD1, CYP19A1 and AKR1C2 increased at late stages (Luchetti et al., 2011). STS and SULT activities, measured by radioimmunoassay and GC-MS in 55 human brain tumor specimens, varied between tumor types (Kríz et al., 2008b). Immunoreactivity for AKR1C3/17βHSD5 was low in medulloblastomas (*n* = 10 analyzed), high in 37 glial neoplasms and 18 meningiomas and was absent in intracranial schwannoma (*n* = 7) (Park et al., 2010). A recent screening of a chemical library of steroid inhibitors using three low grade pediatric glioma cell lines found that inhibition of 17βHSD3 blocked cell growth and induced apoptosis *in vitro* (Ajeawung et al., 2013)

Type 10 17βHSD is associated with AD and is a potential target in diseases like AD, Parkinson, and an X-linked mental retardation, that may arise from the impaired degradation of branched chain fatty acid, isoleucine or aberrant neurosteroid (THP) metabolism (Lim et al., 2011; Yang et al., 2016).

STS has been implicated in ADHD and a recent mouse study indicates that genetic and pharmacological manipulations of the STS axis influence the inhibitory processes and give rise to improvements in response control (Davies et al., 2014). A recent animal experiment using a model of autoimmune encephalomyelitis showed high SULT1A1 mRNA expression in laser-captured-micro-dissected white matter astrocytes, suggesting that deactivation of estrogens (and other phenolic substrates) may be responsible for the resistance to anti-neuro-inflammatory treatments in these cells and could be possibly used as new treatments to protect CNS from inflammatory injuries (Guillot et al., 2015).

#### CNS: conclusions

CNS can synthesize steroids from cholesterol, although this is restricted to few brain regions. Steroid metabolism in the CNS is particular complex due to the formation of both 5α-/β-reduced and sulpho-conjugated neurosteroids (Table 8 and Figure 3).

### Intracrinology in other tissues and systems

Steroid metabolism is also important in the immune system, skin and adipose tissue. A thorough review of these systems is outside the scope of this study, however, a brief mention is given below.

#### Immune system and inflammation

Beside corticosteroids, several other steroids affect the immune system and inflammation. A5 induces white blood cells and platelets production in bone marrow (Chen et al., 2004); estrogens and androgens control B-lymphocyte development in a sex-dependent way and modulate autoimmune diseases (McCarthy, 2000; Calippe et al., 2010; Sakiani et al., 2013).

Lipopolysaccharide-mediated proinflammatory pathway in macrophages and NF-κB activation are blocked by estrogens, which induce T-helper (Th) type 2 responses, whereas androgens stimulate type 1 responses (Iwasa et al., 2015). DHEA and DHEA-S also regulate the maturation of Th1 or Th2 cells. It was shown that plasma Th2 lymphocytes and its major secreted cytokine IL6 increase with age, and this is reversed in mice upon administration of DHEA or DHEA-S (Reed et al., 2005). Such effect was recapitulated *in vitro* by DHEA but not DHEA-S implicating the involvement of macrophage STS in lymphoid tissues where Th cell maturation occurs. In line with this, the effect of DHEA-S, but not DHEA, was impaired *in vivo* by an STS inhibitor (Reed et al., 2005). These data prompted to propose STS inhibition as a therapeutic approach for diseases associated with inappropriate immune responses and excess Th1 cytokines such as rheumatoid arthritis (Reed et al., 2005). Whether the action of DHEA is secondary to its conversion to androgens or estrogens is currently unclear. STS activity of peripheral blood leukocytes is higher in women during the follicular phase of the menstrual cycle than in women in the luteal phase or in men and it becomes highest during pregnancy, suggesting a role for P in regulating STS activity (Reed et al., 2005; Mueller et al., 2015). *In vitro* studies also demonstrated that STS activity is induced by cytokines such as IL6 and TNF (Mueller et al., 2015).

Opposite deregulation of the sulphatase pathway is seen in other chronic inflammatory diseases/cell types. Vascular smooth muscle cells show higher STS activity in women with mild atherosclerosis compared with women with severe disease (and male), whereas SULT1E1 activity is lower in women with severe disease (Mueller et al., 2015).

CYP19A1 is also expressed in macrophages (Konings et al., 2017) and KO mice have increased numbers of peripheral blood and bone marrow cells and inflammatory renal lesions (Shim et al., 2004). CYP19A1 inhibitors exacerbate the autoimmune lesions in a murine model of Sjögren syndrome and estrogen administration reverses such phenotype (Iwasa et al., 2015; Park et al., 2015). Opposite effects are observed in prostate, where elevated intracrine estrogens due to CYP19A1 overexpression induce inflammation and pre-malignant pathology (Ellem et al., 2009) as well as in adipose tissue (Reed et al., 2005).

#### Skin

The skin is the largest human organ and first barrier against pathogens where important immune functions interconnected with intracrine steroid metabolism take place (Slominski et al., 2013). Keratinocytes and sebocytes express ERs, intracrine enzymes, and the activity of sebaceous glands is influenced by steroids as indicated by the sebum production at andrenarche (Slominski et al., 2013). CYP17A1, CYP19A1, 17βHSD1, 2, 3, 4 (and enzymes metabolizing corticosteroid - outside the scope of this review) are detected in human skin. Some genes are under the influence of vitamin D and sebocytes can synthesize T from adrenal precursors (Hughes et al., 1997; Thiboutot et al., 1998; Slominski et al., 2013). Low 17βHSD oxidative metabolism characterizes sebaceous glands from skin areas prone to develop acne compared with other locations, suggesting a protective role of the oxidative metabolism against androgen excess (Fritsch et al., 2001). Sulphatase pathway is present in the skin (Reed et al., 2005; Simard et al., 2005), and genetic variants in STS and SULTs are associated with skin disturbances, most likely because of unbalanced steroid accumulation (Table 5).

#### Adipose tissue

The adipose tissue is one of the most complex endocrine organs that besides secreting leptin and adiponectin, is a site of steroid metabolism, it establishes interaction with the CNS for glucose and lipid metabolism control, energy homeostasis and inflammation. The implication of sex steroids in adipose tissue is demonstrated by the different fat distribution that characterizes men and women (Mauvais-Jarvis et al., 2013; Varlamov et al., 2014; López and Tena-Sempere, 2015, 2016; Palmer and Clegg, 2015). ER-KO and CYP19A1-KO mice develop obesity with human-like phenotypes (López and Tena-Sempere, 2015). Estrogens protect against metabolic syndrome and men lacking endogenous estrogens (CYP19A1 or ER-α deficiency) develop hypertriglyceridemia, glucose intolerance and insulin resistance (Kim et al., 2014). In adipose tissue of men, 17βHSD2 levels and androgen inactivation correlate with BMI (Fouad Mansour et al., 2015). A mouse study also showed that increased unsulphated-estrogen availability due to loss of SULT1E1 improved metabolic function in a model of type 2 diabetes, which leads to speculations about a potential role of SULT1E1 inhibition for this disease - at least in women (Gao et al., 2012).

Fat consists of different tissue types (white and brown) and different regional depots with distinct physiological, intracrinological characteristics and distinct relations with pathologies and metabolic disorders (Blouin et al., 2009; Mauvais-Jarvis et al., 2013). White adipose tissue is mainly subcutaneous (abdomen) or visceral (surrounding the inner organs), this last being associated with metabolic risks. A plethora of investigations demonstrated the ability of adipose tissue to aromatise androgens into estrogens and that the intra-tissue steroid levels are higher than the levels in blood (Bélanger et al., 2002). Androgenic and estrogenic 17βHSD activity and the mRNA for 17βHSD1, 2, 3, 7, 12, AKR1C3/17βHSD5 were detected in both intra-abdominal and subcutaneous fat (Bélanger et al., 2002; Quinkler et al., 2004; Bellemare et al., 2009; Wang et al., 2013).

Both subcutaneous and visceral fat tissue of women expresses the androgenic 17βHSD3 (generally considered testis specific) indicating that adipose tissue in women is substantially androgenic. Such characteristic in the visceral depot increased with increasing BMI, suggesting a link between central obesity and metabolic diseases (Corbould et al., 2002).

Additionally, several enzymes (AKR1C2, AKR1C3/17βHSD5, CYP19A1, STS and SULT1E1) vary throughout adipocyte differentiation and maturation (Quinkler et al., 2004; Bellemare et al., 2009; Blouin et al., 2009; Mueller et al., 2015).

## Conclusions and recommendations

Intracrinology consists of a complex and intricate network of alternative and redundant pathways that generate, deactivate steroids in peripheral tissues and ultimately control steroid exposure in a tissue specific manner. A number of compounds have that ability to bind and activate more than one nuclear receptors thus exerting multiple biological actions. Blood steroids represent a reservoir of substrates that support these intracrine networks. Studies retrieved by the systematic search demonstrated that most investigations rely on RT-PCR or IHC to detect enzyme and protein, and frequently without multiple-technique confirmation of the data. Since both techniques present limitations, and antibodies for IHC often perform sub-optimally (detection limit is not sufficient to detect some intracrine enzymes, crossreactivity between isoforms) these techniques are not always suitable to infer the real biological role of a reaction/enzyme.

However, the recent technological advances in steroid profiling together with an improved knowledge of intracrine enzymes and the possibility to validate data using multiple approaches (RNA, protein, activity, steroid profiling) create today unprecedented opportunities to expand our understanding of intracrinology, its relation with endocrinology and to exploit this knowledge in patient care. Improved multiplex platforms allowing to profile in peripheral tissues all steroids depicted in Figure 2 are awaited and will elucidate the relevant tissue-specific networks. It is envisaged that novel prognostic markers and drug targets will become of clinical relevance soon.

We should however be aware that the redundant actions of intracrine enzymes, their substrate promiscuity, the existence of alternative pathways and the patient-to-patient variability might result in drug insensitivity. Dual/triple inhibitors will help solving this problem. In addition, in order to optimize research on novel drugs, the classical preclinical drug discovery pipelines (safety, pharmacokinetics and dynamics), should encompass parallel research lines to learn how to pre-select potentially responsive patients.

Finally, since we know that steroidal and intracrine drugs might have profound effects on the CNS, it is desirable to have in depth research on the neurological effects of potential novel drugs during the nonclinical phase of drug development. This will facilitate to select suitable compounds to the clinical development.

## Author contributions

GK drafted the study, prepared figures, tables, intermediate versions, final version and approved final version. LB drafted part of the study, contributed to intermediate versions and approved final version. KC drafted part of the study, contributed to intermediate versions and approved final version. BD contributed to intermediate versions and approved final version. TL drafted part of the study, contributed to intermediate versions and approved final version. PK contributed to intermediate versions and approved final version. MB contributed to intermediate versions and approved final version. RK contributed to intermediate versions and approved final version. SX drafted part of the study, contributed to intermediate versions and approved final version. AR drafted the study, prepared figures, tables, intermediate versions, final version and approved final version.

### Conflict of interest statement

PK and TL are employees of Forendo Pharma Ltd. The remaining authors declare that the research was conducted in the absence of any commercial or financial relationships that could be construed as a potential conflict of interest.

## Abbreviations

[3H]: tritiated hydrogen
[14C]: radioactive carbon
AD: Alzheimer disease
ADHD: attention deficit hyperactivity disorder
AI: aromatase inhibitor
AKR: aldo-ketoreductase
AR: androgen-receptor
ART: assisted reproduction technology
BMD: bone mineral density
BMP-2: bone morphogenetic protein 2
COPD: chronic obstructive pulmonary disease
COUP-TFII: chicken-ovalbumin-upstream-promoter-transcription-factor II
CNS: central nervous system
CRC: colorectal cancer
CX43: connexin 43
DS: digestive system
Ed: embryonic day
EC: endometrial cancer
EndRet: endoplasmic reticulum
ER: estrogen-receptor
Fdx: ferredoxin
FdR: ferredoxin reductase
GC-MS: gas-chromatography tandem mass-spectrometry
GnRH: gonadotropin releasing hormone
GH: growth hormone
GIT: gastrointestinal tract
GPER: G protein-coupled estrogen-receptor
HPLC: high performance liquid-chromatography
HRT: hormone-replacement-therapy
hCG: human chorionic gonadotropin
IGF1: insulin-like growth factor 1
IHC: immunohistochemistry
IL-1: interleukin-1
IL-6: interleukin-6
KO: knock-out
LC-MS: liquid-chromatography tandem mass-spectrometry
mTOR: mammalian target of rapamycin
NAPDH: nicotine-adenine-dinucleotide-phosphate
NSCLC: non-small cell lung cancer
PAIN: phenomena of pan-assay interfering compounds
PAP: bis-phospho-nucleotide-3′-phospho-adenosine-5′-phosphate
PCOC: poly cystic ovarian syndrome
POR: P450 oxidoreductase
RT-qPCR: reverse-transcriptase quantitative polymerase-chain-reaction
SF1: steroidogenic factor 1
SHBG: sex hormone binding globulin
SNP: single nucleotide polymorphism
SRD: short-chain dehydrogenase
Th: T-helper
TNF: tumor necrosis factor
VEGF: vascular endothelial growth factor
WB: western blot/blotting
WOI: window of implantation.
