# Supplementary material for: Intracrine Regulation of Estrogen and Other Sex Steroid Levels in Endometrium and Non-gynecological Tissues; Pathology, Physiology, and Drug Discovery
Source: Front Pharmacol. 2018 Sep 19;9:940. doi: 10.3389/fphar.2018.00940 (PMC6157328; doi:10.3389/fphar.2018.00940)

PANEL I: ALIASES FOR COMPOUND NAMES

5α-pregnan-20-ol-3-one
- allopregnan-20α-ol-3-one
- 20α-hydroxy-5α-pregnan-3-one

allopregnandiol
- 5α-pregnane-3α,20α-diol (isomer 1/4)
- allopregnan-3α,20α-diol (isomer 1/4)
- 3α,20α-dihydroxy-5α-pregnane (isomer 1/4)
- 5α-pregnane-3β,20β-diol (isomer 2/4)
- 5α-pregnane-3α,20β-diol (isomer 3/4)
- 5α-pregnane-3β,20α-diol (isomer 2/4)

5αDHP
-allopregnanedione
-5α-pregnane-3,20-dione
- 3,20-allopregnanedione
- 3,20-dioxo-5α-pregnane
- 5α-dihydroprogesterone

5α-pregnan-17α-ol-3,20-dione
- 17α-hydroxy-5α-pregnane 3,20-dione
- allopregnane-3,20-dione-17α-ol

THP (isomer 1/4)
- allopregnanolone
- 3α-hydoxy-5α-pregnan-20-one
- (3α,5α)-3-hydroxypregnan-20-one
- 3α-OH DHP
- 5α-pregnan-3α-ol-20-one
- allopregnan-3α-ol-20-one
- (+)-3α-hydroxy-5α-pregnan-20-one
- (3α)-allopregnanolone
- 3α-tetrahydroprogesterone
- 3α,5α-tetrahydroprogesterone
- 3α,5α-pregnanolone
- Pregnanolone, (5α)-isomer
- brexanolone

THP (isomer 2/4)
- pregnanolone
- 3α-hydroxy-5β-pregnan-20-one
- 3α,5β-tetrahydroprogesterone
- 3α,5β-THP
- eltanolone
- pregnan-3α-ol-20-one
- β-pregnan-20-one, 3α-hydroxy-5

THP (isomer 3/4)
- isopregnanolone
- 3β-hydoxy-5α-pregnan-20-one
- 3β,5α-tetrahydroprogesterone
- 3β,5α-THP
- isoallopregnanolone
- epiallopregnanolone
- allopregnan-3β-ol-20-one
- β-ol-20-one, allopregnan-3
- 3-pregnan-20-one, 3α-hydroxy-5
- eltanolone
- Pregnanolone, (3β, 5α)-isomer

THP (isomer 4/4)
- epipregnanolone
- 3β-hydoxy-5β-pregnan-20-one
- 3β,5β-tetrahydroprogesterone
- 3β,5β-THP
- 3β-hydroxy-5β-tetrahydroprogesterone
- pregnanolone I
- sepranolone
- β-ol-20-one, allopregnan-3

P
- progesterone
- 4-pregnene-3,20-dione
- pregn-4-en-3,20-dione
- agolutin
- akrolutin
- corpus luteum hormone
- crinone
- Δ4-pregnene-3,20-dione

17OHP
- 17α-hydroxyprogesterone
- 17-hydroxypregn-4-en-3,20-dione
- 17α-hydroxy-4-pregnene-3,20-dione
- 17α-hydroxy-progesterone
- Δ4-pregnene-17α-ol-3,20-dione
- hydroxyprogesterone
- pregn-4-ene-3,20-dione-17-ol

20αDHP
- 20α-dihydroprogesterone
- 20α-hydroxypreg-4-en-3-one
- 20α-hydroxydihydroprogesterone
- 20α-hydroxy-4-pregnen-3-one
- 20α-progerol
- 20α-hydroxypregnen-2-one
- progesterol-20α
- algestone
- alphasone
- pregn-4-en-3-one, 20α-hydroxy

17OHP5
- 17-hydroxypregnenolone
- 5-Pregnen-3β,17α-diol-20-one
- (3β)-3,17-dihydroxypregn-5-en-20-one
- 17-OH-pregnenolone
-17α-hydroxypregnolone
- 3β,17-dihydroxy-5-pregnen-20-one
- hydroxypregnenolone

P5
- pregnenolone
- 3β-hydroxy-5-pregnen-20-one
- 3-hydroxypregn-5-en-20-one
- 5-Pregnen-3β-ol-20-one
- natolone
- Pregn-5-en-3β-ol-20-one
- pregneton

Androstanedione
- 5α-androstane-3,17-dione
- 5α-androstanedione
- androstane-3,17-dione
- 5α-androstane-3,17-dione
- androstane-3,17-dione, (5α)-isomer
- 3,17-dioxy-5α-androstane

AN
- androsterone
- 3α-hydroxy-5α-androstan-17-one
- 3α-hydroxy-17-androstanone
- (3α,5α)-3-hydroxy-androstan-17-one
- 3α-hydroxyetioallocholan-17-one
- 3-epihydroxyetioallocholan-17-one
- 3-hydroxyandrostan-17-one
- androkinine

3αDIOL
- androstanediol
- 5α-androstane-3α,17β-diol
- 3α-androstanediol
- androstane-3,17-diol
- 5-androstane-3,17-diol

3βDIOL
- 3β-androstanediol
- 5α-androstane-3β,17β-diol

EPI
- epiandrosterone
- 3β-hydroxy-17-oxo-5α-androstane
- 3-epiandrosterone
- 3β-androsterone
- 3β-hydroxy-3β-androstan-17-one
- 3β-hydroxyandrostan-17-one
- 3β-hydroxyetioallocholan-17-one
- 3β-OH-5α-androstane-17-one
- 5α-androstan-3β-ol-17-one
- D-epiandrosterone

DHT
- Dihydrotestosterone
- 5α-androstan-17β-ol-3-on
- 5α-dihydrotestosterone
- 17β-hydroxy-5α-androstan-3-one
- 17β-Hydroxy-3-oxo-5α-androstanone
- androstanolona
- androstanolone
- stanolone

EpiT
- epitestosterone
- 17α-hydroxy-4-androsten-3-one
- isotestosterone
- 17α-testosterone
- androst-4-en-17α-ol-3-one

A5
- androstenediol
- androst-5-ene-3β,17β-diol
- 5-androstenediol
- androst-5-ene-3,17-diol
- Tetrabol
- (3β,17β)-androst-5-ene-3,17-diol
- Δ5-androstenediol
- Δ5-androstene-3β,17β-diol

Δ4-A5
- 4-androstenediol
- androst-4-ene-3β,17β-diol
- Δ4-androstene-3β,17β-diol
- (3β,17β)-androst-4-ene-3,17-diol
- 3β,17β-dihydroxy-4-androstene

DHEA
- Dehydroepiandrosterone
- dehydroisoandrosterone
- (3β)-3-hydroxyandrost-5-en-17-one
- 17-hormoforin
- 3β-hydroxy-5-androsten-17-one
- 3β-Hydroxy-Δ5-androsten-17-one
- andrestenol
- androstenolone

A4
- androstenedione
- 4-Androstene-3,17-dione
- 3,17-dioxoandrost-4-ene
- Δ4-androstenedione
- Androst-4-ene-3,17-dione
- 4-androstene-3,17-dione
- Δ4-dione
- Δ4-Androsten-3,17-dione
- 17-ketotestosterone

T
- testosterone
- 17β-hydroxyandrost-4-ene-3-one

E2
- 17β-estradiol
- (17β)-estra-1,3,5(10)-triene-3,17-diol
- cis-estradiol
- 13β-methyl-1,3,5(10)-gonatriene-3,17β-ol
- 3,17-epidihydroxyestratriene
- 3,17β-dihydroxyestra-1,3,5(10)-triene
- aerodiol

E1
- estrone
- 3-hydroxy-1,3,5(10)-estratrien-17-one
- follicular hormone
- 1,3,5(10)-estratrien-3-ol-17-one
- 3-hydroxy-17-keto-estra-1,3,5-triene
- 3-hydroxyestra-1,3,5(10)-trien-17-one
- Δ1,3,5(10)-estratrien-3-ol-17-one

PANEL II: ALIASES FOR ENZYME NAMES (1/2)

StAR
-Steroidogenic Acute Regulatory Protein
- Mitochondrial Steroid Acute Regulatory Protein
- StAR Related Lipid Transfer (START) Domain Containing 1
- Cholesterol Trafficker

CYP11A1
- Cytochrome P450 Family 11 Subfamily A Member 1
- Cytochrome P450, Subfamily XIA (Cholesterol Side Chain Cleavage)
- Cytochrome P450 11A1
- Cytochrome P450 side-chain cleavage enzyme
- Cholesterol 20-22 Desmolase
- Cholesterol Desmolase
- Cholesterol Monooxygenase (Side-Chain-Cleaving)

CYP17A1
- Cytochrome P450c17
- Cytochrome P450 Family 17 Subfamily A Member 1
- Steroid 17-α-Monooxygenase
- Cytochrome P450, Family 17, Subfamily A, Polypeptide 1
- Steroid 17-α-Hydroxylase/17,20 Lyase
- 17-α-Hydroxyprogesterone Aldolase

CYP19A1
- Aromatase
- Cytochrome P450 Family 19 Subfamily A Member 1
- Cytochrome P450, Subfamily XIX
- Cytochrome P-450AROM
- Cytochrome P450 19A1
- Estrogen Synthase
- Microsomal Monooxygenase
- Flavoprotein-Linked Monooxygenase

HSD3B1
- 3β-Hydroxysteroid Dehydrogenase/Δ5→4-Isomerase Type I
- Δ^{5-4}-isomerase
- Δ^{5}-3β-HSD
- Hydroxy-Δ^{5}-Steroid Dehydrogenase, 3β- and Steroid Δ-Isomerase 1
- Short Chain Dehydrogenase/Reductase Family 11E, Member 1
- Trophoblast Antigen FDO161G
- 3β-Hydroxy-Δ(5)-Steroid Dehydrogenase
- 3β-Hydroxy-5-Ene Steroid Dehydrogenase
- Δ^{5}-3-Ketosteroid Isomerase
- Steroid Δ-Isomerase
- Progesterone Reductase

HSD3B2
- 3β-Hydroxysteroid Dehydrogenase/Delta 5-->4-Isomerase Type II
- Δ^{5-4}-isomerase
- Δ^{5}-3β-HSD
- Hydroxy-Δ-5-Steroid Dehydrogenase, 3 β- And Steroid Δ-Isomerase 2
- Short Chain Dehydrogenase/Reductase Family 11E, Member 2
- 3β-HSD Adrenal And Gonadal Type
- β-Hydroxysteroid Dehydrogenase Type II
- 3-β-Hydroxy-Δ(5)-Steroid Dehydrogenase
- 3-β-Hydroxy-5-Ene Steroid Dehydrogenase
- Delta 5-Δ 4-Isomerase Type II
- Progesterone Reductase

17βHSD1
- 17β-hydroxysteroid dehydrogenase type 1
- Short Chain Dehydrogenase/Reductase Family 28CE, Member 1
- Hydroxysteroid 17-β-Dehydrogenase 1
- Placental 17-β-Hydroxysteroid Dehydrogenase
- 20 Alpha-Hydroxysteroid Dehydrogenase
- Estradiol 17-β-Dehydrogenase-1

17βHSD2
- 17β-hydroxysteroid dehydrogenase type 2
- Short Chain Dehydrogenase/Reductase Family 9C Member 2
- Hydroxysteroid 17-β-Dehydrogenase 2
- Microsomal 17-β-Hydroxysteroid Dehydrogenase
- 20 Alpha-Hydroxysteroid Dehydrogenase
- Testosterone 17-β-Dehydrogenase

17βHSD3
- 17β-hydroxysteroid dehydrogenase type 3
- Short Chain Dehydrogenase/Reductase Family 12C Member 2
- Hydroxysteroid 17-β-Dehydrogenase 3
- Testicular 17-β-Hydroxysteroid Dehydrogenase
- Testosterone 17-β-Dehydrogenase 3

17βHSD4
- 17β-hydroxysteroid dehydrogenase type 4
- Short Chain Dehydrogenase/Reductase Family 8C Member 1
- 3-Alpha,7-Alpha,12-Alpha-Trihydroxy-5-β-Cholest-24-Enoyl-CoA Hydratase
- 17β-Estradiol Dehydrogenase Type IV
- Peroxisomal MultiCatalysisal Protein 2
- D-BiCatalysisal Protein, Peroxisomal
- D-3-Hydroxyacyl-CoA Dehydratase
- β-Hydroxyacyl Dehydrogenase
- MultiCatalysisal Protein 2
- β-Keto-Reductase2

17βHSD6
- 17β-hydroxysteroid dehydrogenase type 6
- Short Chain Dehydrogenase/Reductase Family 9C Member 6
- Oxidative 3-Alpha-Hydroxysteroid-Dehydrogenase
- 3(Alpha->Beta)-Hydroxysteroid Epimerasel
- 3-Hydroxysteroid Epimerase
- Retinol Dehydrogenase
- Oxidoreductase
- NAD+ -Dependent 3 Alpha-Hydroxysteroid Dehydrogenase
- Oxidative 3-Alpha Hydroxysteroid Dehydrogenase

17βHSD7
- 17β-hydroxysteroid dehydrogenase type 7
- Short Chain Dehydrogenase/Reductase Family 37C Member 1
- Estradiol 17-Beta-Dehydrogenase 7
- 3-Keto-Steroid Reductase

17βHSD8
- 17β-hydroxysteroid dehydrogenase type 8
- Short Chain Dehydrogenase/Reductase Family 30C Member
- 3-Ketoacyl-[Acyl-Carrier-Protein] Reductase Alpha Subunit
- 3-Oxoacyl-[Acyl-Carrier-Protein] Reductase
- Really Interesting New Gene 2 Protein
- Testosterone 17-Beta-Dehydrogenase 8
- Estradiol 17-Beta-Dehydrogenase 8
- Estrogen 17-Oxidoreductase

17βHSD9
- 17β-hydroxysteroid dehydrogenase type 9
- Short Chain Dehydrogenase/Reductase Family 9C Member 5
- Retinol Dehydrogenase 5
- 9-Cis Retinol Dehydrogenase
- 11-Cis Retinol Dehydrogenase

17βHSD10
- 17β-hydroxysteroid dehydrogenase type 10
- Endoplasmic Reticulum-Associated Amyloid Beta-Peptide-Binding Protein
- 3-Hydroxy-2-Methylbutyryl-CoA Dehydrogenase
- Mitochondrial Ribonuclease P Protein 2
- AB-Binding Alcohol Dehydrogenase
- Mitochondrial RNase P Subunit 2
- 3-Hydroxyacyl-CoA Dehydrogenase Type II

PANEL II: ALIASES FOR ENZYME NAMES (2/2)

17β HSD11
- 17β-hydroxysteroid dehydrogenase type 11
- Short Chain Dehydrogenase/Reductase Family 16C Member 2
- Retinal Short-Chain Dehydrogenase/Reductase 2
- Cutaneous T-Cell Lymphoma-Associated Antigen HD-CL-03
- Dehydrogenase/Reductase SDR Family Member 8
- Estradiol 17-Beta-Dehydrogenase 11

17β HSD12
- 17β-hydroxysteroid dehydrogenase type 12
- Short Chain Dehydrogenase/Reductase Family 12C Member 1
- Estradiol 17-Beta-Dehydrogenase 12
- Very-Long-Chain 3-Oxoacyl-CoA Reductase
- Steroid Dehydrogenase Homolog

17β HSD13
- 17β-hydroxysteroid dehydrogenase type 13
- Short Chain Dehydrogenase/Reductase Family 16C Member 3
- Short-Chain Dehydrogenase/Reductase 9

17β HSD14
- 17β-hydroxysteroid dehydrogenase type 14
- Short Chain Dehydrogenase/Reductase Family 47C Member 1
- Retinal Short-Chain Dehydrogenase/Reductase RetSDR3
- Dehydrogenase/Reductase (SDR Family) Member 10
- Retinal Short-Chain Dehydrogenase/Reductase 3
- 17-Beta-Hydroxysteroid Dehydrogenase DHRS10

17β HSD15
- Retinol Dehydrogenase 11 (All-Trans/9-Cis/11-Cis)
- Androgen-Regulated Short-Chain Dehydrogenase/Reductase
- Short Chain Dehydrogenase/Reductase Family 7C, Member
- HCV Core-Binding Protein HCBP12
- Retinal Reductase 1 Prostate Short-Chain Dehydrogenase Reductase
- Prostate Short-Chain Dehydrogenase/Reductase
- Retinol Dehydrogenase 11

DHRS11
- Dehydrogenase/Reductase 11
- Short-Chain Dehydrogenase/Reductase Family 24C Member 1
- Dehydrogenase/Reductase (SDR Family) Member 11
- Inactive Dehydrogenase/Reductase Isoform

AKR1C1
- Aldo-Keto Reductase Family 1 Member C1
- 3α-hydroxysteroid dehydrogenases type IV
- 20α-hydroxysteroid dehydrogenase
- 20α-HSD
- 20α(3α)-HSD this is (stereo selective)
- Dihydrodiol Dehydrogenase 1
- Type II IV (3-Alpha-Hydroxysteroid Dehydrogenase
- Hepatic Dihydrodiol Dehydrogenase

AKR1C2
- Aldo-Keto Reductase Family 1 Member C2
- 3α-hydroxysteroid dehydrogenase type III
- Dihydrodiol Dehydrogenase 2
- Trans-1,2-Dihydrobenzene-1,2-Diol Dehydrogenase
- Pseudo-Chlordecone Reductase

AKR1C3/17β HSD5
- 17β-hydroxysteroid dehydrogenase type 5
- aldoketoreductase 1C3
- 3α-hydroxysteroid dehydrogenase type II
- Aldo-Keto Reductase Family 1 Member C3
- Prostaglandin F Synthase
- Trans-1,2-Dihydrobenzene-1,2-Diol Dehydrogenase
- Dihydrodiol Dehydrogenase Type I

AKR1C4
- Aldo-Keto Reductase Family 1 Member C4
- 3α-hydroxysteroid dehydrogenase type 1
- Chlordecone Reductase
- 3α-HSD
- Dihydrodiol Dehydrogenase Isozyme DD4
--Chlordecone Reductase

SRD5A1
- Steroid 5α-Reductase 1
- S5AR 1
- SR Type 1
- Steroid-5α-Reductase, α Polypeptide 1 (3-Oxo-5α-Steroid Delta 4-Dehydrogenase α1)
- 3-Oxo-5α-Steroid 4-Dehydrogenase 1

SRD5A2
- Steroid 5α-Reductase 2
- Steroid-5α-Reductase, α Polypeptide 2 (3-Oxo-5α-Steroid Delta 4-Dehydrogenase α2)
- Delta 4-Dehydrogenase α 1
- 3-Oxo-5α-Steroid 4-Dehydrogenase 2

SRD5A3
- Steroid 5α-Reductase 3
- SR Type 3
- Steroid 5-α-Reductase 2-Like
- Probable Polyprenol Reductase
- Polyprenol Reductase
- 3-Oxo-5α-Steroid 4-Dehydrogenase 3

STS
- Steroid Sulphatase
- arylsulphatase C
- Steroid Sulphatase (Microsomal), Isozyme S
- Steryl-Sulphate Sulphohydrolase
- Steryl-Sulphatase

SULT1E1
- Estrogen sulphotransferase
- Sulphotransferase Family 1E Member 1

SULT2A1
- Sulphotransferase Family 2A Member 1
- Sulphotransferase Family, Cytosolic, 2A, Dehydroepiandrosterone (DHEA)-Preferring, Member 1
- Dehydroepiandrosterone Sulphotransferase
- Bile-Salt Sulphotranasferase 2A1
- Hydroxysteroid Sulphotransferase

SULT2B1
- Sulphotransferase Family 2B Member 1
- Sulphotransferase Family, Cytosolic, 2B, Member 1
- Hydroxysteroid Sulphotransferase 2
- Alcohol Sulphotransferase

SULT1A1
- Sulphotransferase Family 1A Member 1
- Sulphotransferase Family, Cytosolic, 1A, Phenol-Preferring, Member 1
- Phenol-Sulphating Phenol Sulphotransferase 1
- Aryl Sulphotransferase 1
- Thermostable Phenol Sulphotransferase1
- Phenol Sulphotransferase 1

PANEL III: SYSTEMATIC REVIEW

The tissue expression of the various enzymes was searched systematically using PUBMED (https://www.ncbi.nlm.nih.gov) on 20 June 2018 using no time limitation.
The following terms were searched and the indicated number of papers were retrieved

	Search terms
	Lung *OR* lung cancer *OR* bone *OR* brain *OR* central nervous system *OR* pancreas *OR* esophagus *OR* stomach *OR* gastric *OR* small intestine *OR* duodenum *OR* jejunum *OR* ileum *OR* rectum *OR* colorectal *OR* gastro intestinal tract *OR* reproductive biology *OR* endometrium *OR* endometriosis *OR* endometrial cancer *OR* endo
AND	HSD17B1 *OR* 17beta-hydroxysteroid dehydrogenase type 1 *OR* steroid sulphatase *OR* steroid sulfatase *OR* 17beta-hydroxysteroid dehydrogenase type 2 *OR* 17beta-hydroxysteroid dehydrogenase type 3 *OR* 17beta-hydroxysteroid dehydrogenase type 4 *OR* 17beta-hydroxysteroid dehydrogenase type 5 *OR* 17beta-hydroxysteroid dehydrogenase type 6 *OR* 17beta-hydroxysteroid dehydrogenase type 7 *OR* 17beta-hydroxysteroid dehydrogenase type 8 *OR* 17beta-hydroxysteroid dehydrogenase type 9 *OR* 17beta-hydroxysteroid dehydrogenase type 10 *OR* 17beta-hydroxysteroid dehydrogenase type 11 *OR* 17beta-hydroxysteroid dehydrogenase type 12 *OR* 17beta-hydroxysteroid dehydrogenase type 13 *OR* 17beta-hydroxysteroid dehydrogenase type 14 *OR* 17beta-hydroxysteroid dehydrogenase type 15 *OR* HSD17B1 *OR* HSD17B2 *OR* HSD17B3 *OR* HSD17B3 *OR* HSD17B4 *OR* HSD17B5 *OR* HSD17B6 *OR* HSD17B7 *OR* HSD17B8 *OR* HSD17B9 *OR* HSD17B10 *OR* HSD17B11 *OR* HSD17B12 *OR* HSD17B13 *OR* HSD17B14 *OR* HSD17B15 OR SULT1E1 *OR* steroid sulphotransferase *OR* steroid sulfotransferase OR estrogen sulphotransferase *OR* estrogen steroid sulfotransferase *OR* intracrinology *OR* SULT1E1 *OR* SULT2A1 *OR* 5alpha-reductase type 1 *OR* 5alpha-reductase type 2 *OR* 5alpha-reductase type 3 *OR* SRD5A1 *OR* SRD5A2 OR SRD5A3 *OR* 3beta-hydroxysteroid dehydrogenase TYPE 1 *OR* 3beta-hydroxysteroid dehydrogenase TYPE 2 *OR* HSD3B1 *OR* HSD3B2 *OR* AKR1C1 *OR* AKR1C2 *OR* AKR1C3 *OR* AKR1C4 *OR* AROMATASE *OR* CYP19A1 *OR* CYP11A1 *OR* CYP17A1
NOT	Cell lines *OR* cell line
Filter	Humans & English
→	**TOTAL RETRIEVED PAPERS: 2971 (published from 1962)**

After removing reviews, these papers were further filtered manually as shown in the scheme below (left), based on title, abstract of keywords. Finally 192 studies were thoroughly considered for inclusion or exclusion (right), after which selection, 172 papers were included in the present study (papers selected through systematic analysis are reported in Tables VI-VIII).

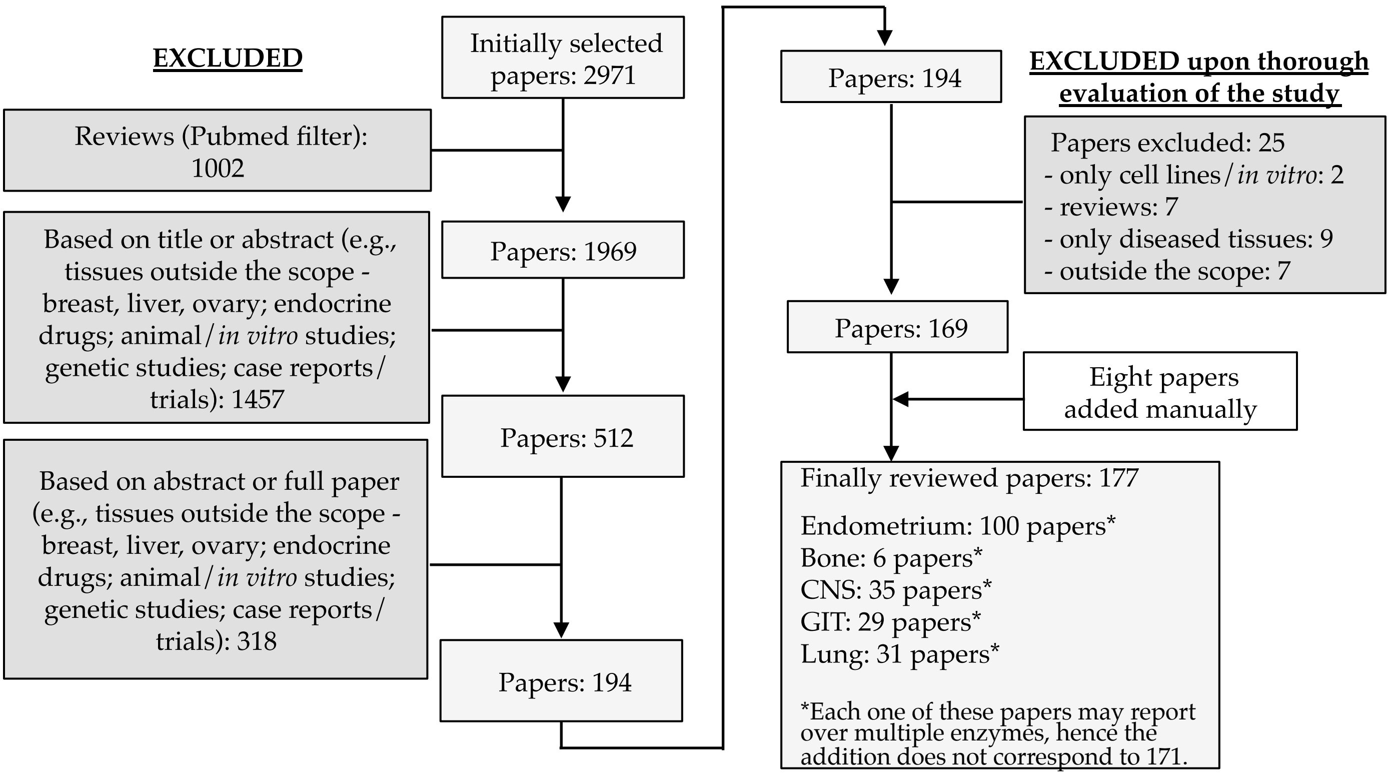

Supplement: Supplementary file 1 [file Presentation_1.pdf]
